# Targeting metabolic vulnerabilities: REV-ERB agonist SR9009 potentiates sorafenib efficacy in liver cancer

**DOI:** 10.1038/s41420-026-02940-3

**Published:** 2026-01-19

**Authors:** Silvia Sabbioni, Paola Guerriero, Ram C. Shankaraiah, Laura Masatti, Angelo Michilli, Cristian Bassi, Lucilla D’Abundo, Farzaneh Moshiri, Bruno De Siena, Edi Simoni, Laura Astolfi, Laura Gramantieri, Roberta Roncarati, Bahaeldin K. Elamin, Massimo Bonora, Paolo Pinton, Carlo M. Croce, Massimo Negrini, Elisa Callegari

**Affiliations:** 1 Department of Life Sciences and Biotechnology, University of Ferrara, 44121 Ferrara, Italy; 2 Laboratorio Per Le Tecnologie Delle Terapie Avanzate (LTTA), University of Ferrara, 44121 Ferrara, Italy; 3 Department of Translational Medicine, University of Ferrara, 44121 Ferrara, Italy; 4 Bioacoustics Research Laboratory, Department of Neurosciences, University of Padova, 35129 Padova, Italy; 5 Division of Internal Medicine, Hepatobiliary and Immunoallergic Diseases, IRCCS Azienda Ospedaliero-Universitaria di Bologna, 40138 Bologna, Italy; 6 CNR Institute of Molecular Genetics “Luigi Luca Cavalli-Sforza”, Unit of Bologna, Bologna, Italy; 7 Department of Microbiology and Clinical Parasitology, College of Medicine, University of Bisha, Bisha, 61922 Saudi Arabia; 8 Department of Medical Microbiology, Faculty of Medical Laboratory Sciences, University of Khartoum, Khartoum, Sudan; 9 Department of Medical Sciences, Section of Experimental Medicine, University of Ferrara, 44121 Ferrara, Italy; 10 Department of Cancer Biology and Genetics, The Ohio State University, Columbus, OH USA; 11 Present Address: Glaxo Smith Kline - Wavre Belgium Av. Fleming 20, 1300 Wavre, Belgium; 12 Present Address: Department of Biology, University of Padova, 35100 Padova, Italy

**Keywords:** Cancer, Oncogenesis

## Abstract

Hepatocellular carcinoma (HCC) is one of the most common cancers and the third leading cause of cancer-related death worldwide. The prognosis is poor, with a median survival of 12–15 months in patients with advanced-stage disease. Early diagnosis and the development of new, more effective therapeutic strategies are needed to address the challenges posed by this malignancy. Although immune checkpoint inhibitors have replaced multikinase inhibitors as first-line therapy, sorafenib continues to represent a valuable option for patients with contraindications to newer treatments. Based on genome-wide RNA-seq analysis, which identified mitochondrial oxidative phosphorylation (OxPhos) and *Hmox1* upregulation as potential pro-survival mechanisms in sorafenib-resistant cells, we investigated whether SR9009, a synthetic agonist of the nuclear receptor REV-ERBα/β, heme competitor, and inhibitor of mitochondrial respiration, could enhance the antitumor efficacy of sorafenib in liver cancer models. Co-treatment with SR9009 and sorafenib significantly enhanced cytotoxic effects in both mouse and human liver cancer cells. This synergistic activity was associated with increased levels of free heme and a complete inhibition of mitochondrial OxPhos. In vivo xenograft studies confirmed that the combination was effective even in sorafenib-resistant tumors. Furthermore, in a N-Nitrosodiethylamine (DEN)-induced HCC model, the combination therapy led to a reduction in size in over 90% of tumor nodules, representing a significant improvement over sorafenib alone. The combination was well tolerated, with no evident signs of acute toxicity. These findings support the concept that the efficacy of anticancer therapies can be enhanced by targeting the metabolic adaptations that tumor cells rely on for survival. Combining sorafenib with agents like SR9009, that disrupt metabolic homeostasis, may offer a promising strategy for treating advanced HCC.

## Introduction

With more than 800,000 cases diagnosed each year, hepatocellular carcinoma (HCC) is the sixth most common cancer and the third leading cause of cancer death worldwide [1]. The prognosis of HCC depends on tumor stage, with a survival rate greater than 70% at 5 years for early-stage HCC patients and a median survival of 12–15 months for advanced-stage patients treated with systemic therapies. Hence, early diagnosis and new and more effective therapeutic approaches are needed.

Therapeutic treatment depends on the stage of cancer, as established according to the Barcelona Clinic Liver Cancer (BCLC) system. For over a decade, the treatment of advanced HCC has been limited to sorafenib [2], a multikinase and antiangiogenic inhibitor. In 2018, lenvatinib, another multikinase inhibitor, was shown to be non-inferior to sorafenib as a first-line therapy [3]. In 2020, the combination of the anti-PD-L1 atezolizumab and anti-VEGF agents led to a change in the first-line standard for all HCC patients eligible for systemic treatment, obtaining the longest median OS of any first-line treatment for advanced-stage HCC to date [4, 5]. Unfortunately, only about 30% of patients with HCC respond or are eligible for immunotherapy, depending on clinical background, treatment tolerance, and the occurrence of immune-related adverse events [6, 7]. Hence, sorafenib remains a first-line therapy option in patients with contraindications for immunotherapy [8, 9]. Nevertheless, it shows limitations in term of response and survival, mainly due to early occurrence of resistance. A number of studies have indeed focused on mechanisms of acquired sorafenib resistance. Metabolic and immunological adaptations have been related to this phenomenon [10].

Here, we analyzed the expression profiles of cells that developed resistance to sorafenib in comparison to the susceptible counterpart. The analyses revealed mechanisms that were potentially targetable. Specifically, we evaluated the activity of SR9009 [11], a synthetic agonist of the nuclear receptors REV-ERBα and REV-ERBβ, transcriptional repressors that regulate metabolic pathways linked to the circadian rhythm [12–14]. The heme group is the physiological ligand of both receptors and is required for their repressor activity [15]. REV-ERBα plays a repressive role in cell proliferation and metabolism [16] and is frequently downregulated in several types of cancer [14]. It was also reported that SR9009 can exert cytotoxic effects on several tumor cell lines in vitro and antitumor effects in vivo [17, 18]. SR9009 has been described to have specific lethal effects on tumor cells and oncogene-induced senescent cells but not on normal cells [18]. Furthermore, SR9009 has been reported to reduce mouse embryonic stem cell viability and proliferation, by inhibiting mitochondrial respiration in a REV-ERB independent manner [19].

In this scenario, agents such as SR9009 might be suitable for combined approaches with therapies already approved for clinical use. Here, we tested the ability of the combination of SR9009 with sorafenib to overcome resistance mechanisms and improve antitumor efficacy in in vitro and in vivo liver cancer models.

## Results

### Increased mitochondrial activity acts as a pro-survival mechanism to cope with the effects of sorafenib

Understanding mechanisms that make cancer cells insensitive to sorafenib can potentially reveal attributes that can be operated to improve its anticancer efficacy. To this end, we have previously described the development of Hep55.1C murine hepatoma cells that are nonresponsive to sorafenib (H55-RES) [20]. Nonresponsive cells grow in vitro in the presence of 10 μM sorafenib, which normally causes wild-type cells to die within a few days. Furthermore, they can form tumors of comparable size both in the presence or absence of sorafenib-based therapy [20].

By applying *Gene Set Enrichment Analysis* (GSEA) algorithm to genome-wide RNA-seq data from sorafenib-susceptible versus nonsusceptible cells, we identified a number of molecular pathways (pathway gene sets were derived from the Reactome Path Database) significantly enriched in upregulated genes of the nonsusceptible cells (Table 1).

**Table 1:** Top pathways enriched in upregulated genes as identified by Gene Set Enrichment Analysis (GSEA).

Reactome/hallmark pathways enriched in resistant vs. WT Hep55.1 C cells	Enrichment score	Normalized enrichment score	*p* value	FDR
REACTOME_TRANSLATION	0.634	4.195	<0.0001	<0.0001
REACTOME_EUKARYOTIC_TRANSLATION_INITIATION	0.733	4.115	<0.0001	<0.0001
HALLMARK_OXIDATIVE_PHOSPHORYLATION	0.617	3.835	<0.0001	<0.0001
REACTOME_NEGATIVE_REGULATION_OF_NOTCH4_SIGNALING	0.769	3.760	<0.0001	<0.0001
REACTOME_RESPIRATORY_ELECTRON_TRANSPORT_ATP_SYNTHESIS_BY_CHEMIOSMOTIC_COUPLING_AND_HEAT_PRODUCTION_BY_UNCOUPLING_PROTEINS	0.612	3.706	<0.0001	<0.0001
REACTOME_EUKARYOTIC_TRANSLATION_ELONGATION	0.725	3.685	<0.0001	<0.0001
REACTOME_RESPIRATORY_ELECTRON_TRANSPORT	0.653	3.524	<0.0001	<0.0001
REACTOME_CYTOPROTECTION_BY_HMOX1	0.607	3.486	<0.0001	<0.0001
REACTOME_CELLULAR_RESPONSE_TO_HYPOXIA	0.649	3.472	<0.0001	<0.0001
REACTOME_METABOLISM_OF_POLYAMINES	0.669	3.340	<0.0001	<0.0001

All the enriched molecular pathways play important roles in cell growth and survival. The enrichment of upregulated genes within the gene sets related to “electron transport chain”, “oxidative phosphorylation”, and “ATP synthesis” suggested that the increase in the expression of genes involved in mitochondrial activity represents a response that cells activate to counterbalance conditions that compromise mitochondrial function. In fact, it has been reported that in addition to its well-known multikinase inhibitor and antiangiogenic activities, sorafenib can also inhibit mitochondrial function, as reported in published studies [21]. These pathways were not only enriched in upregulated genes, but the upregulation was statistically significant, as shown for example by the genes belonging to the gene set HALLMARK_OXIDATIVE_PHOSPHORYLATION (Supplementary Table 1). Therefore, increased expression of genes implicated in mitochondrial oxidative phosphorylation (OxPhos) represents a major pro-survival mechanism to cope with the action of sorafenib.

This finding was also supported by the evaluation of the biological processes influenced by sorafenib during the first hours of treatment. According to the analysis of the genes positively modulated by sorafenib in a time course study of RNA-seq data, Gene Ontology (GO) analysis revealed that the upregulated genes involved in mitochondrial activity began to increase during the first hours of treatment and further increased in cells resistant to sorafenib (Supplementary Fig. 1 and Supplemantary Table 2). These findings indicate that sorafenib leads to the induction of adaptive transcriptional mechanisms that, over time, can reach levels that make cells insensitive to the drug. Among these, the increased expression of genes implicated in mitochondrial functionality appears to be one of the most relevant.

### The sorafenib/SR9009 combination shows a synergistic effect on mouse hepatoma cell viability

These results suggested that pharmacological treatments capable of acting on OxPhos could inhibit this pro-survival response and possibly enhance the antitumor effect of sorafenib. Among drugs, we focused on SR9009, an agonist of REV-ERBα/β, a nuclear receptor that is a component of circadian clock-modulating proteins [22]. Indeed, SR9009 has been also reported to exert a cytotoxic effect on tumor cells not only through the inhibition of autophagy and de novo lipogenesis [17, 18] but also through the reduction of mitochondrial respiration [19].

**Fig. 1:**
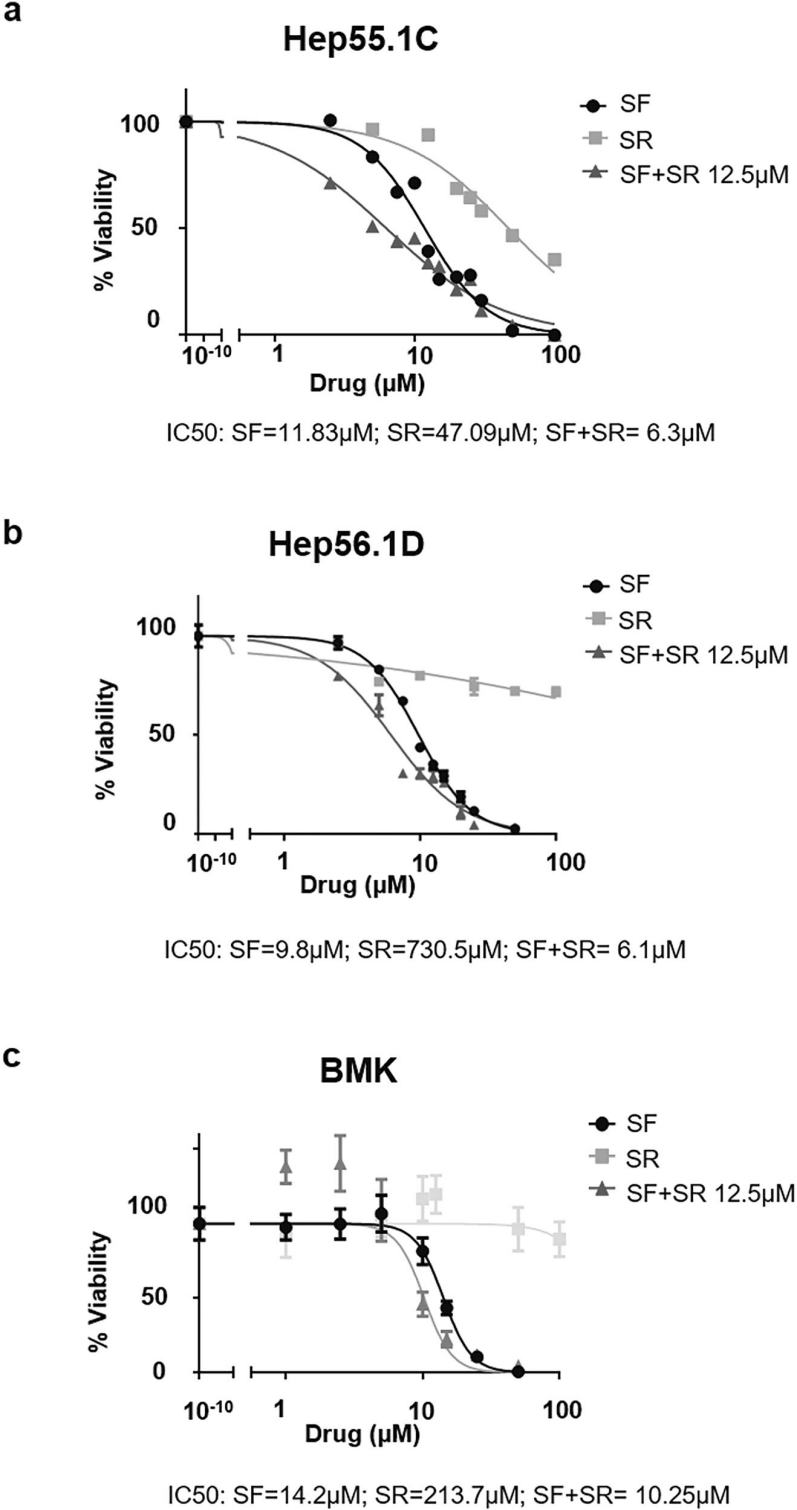
The combination of sorafenib and SR9009 reduces the IC₅₀ of sorafenib in murine hepatoma cell lines. Dose–response curves were generated to evaluate the cytotoxic effects of increasing concentrations of sorafenib (SF) and SR9009 (SR) in Hep55.1 C (**a**) and Hep56.1D (**b**) hepatoma cells. When administered as a single agent, SR9009 exhibited cytotoxicity only at high concentrations (IC₅₀ = 47.1 µM in Hep55.1C and IC₅₀ = 730.5 µM in Hep56.1D cells). Drug combination studies were performed using increasing concentrations of sorafenib in the presence of a fixed dose of SR9009 (12.5 µM). After 48 h of treatment, a pronounced synergistic effect on cell viability was observed, with a marked reduction in sorafenib IC₅₀ values (from 12 µM to 6 µM in Hep55.1C and from 10 µM to 6 µM in Hep56.1D), and a detectable cytotoxic response even at submicromolar sorafenib concentrations (<1 µM). In contrast, in normal baby mouse kidney fibroblasts (BMK) (**c**), low concentrations of SR9009 slightly improved cell growth and viability, while cytotoxic effects were evident only at high doses (>100 µM). When combined with SR9009 (12.5 µM), sorafenib showed only a modest reduction in IC₅₀ (from 14 µM to 10 µM), with no evidence of synergistic cytotoxicity at lower concentrations. Cell viability values were normalized to untreated controls. Drug concentrations are expressed on a log₁₀ scale. Data points represent mean cell viability ± SD for each concentration.

As shown in Fig. 1, the combination of SR9009 with sorafenib exerted a marked cytotoxic effect in two distinct murine hepatoma cell lines, Hep55.1C and Hep56.1D, which differ in their TP53 gene status. When administered as a single agent, SR9009 displayed minimal cytotoxicity in both cell lines, except at the highest concentrations tested. In contrast, co-administration of 12.5 µM SR9009 with sorafenib significantly enhanced sorafenib sensitivity, reducing its IC₅₀ from 12 µM to 6 µM in Hep55.1C cells and from 10 µM to 6 µM in Hep56.1D cells. Notably, cell viability declined sharply even at low sorafenib concentrations, suggesting a synergistic interaction between the two agents (Fig. 1a, b and Supplementary Fig. 2).

To further evaluate long-term proliferative capacity under combined treatment, we performed a colony formation assay in Hep55.1C cells. The combination of SR9009 and sorafenib markedly reduced both the number and the size of colonies compared with either treatment alone (Supplementary Fig. 3). These findings were corroborated by synergy analysis using the SynergyFinder algorithm (http://www.synergyfinderplus.org/#!/) (Supplementary Fig. 4).

In contrast, only a modest reduction in viability was observed in normal baby mouse kidney fibroblasts (BMKs) treated with the SR9009-sorafenib combination (Fig. 1c), indicating that the synergistic cytotoxic effects were largely selective for cancer cells. These findings are consistent with previous reports showing that SR9009 mitigates hepatic steatosis and inflammation [23], suppresses tumor cell proliferation and migration without affecting normal hepatocytes [24], and can even attenuate the hepatotoxicity of anticancer agents [25]. We then assessed whether the combination of sorafenib and SR9009 could also be effective in sorafenib-resistant H55-RES cells. Cells were treated with different concentrations of sorafenib in the presence or absence of SR9009 (Supplementary Fig. 5). These results demonstrated that SR9009 was able to sensitize cells that were previously insensitive to sorafenib.

We next assessed whether the combination of sorafenib and SR9009 was effective in human HCC cells. Across four human HCC cell lines, the combination consistently produced greater cytotoxicity than sorafenib alone (Supplementary Fig. 6). This confirms that the synergistic interaction between SR9009 and sorafenib is preserved in human models. PARP cleavage and GSEA analyses further demonstrated enhanced apoptotic signaling in cells treated with the combination compared with either monotherapy (Supplementary Fig. 7 and Supplementary Table 3).

### Mitochondrial function is strongly inhibited by the combination of sorafenib and SR9009

**Fig. 2:**
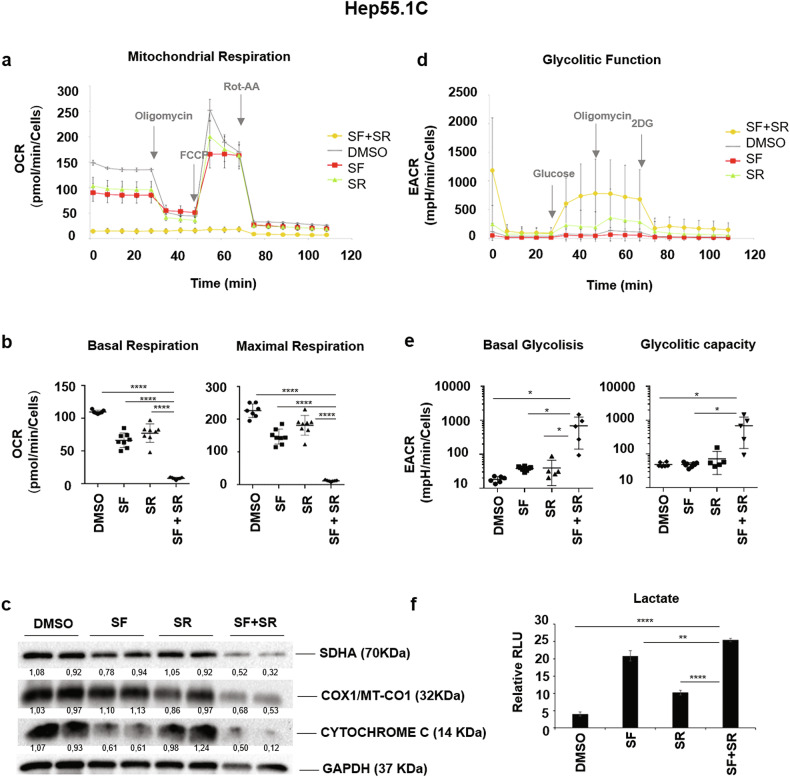
Combined treatment with sorafenib and SR9009 markedly inhibits mitochondrial ATP production in Hep55.1C cells. Mitochondrial oxidative phosphorylation (OxPhos) and glycolytic activity were assessed in Hep55.1C cells under the following conditions: (i) sorafenib (7.5 µM; SF), (ii) SR9009 (12.5 µM; SR), (iii) combined sorafenib + SR9009 (SF + SR), and (iv) DMSO control. **a** OxPhos activity was measured as oxygen consumption rate (OCR) using a Seahorse XFe/XF Extracellular Flux Analyzer under basal conditions and following sequential injections of oligomycin, FCCP (carbonyl cyanide-4-(trifluoromethoxy)phenylhydrazone), and a rotenone/antimycin A mixture (Rot/AA). **b** Both basal and maximal respiration were profoundly reduced in cells treated with SF + SR, indicating severe mitochondrial dysfunction. **c** Immunoblot analysis confirmed a marked decrease in mitochondrial complex proteins, including the succinate dehydrogenase subunit A (SDHA), cytochrome c oxidase subunit I (COX1/MT-CO1), and cytochrome c. Protein levels were normalized to GAPDH and expressed relative to untreated controls. **d** Glycolytic flux was measured as extracellular acidification rate (ECAR). **e** Cells treated with SF + SR exhibited enhanced glycolytic activity compared to single-agent treatments, suggesting a compensatory shift toward glycolysis to sustain residual ATP production under conditions of mitochondrial inhibition. Both basal glycolysis and glycolytic capacity were significantly elevated, indicating that glycolysis operated near its maximal rate in the combination setting. **f** Consistently, lactate production was significantly increased in SF + SR-treated cells compared with control and single-drug treatments. Data represent mean ± SD. *p ≤ 0.05; **p ≤ 0.01; ***p ≤ 0.001; ****p ≤ 0.0001.

Considering the previous results and that the inhibitory effects of either SR9009 or sorafenib on mitochondrial functionality have been previously described [19, 21], to investigate the mechanism underlying the synergistic cytotoxic effect, we analyzed whether the combination treatment had an impact on mitochondrial metabolism. We evaluated the two main metabolic fluxes, oxidative phosphorylation (OxPhos) and glycolysis, in the two murine cell lines Hep55.1C and Hep56.1D using the Seahorse platform [26]. Figure 2 shows the results of the OCR (oxygen consumption rate) and ECAR (extracellular acidification rate) activities of single agents and combined treatments in Hep55.1C cells. A slight reduction in ATP production via OxPhos was caused by both sorafenib and SR9009 as single agents, while strong inhibition was detected after treatment with the combination of sorafenib and SR9009 (Fig. 2a, b). Consistently, a marked decrease in the expression of mitochondrial respiratory complex proteins such as SDHA (Succinate Dehydrogenase subunit A), COX1 (Cytochrome c oxidase I) and Cytochrome c was observed following the combined treatment (Fig. 2c), further supporting a synergistic impairment of mitochondrial function.

The inhibition of oxidative phosphorylation (OxPhos) was partially compensated by an increase in glycolytic flux (Fig. 2d, e), as evidenced by a significant elevation in lactate production in sorafenib + SR9009 treated cells compared with either single-agent treatment or control (Fig. 2f). Similar metabolic reprogramming was observed in Hep56.1D cells, confirming the reproducibility of this response across distinct hepatoma models (Supplementary Fig. 8).

Mitochondrial function was further evaluated using the Mitochondrial ToxGlo Assay, which simultaneously measures cellular ATP content and membrane integrity. Among all tested murine and human liver cancer cell lines, the combination of SR9009 and sorafenib produced the most pronounced reduction in ATP levels (Supplementary Fig. 9), indicating a marked impairment of mitochondrial activity. To validate this observation, we assessed the effects of sorafenib combined with the mitochondrial respiration inhibitor IACS-010759 [27]. This combination elicited a cytotoxic response in HCC cells comparable to that induced by SR9009 and sorafenib (Supplementary Fig. 10). Collectively, these findings indicate that the synergistic inhibition of mitochondrial function represents a major mechanism underlying the cytotoxic effects of the SR9009, sorafenib combination in vitro.

## SR9009 modulates intracellular heme homeostasis and oxidative stress in sorafenib-resistant cells

**Fig. 3:**
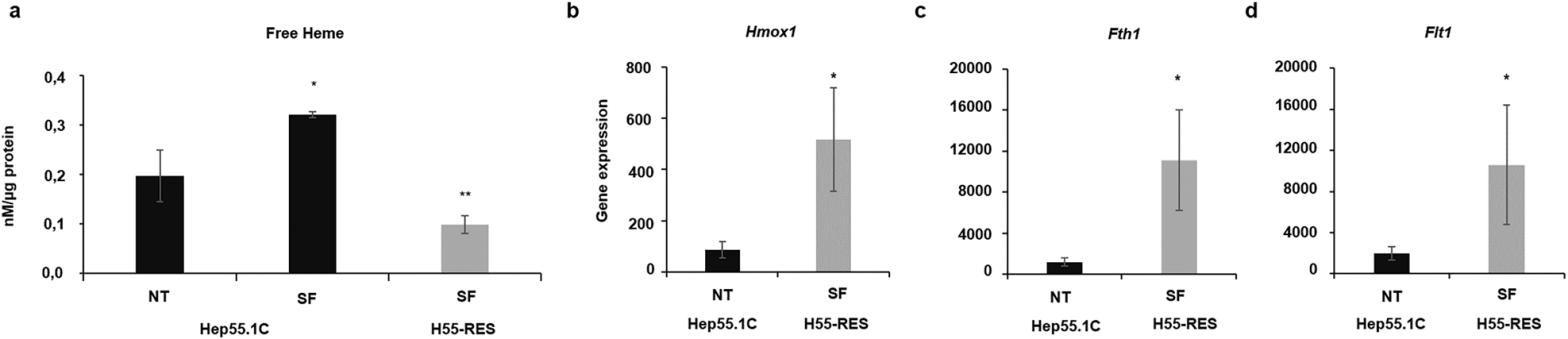
Sorafenib-resistant hepatoma cells are protected from heme-induced toxicity through upregulation of *Hmox1* and *ferritin* genes. **a** Intracellular free heme levels were quantified in cell lysates using a functional assay based on inactive apo-horseradish peroxidase (see *Materials and Methods*). Treatment of wild-type Hep55.1C cells with sorafenib (SF) led to a pronounced accumulation of free heme compared to untreated (NT) controls. In contrast, sorafenib-resistant H55-RES cells, continuously maintained in the presence of SF, exhibited significantly lower free heme levels, indicating activation of protective mechanisms against sorafenib-induced heme toxicity. **b** RNA-seq analysis revealed a strong upregulation of *Hmox1*, encoding heme oxygenase-1 (HO-1), in resistant cells, suggesting a pivotal role for this enzyme in mitigating toxic heme accumulation. **c**, **d** Consistently, *Fth1* (ferritin heavy chain 1) and *Ftl1* (ferritin light chain) transcripts were significantly elevated in resistant cells compared with wild-type Hep55.1C cells, supporting enhanced sequestration of labile ferrous iron (Fe²⁺) and attenuation of iron-mediated cytotoxicity. Data represent mean ± SD. *p ≤ 0.05; **p ≤ 0.01.

Transcriptomic comparison between wild-type and sorafenib-resistant Hep55.1C cells revealed a significant enrichment of upregulated genes associated with the HMOX1 signaling pathway (“CYTOPROTECTION MEDIATED BY HMOX1”) (Table 1). Consistently, *Hmox1*, which encodes heme oxygenase-1 (HO-1), the key enzyme catalyzing the degradation of free heme into biliverdin and bilirubin, was markedly upregulated in resistant cells (Fig. 3b). At the protein level, HO-1 expression was likewise elevated (Supplementary Fig. 11). Correspondingly, intracellular free heme levels were significantly reduced in resistant cells compared with sorafenib-treated wild-type cells (Fig. 3a), suggesting that HO-1 upregulation mitigates heme-induced cytotoxicity. Functional validation through siRNA-mediated *Hmox1* knockdown in resistant Hep55.1C cells resulted in a dose-dependent reduction of HO-1 protein levels, leading to impaired cell viability and mitochondrial activity (Supplementary Fig. 12). Moreover, the ferrous iron (Fe²⁺) released during heme degradation appeared to be detoxified through the coordinated upregulation of ferritin heavy (*Fth1*) and light (*Ftl*) chain genes (Fig. 3c, d).

**Fig. 4:**
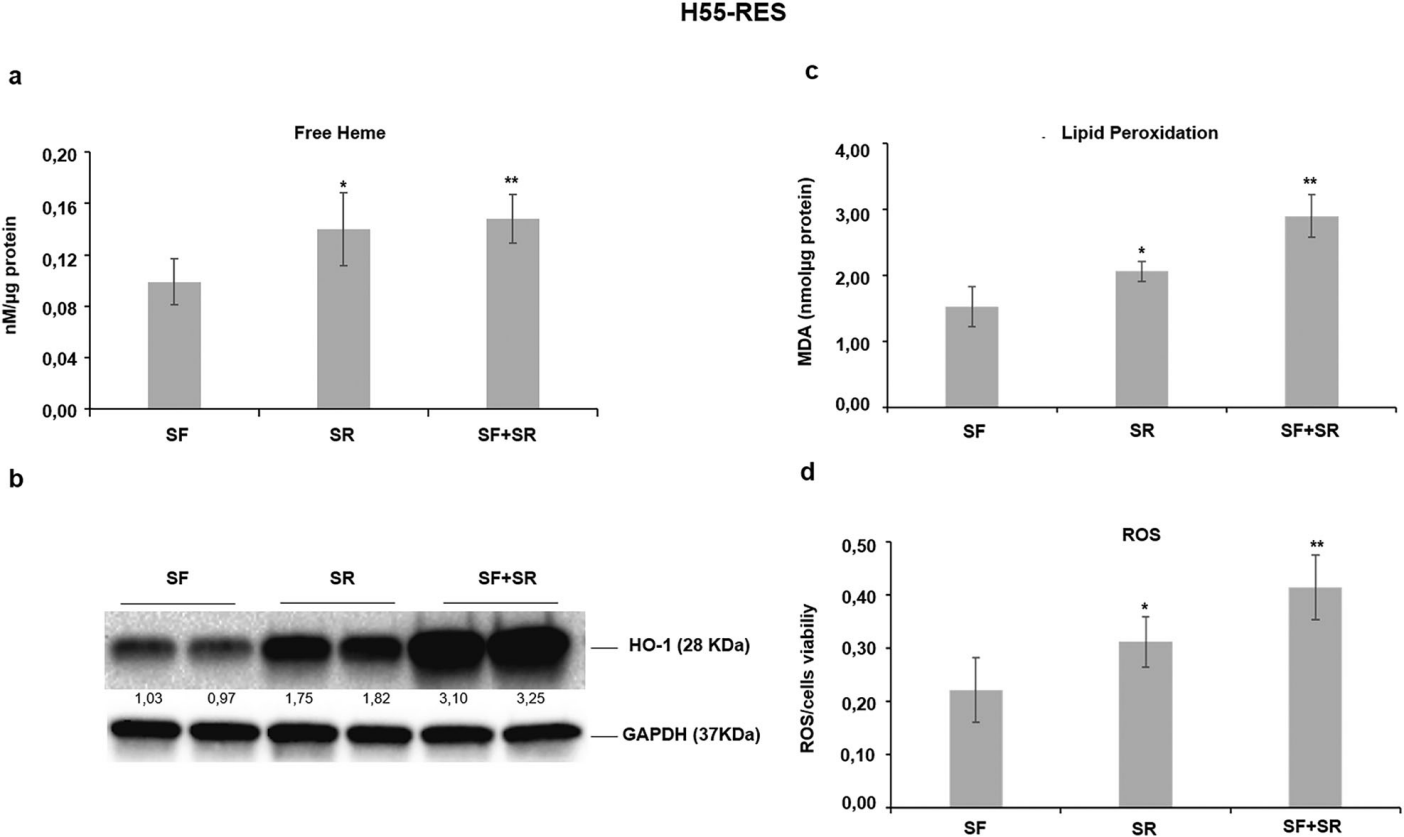
SR9009 increases free heme levels and promotes oxidative stress in sorafenib-resistant hepatoma cells. **a** In H55-RES cells, treatment with SR9009 markedly increased intracellular free heme levels, both when administered alone (SR) and in combination with sorafenib (SR + SF). **b** In response to elevated heme accumulation, heme oxygenase-1 (HO-1) protein expression was concomitantly upregulated following SR9009 treatment, with a further induction observed under combined SR9009 and sorafenib exposure. **c**, **d** To evaluate SR9009-induced oxidative stress, lipid peroxidation and intracellular reactive oxygen species (ROS) levels were quantified in H55-RES cells treated with sorafenib (SF), SR9009 (SR), or the combination (SR + SF). Malondialdehyde (MDA) content, a marker of lipid peroxidation, was measured in cell lysates after 24 h of treatment, while ROS accumulation was quantified in live cells and normalized to cell viability. Data represent mean ± SD. *p ≤ 0.05; **p ≤ 0.01.

Based on these observations, considering that SR9009 is an heme agonist, we hypothesized that SR9009 may compete with heme for binding to REV-ERBα/β and potentially to other heme-binding proteins, including mitochondrial cytochromes. To test this, we measured intracellular free heme levels and assessed the resulting impact on the cellular redox state in sorafenib-resistant H55-RES cells. SR9009 treatment led to a marked increase in free heme accumulation, which was accompanied by elevated lipid peroxidation and reactive oxygen species (ROS) production, effects that were further amplified when SR9009 was combined with sorafenib (Fig. 4a, c, d). These findings suggest that SR9009-induced perturbation of heme homeostasis plays a key role in promoting mitochondrial dysfunction and cellular cytotoxicity under combination treatment.

Consistent with a compensatory response to oxidative stress, HO-1 protein levels were further increased following treatment with SR9009 and sorafenib (Fig. 4b). A similar induction of HO-1 was also observed in Hep55.1 C cells exposed to the combination treatment (Supplementary Fig. 13), further supporting the role of heme dysregulation and redox imbalance in the cytotoxic mechanism of SR9009-sorafenib co-treatment.

### Effect of the combination of sorafenib and SR9009 on Hep55.1C cells in vivo

**Fig. 5:**
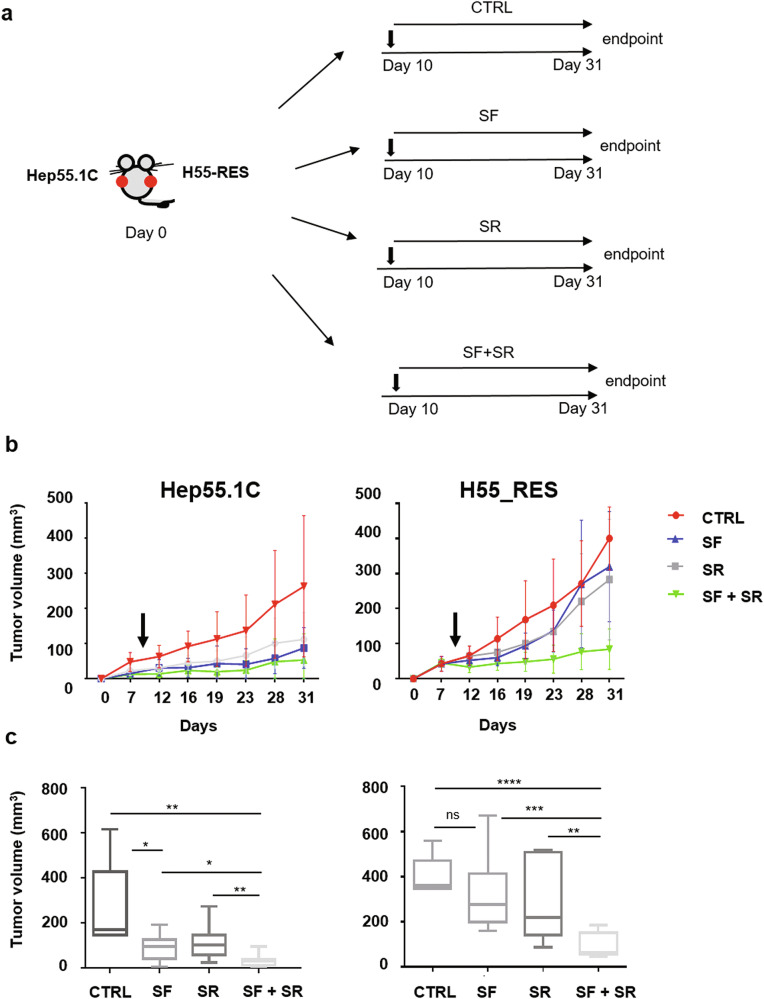
SR9009 potentiates the antitumor efficacy of sorafenib in sorafenib-resistant xenograft models. **a** Wild-type Hep55.1C cells and sorafenib-resistant (H55-RES) cells were subcutaneously implanted into the left and right flanks, respectively, of female C57BL/6 mice. **b** Approximately 10 days after cell implantation, when tumor volumes reached ~50 mm³, mice were randomized into four treatment groups: (1) vehicle control (CTRL, *n* = 5), (2) sorafenib alone (SF, *n* = 10), (3) SR9009 alone (SR, *n* = 10), and (4) sorafenib plus SR9009 (SF + SR, *n* = 10). Treatments were administered as indicated, and tumor volumes were measured every two days until endpoint. **c** Mean tumor volumes at endpoint are shown. Data are presented as mean ± SD. *p ≤ 0.05; **p ≤ 0.001.

We investigated whether the effects on tumor cell viability could be reproduced in vivo. Wild-type Hep55.1C cells and sorafenib-resistant H55-RES cells were implanted subcutaneously into opposite flanks of 4-week-old female syngeneic C57BL6 mice (Fig. 5a). Ten days post-implantation, when palpable tumors had formed, animals were randomly assigned to four treatment groups (*n* = 10 per group): (i) sorafenib (30 mg/kg), (ii) SR9009 (100 mg/kg), (iii) sorafenib + SR9009, or (iv) vehicle as control. As shown in Fig. 5b, c, all treatments were effective against wild-type cells. In contrast, only the sorafenib + SR9009 combination demonstrated marked antitumor efficacy in mice bearing H55-RES xenografts, indicating that SR9009 effectively restores sensitivity to sorafenib in resistant tumors.

Mechanistically, tumors from the combination group displayed a pronounced reduction in mitochondrial respiratory complex proteins and a robust induction of HO-1 expression, compared with either monotherapy (Supplementary Fig. 14). These findings corroborate in vitro results, supporting a model in which combined sorafenib and SR9009 treatment drives mitochondrial dysfunction and oxidative stress–mediated tumor regression in resistant HCC.

**Fig. 6:**
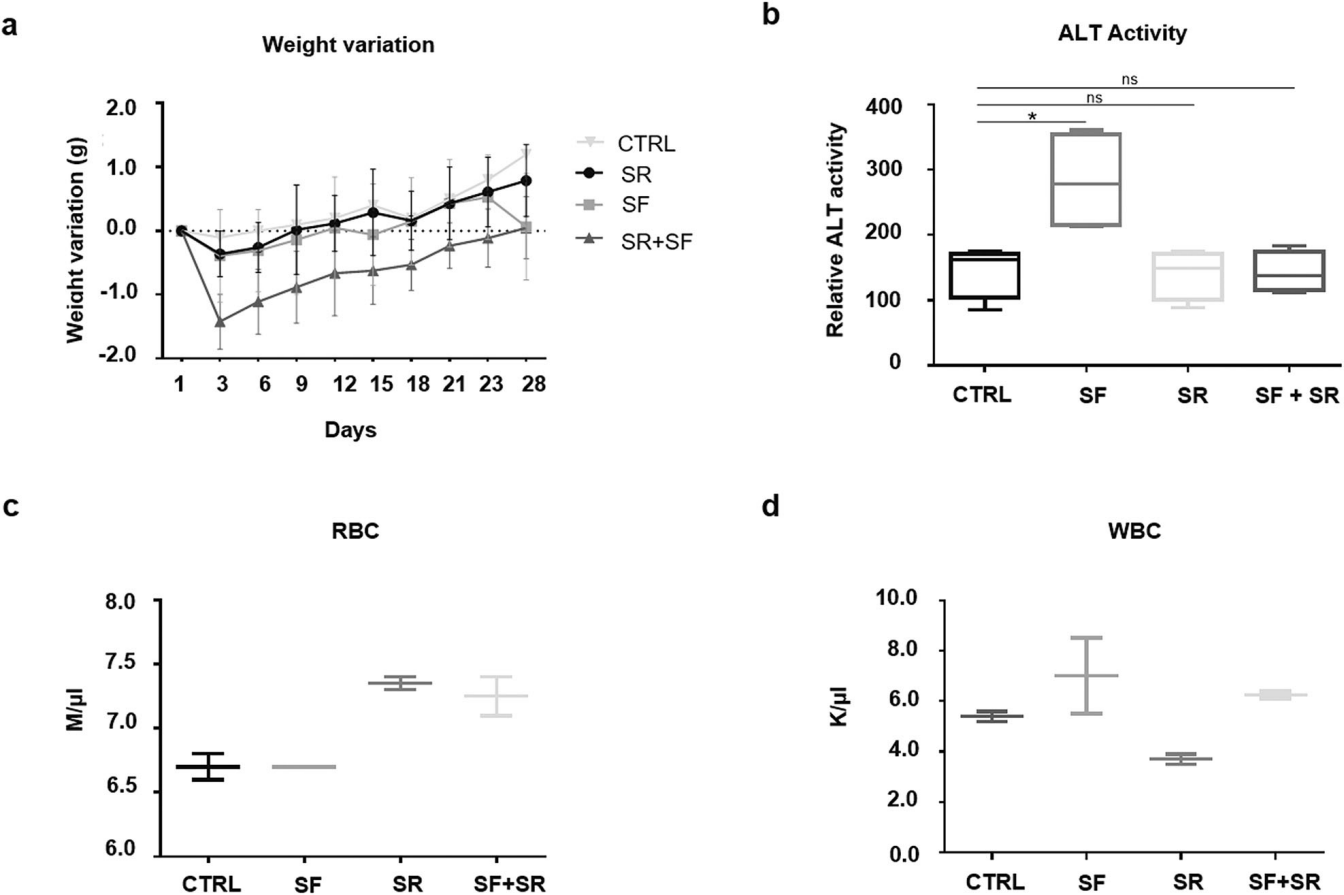
In vivo toxicity assessment of the sorafenib and SR9009 combination. **a** Mouse body weight was monitored throughout the treatment period. Except for a transient decrease during the initial days of combination treatment, no significant weight loss was observed in any treatment group. **b** Liver function was evaluated by measuring serum alanine aminotransferase (ALT) activity. As expected, ALT levels were elevated following sorafenib treatment alone but remained within the normal range when SR9009 was administered, either alone or in combination with sorafenib, indicating a protective effect of SR9009 on liver function. **c**, **d** Hematological parameters, including red blood cell (RBC) and white blood cell (WBC) counts, were not significantly altered under any treatment condition. Data are presented as mean ± SD. Treatment groups: control (CTRL), SR9009 alone (SR), sorafenib alone (SF), and the combination (SR + SF). *p ≤ 0.05.

In vivo studies were also useful to investigate toxic side effects (Fig. 6). No significant weight loss was detected in any of the treatment regimens. A slight weight reduction was observed in the sorafenib + SR9009combination group during the first week, which recovered during the following two weeks. Hepatotoxicity was observed after treatment with sorafenib but not with SR9009. In fact, blood alanine transferase (ALT) levels normalized when sorafenib was combined with SR9009. Finally, hematological counts were not significantly altered. Therefore, the combination of sorafenib and SR9009 was more effective than sorafenib as a single agent, and no increase in toxicity was detected.

### Antitumor efficacy of sorafenib combined with SR9009 in the TG221 HCC model

**Fig. 7:**
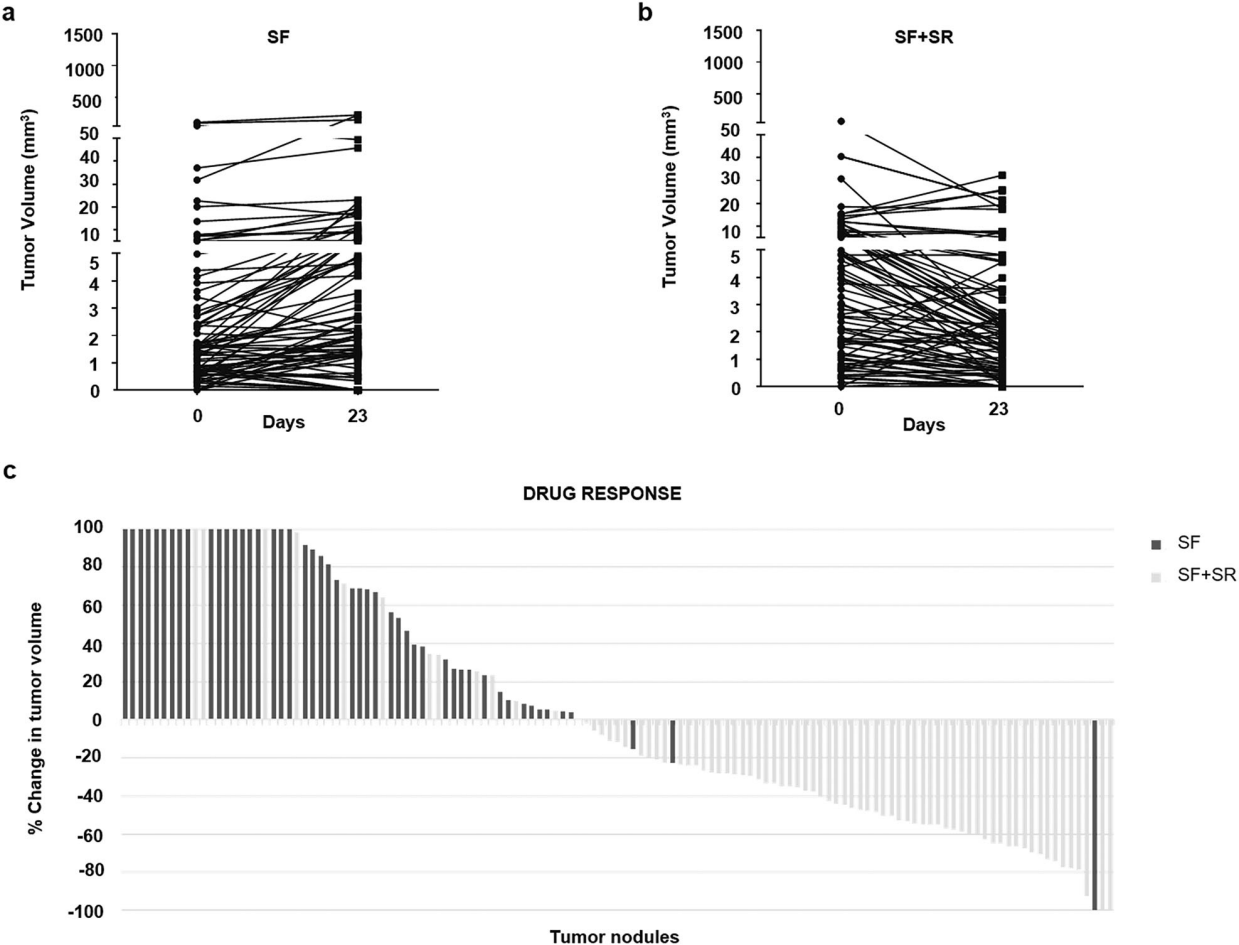
The combination of sorafenib and SR9009 shows superior efficacy to sorafenib alone in primary liver tumors. At six months of age, thirty DEN-treated TG221 male mice were randomized into two groups and treated daily for 23 days with either sorafenib alone (SF, 10 mg/kg; *n* = 15) or the combination of sorafenib (10 mg/kg) and SR9009 (100 mg/kg; *n* = 15). **a**, **b** Tumor growth was monitored by ultrasound imaging at baseline and at the end of treatment. The combination therapy produced a more pronounced reduction in tumor volume compared with sorafenib monotherapy. **c** This enhanced therapeutic response is further illustrated in the waterfall plot, which highlights a greater proportion of tumor nodules showing regression in mice treated with the combination regimen compared with those receiving sorafenib alone.

To further evaluate the therapeutic efficacy of the sorafenib + SR9009, we tested the combination on primary tumors developed in TG221 transgenic mice. Mice received a single intraperitoneal injection of N-nitrosodiethylamine (DEN) ten days after birth, and liver tumors became detectable by 20–22 weeks of age. Tumor growth was monitored weekly by high-resolution ultrasound imaging. Following tumor detection, animals were randomized into two treatment groups (*n* = 15 per group): sorafenib (10 mg/kg) or sorafenib (10 mg/kg) combined with SR9009 (100 mg/kg), administered for 23 days. Based on previous studies [20], the dose of sorafenib was reduced to 10 mg/kg, since the standard 30 mg/kg dose displayed near-maximal single-agent activity, masking potential additive or synergistic effects of SR9009 (data not shown). Under this optimized regimen, the sorafenib + SR9009 combination showed markedly enhanced antitumor efficacy. As illustrated in Fig. 7a, b, tumor burden significantly regressed in the majority of treated mice after three weeks of combined therapy. The *waterfall plot* (Fig. 7c) further highlights a reduction in the size of most tumor nodules in the combination group, whereas tumors in sorafenib-only–treated mice continued to grow progressively. Collectively, these findings demonstrate that SR9009 potentiates the antitumor effects of sorafenib in primary HCC, even at reduced sorafenib doses, while simultaneously lowering the incidence of drug-related toxicity.

## Discussion

This study demonstrates that the REV-ERBα/β agonist SR9009 enhances the antitumor efficacy of sorafenib without increasing systemic toxicity in vivo. Synthetic REV-ERB agonists such as GSK4112, SR9009 and SR9011 [11] have attracted attention due to their ability to modulate metabolic and proliferative pathways linked to tumorigenesis [28, 29]. Previous studies have shown that SR9009 exerts cytotoxic and antiproliferative effects in various tumor cell lines and inhibits tumor growth in xenograft models by repressing autophagy and lipid metabolism [17, 18].

In our experimental models, SR9009 alone displayed cytotoxic effects only at relatively high concentrations, whereas its combination with sorafenib produced a strong synergistic antitumor activity. The combined treatment markedly reduced cell viability, clonogenic potential, and tumor growth, both in vitro and in vivo. Mechanistically, this synergism appears to result from the complete suppression of mitochondrial oxidative phosphorylation (OxPhos), leading to a collapse of mitochondrial bioenergetics.

Transcriptomic analyses of sorafenib-resistant cells revealed a significant enrichment of OxPhos-related genes, suggesting that enhanced mitochondrial activity may serve as an adaptive mechanism to counteract sorafenib-induced metabolic stress. Sorafenib is known not only to inhibit RAF/MEK/ERK and angiogenic signaling pathways by inhibiting VEGFR and PDGFR signaling [30], but also to interfere with mitochondrial respiration [21, 31, 32]. Therefore, the upregulation of OxPhos genes likely reflects a compensatory response to maintain energy production and survival. SR9009, which has been reported to suppress mitochondrial respiration, acts as a metabolic antagonist of this adaptation. Consistent with this interpretation, our data show that the combination of sorafenib and SR9009 nearly abolished mitochondrial respiration and ATP production, accompanied by a strong decrease in mitochondrial complex proteins such as SDHA (Succinate Dehydrogenase subunit A), COX1 (Cytochrome c oxidase I) and Cytochrome c.

Importantly, this profound inhibition of mitochondrial metabolism was selectively cytotoxic to hepatoma cells, which rely heavily on mitochondrial ATP production, while sparing normal hepatocytes and fibroblasts [33]. In mouse models, the combination treatment reduced tumor burden without increasing hepatotoxicity, aligning with previous evidence that SR9009 can exert hepatoprotective and anti-inflammatory effects, reducing steatosis and cellular stress in non-tumor hepatic tissue [23–25].

Mechanistically, SR9009 may compete with heme for binding to REV-ERBα/β [15], thereby displacing heme and increasing intracellular free heme levels. Although originally developed as a REV-ERBα/β agonist, SR9009 can potentially interfere with other heme-dependent enzymes, including mitochondrial cytochromes [34]. Given the high reactivity of unbound heme, this displacement may promote mitochondrial dysfunction and enhance cytotoxicity. Supporting this hypothesis, we observed that *Hmox1*, encoding heme oxygenase-1 (HO-1), the enzyme responsible for degrading free heme, was strongly upregulated in sorafenib-resistant cells. HO-1 is a well-established cytoprotective enzyme that converts heme into biliverdin, bilirubin, and ferrous iron (Fe²⁺) [35]. The concomitant upregulation of ferritin in resistant cells further suggests that detoxification of free heme and sequestration of reactive iron are integral components of the adaptive resistance mechanism. Indeed, HO-1 functions as a key antioxidant and cytoprotective enzyme in liver disease [36], with growing evidence linking its upregulation to chemoresistance [37]. A similar protective role against heme-induced cytotoxicity was reported in breast cancer [38]. Functional validation through *Hmox1* knockdown confirmed the protective role of HO-1, as silencing of this gene decreased cell viability and mitochondrial activity in resistant cells. This indicates that HO-1 upregulation contributes to the survival advantage of sorafenib-resistant HCC cells.

Moreover, the SR9009-induced accumulation of free heme and reactive oxygen species (ROS) likely drives the observed synergistic cytotoxicity in combination with sorafenib. The elevated HO-1 protein levels detected after combined treatment reflect a strong cellular stress response, consistent with oxidative damage and lipid peroxidation triggered by excessive ROS generation.

In vivo, the combination of SR9009 and sorafenib showed superior efficacy compared with sorafenib alone, both in primary transgenic liver tumors and in xenografts derived from sorafenib-resistant cells, without exacerbating toxicity. Previous studies have similarly reported that SR9009 is well tolerated and even protective in cardiovascular [39] and metabolic disease models, including reducing atherosclerotic plaque formation and attenuating cardiac hypertrophy, further supporting its safety profile [40].

Collectively, our findings reveal that the metabolic adaptations conferring resistance to sorafenib, particularly the upregulation of mitochondrial respiration and heme detoxification pathways, can be therapeutically exploited. Pharmacological activation of REV-ERBs with SR9009 disrupts these adaptive mechanisms by impairing OxPhos and altering heme metabolism, leading to irreversible mitochondrial dysfunction and cell death, without worsening its tolerability.

Overall, this study highlights a rational strategy to enhance sorafenib efficacy by exploiting metabolic vulnerabilities in hepatocellular carcinoma. Integrating oncogene-targeted therapy with metabolic modulation offers a means to overcome adaptive resistance while minimizing systemic toxicity. Beyond sorafenib, this combinatorial approach provides a conceptual framework for improving therapeutic efficacy and safety by coupling oncogene-targeted agents with compounds that disrupt tumor-specific metabolic dependencies.

## Materials and methods

### Cell lines

Murine Hep55.1C (440201) and Hep56.1D (440204) cells, obtained from hepatomas of C57BL/6J mice, were obtained from CLS Cell Lines Service GmbH, Germany. The two cell lines differed in the status of the Tp53 gene: wild type in Hep55.1C and mutated (pCys132Trp) in Hep56.1D. Hep55.1C sorafenib-resistant cells (H55-RES) were obtained by in vivo selection from tumor xenografts of C57BL/6J mice as previously described [20]. Briefly, to obtain H55-RES, cells, C57BL/6 mice bearing H55.1C cells derived xenograft were treated with sorafenib. After a month of sensitivity to the drug, some tumors start to regrew. To confirm stable sorafenib resistance, resistant xenograft-derived cells were re-implanted into new mice, and notably no significant difference in tumor growth was observed between sorafenib-treated and untreated groups. We developed and analyzed 5 different resistant cell lines (named H55-RES 191, 412, 422, 423, 424). For in vitro and in vivo experiments, the H55-RES 191 cell line was used. Normal baby mouse kidney (BMK) fibroblasts were established from the normal kidneys of newborn C57BL/6J mice as previously described [41]. The human HCC cell lines Hep3B (HB-8064), HepG2 (HB-8065) and SNU449 (CRL-2234) were obtained from the American Type Culture Collection (ATCC, Manassas, VA, USA), and the Huh7 cell line (300156) was obtained from CLS Cell Lines Service GmbH. All cell lines were propagated and maintained in Iscove’s modified Dulbecco’s medium supplemented with 10% fetal bovine serum (FBS), 0.1% gentamicin, and 1% L-glutamine (Sigma‒Aldrich, St. Louis, MO, USA). All cell lines were authenticated by the supplier and tested for mycoplasma contamination (MycoAlert Mycoplasma Detection kit, LT07-418, Lonza Group Ltd., Basel Switzerland).

### Drugs

For in vitro experiments, Sorafenib (S-8599, LC Laboratories, Woburn, MA, USA), SR9009 (1379686-30-2, BOC SCIENCES, NY, USA) and IACS-010759 (HY-112037, MedChemExpress, NJ 08852, USA) were solubilized in dimethyl sulfoxide (DMSO). For all in vivo experiments, sorafenib (10 mg/kg or 30 mg/kg) was dissolved in a 50:50 solution of Cremophor EL/ ethanol and was administered daily by gavage, while SR9009 (100 mg/kg) was dissolved in 15% Cremophor EL solution and was administered daily by intraperitoneal injection (i.p).

### Transfection

Dicer-substrate siRNAs (DsiRNAs) are chemically synthesized 27mer RNA duplexes processed by Dicer, characterized by an increased potency in RNA interference compared to traditional, 21mer siRNAs [42]. A TriFECTa kit (IDT, Integrated DNA Technologies) containing three specific pre-designed DsiRNAs targeting murine *Hmox1* gene was used. Specifically, the duplex oligonucleotides sequences were as follows: DsiRNA mm.Ri.Hmox1.13.1: Duplex Oligo sense –rUrArArArUrGrGrCrArUrUrArUrCrUrArArCrArGrUrCrACT; Duplex Oligo antisense – rArGrUrGrArCrUrGrUrUrArGrArUrArArUrGrCrCrArUrUrUrArUrU; DsiRNA mm.Ri.Hmox1.13.2: Duplex Oligo sense – rArUrGrGrCrUrUrCrCrUrUrGrUrArCrCrArUrArUrCrUrACA; Duplex Oligo antisense – rUrGrUrArGrArUrArUrGrGrUrArCrArArGrGrArArGrCrCrArUrCrA; DsiRNA mm.Ri.Hmox1.13.3: Duplex Oligo sense – rGrGrGrArArUrUrUrArUrGrCrCrArUrGrUrArArArUrGrCAA; Duplex Oligo antisense – rUrUrGrCrArUrUrUrArCrArUrGrGrCrArUrArArArUrUrCrCrCrArC. DsiRNAs transfection was carried out by using Lipofectamine 2000 (#11668027, Invitrogen, Thermo Fisher Scientific, Carlsbad, CA, USA), in accordance with the manufacturer’s indications.

### Cell viability assays

The Muse® Count & Viability Kit was used to perform quantitative analysis of cell counts and viability (MCH100102, Luminex Corporation, Austin, TX, USA). All assays were performed in triplicate, and the data were analyzed with a Muse® Cell Analyzer instrument (Merck Millipore). The PrestoBlue™ HS Cell Viability Reagent (#P50200; Invitrogen, Thermo Fisher Scientific, Carlsbad, CA) was used to measure the half maximal inhibitory concentration (IC50) to evaluate the efficacy of the drug in vitro. All assays were performed in triplicate and analyzed with a Tecan Infinite 200 Pro M Plex Microplate Reader (Tecan Austria Gmbh).

### Mitochondrial function assays and Lactate Assay

The Agilent Seahorse Cell Mito Stress Test (103015-100, Agilent Technologies, Wilmington, DE, USA) was used to evaluate mitochondrial function by measuring the oxygen consumption rate (OCR) of the cells, while the Agilent Seahorse XF Glycolysis Stress Test (103020-100, Agilent Technologies) was used to evaluate glycolytic function by measuring the extracellular acidification rate (EACR) of the cells. Real-time measurements of the OCR and ECAR were performed on Seahorse XFe/XF extracellular flux analyzers. The Mitochondrial ToxGlo™ assay (G8000, Promega, Madison, WI, USA) was used to evaluate mitochondrial function through differential measurement of biomarkers associated with changes in cell membrane integrity and cellular ATP levels. The Lactate-Glo™ Assay (#J5021, Promega, Madison, WI, USA), a bioluminescent detection system that couples lactate oxidation and NADH production, was used to evaluate lactate levels in cell culture media of treated cells and normalized on cell viability, measured with the resazurin-based PrestoBlue HS reagent (#P50200; Invitrogen, Thermo Fisher), in accordance with the manufacturer’s instructions. Assays were performed in triplicate and analyzed in a Tecan Infinite 200 Pro M Plex microplate reader (Tecan Austria Gmbh).

### Colony formation assay

1 ml of 0.8% Sea Plaque Low Melt Agarose (#50101, Lonza Group Ltd., Basel Switzerland) was distribute in 6-well plates to prepare base agarose layer. A single-cell suspension from Hep55.1C cells, containing 5 × 10^3^ cells/well was prepared and mixed with an upper agarose layer (0.48%). 1 ml of cell/agar mixture was immediately overlaied in triplicate for each treatment condition in wells containing the solidified base layer. Each plate was cooled for ~5 min at 4 °C to solidify agarose and then incubated at 37 °C in a humidified atmosphere with 5% CO_2_ for 3 weeks. Colonies formation was monitored during the incubation time and representative images were captured with a DS-L3 Camera (#M555E, Nikon). Colonies were finally stained adding 1 ml of PBS containing 4% formaldehyde and 0.005% crystal violet to each well.

### Western blot and antibodies

Cell cultures were harvested and lysed with radioimmune precipitation buffer (RIPA) (#R0278; Sigma‒Aldrich) as previously described [20]. Protein extracts (10 μg from each of the samples) were subjected to SDS‒PAGE (4–15% Tris Glicyne Gel, no. 4561083, Bio-Rad) and then transferred to a PVDF membrane (no. 1704156, Bio-Rad). After incubation with 5% blocking agent, the membranes were incubated overnight at 4°C with the following rabbit antibodies: SDHA (D6J9M)XP, #11998), Cytocrome c (D18C7, #11940), COX1/MT-CO1 (E2I2R, #55159), HO-1 (E3F4S, #43966), PARP (#9542, and Cleaved PARP (#9544), all from Cell Signaling Technology, Danvers, MA, USA. A monoclonal anti-glyceraldehyde-3-phosphate dehydrogenase (GAPDH) antibody (clone 2D9, TA802519; OriGene Technologies, Rockville, MD, USA) was used as a loading control. For chemiluminescent detection, a horseradish peroxidase-conjugated secondary antibody (#7074; Cell Signaling Technology) was used in combination with Clarity Western ECL Blotting Substrate (#170-5060; Bio-Rad), and digital images were acquired using Chemidoc (Bio-Rad). Signals were quantified with ImageJ software (https://imagej.nih.gov), and protein expression levels were normalized to GAPDH protein expression.

### RNA purification

Total RNA was extracted from frozen liver cells or tissues using a Maxwell RSC instrument (Promega Italia, Milan) and a Maxwell RSC miRNA from a tissue purification kit (#AS1460, Promega) according to the manufacturer’s instructions.

### Free heme, lipid peroxidation (MDA) and intracellular ROS measurement

Measurement of intracellular free heme in cellular lysates was based on the interaction of an inactive apo-horseradish peroxidase (apoHRP, #293A0000, Calzyme Labs, San Luis Obispo, CA, USA) with free heme to form an active holoHRP, as previously described [43]. 10 µg of cellular lysate collected after 8 h of treatment were analyzed in presence of apo-HRP enzyme (5 µM). The reconstitution time of active holo-HRP activity with free heme was established at 10 min. The activity of the reconstituted HRP was measured by colorimetric oxidation of the Peroxidase substrate 3,3’,5,5’-tetramethylbenzidine (TMB, #T4444; Sigma‒Aldrich) and was converted to free heme concentration using a standard curve established with increasing concentrations of hemin (0–2,5 nM) (#51280; Sigma‒Aldrich). Oxidatve stress was determined by a colorimetric quantification of malondialdehyde (MDA) levels in cell lysates using the Lipid Peroxidation (MDA) Assay kit (#MAK568; Sigma‒Aldrich), while intracellular ROS was determined in living cells by fluorimetric assay (Fluorimetric Intracellular ROS kit, #MAK143; Sigma‒Aldrich) and normalized on cell viability measured with the resazurin-based PrestoBlue HS reagent (#P50200; Invitrogen, Thermo Fisher), in accordance with the manufacturer’s instructions. All the assays were performed in triplicate and analyzed in a Tecan Infinite 200 Pro M Plex microplate reader (Tecan Austria Gmbh).

### RNA sequencing

RNA quality and integrity were checked by a Bioanalyzer 2100 and Agilent RNA 6000 Nano Kit (No. 5067-1511 Agilent, Santa Clara, CA, USA). RNA-seq libraries were prepared using the Qiagen QIAseq Fast Select rRNA HMR kit (no. 334386, Qiagen Düsseldorf, Germany) for ribosomal RNA depletion and the Qiagen QIAseq stranded total RNA library kit (no. 180745, Qiagen) according to the manufacturer’s instructions. RNA sequencing was performed according to the Illumina pipeline on a NextSeq 500 instrument (Illumina, San Diego, CA, USA.) using the NextSeq 500/550 High Output Kit v2.5 150 Cycles (#20024907, Illumina).

### Bioinformatics analysis

The obtained sequences were mapped to the mouse genome (GRCm38) using the HISAT2 algorithm [44] and a prebuilt genome index downloadable from the HISAT2 website. Then, StringTie [45] was used to assemble and quantify the transcripts in each sample. Finally, the expressed transcripts were normalized using the “DESeq2” package [46] for R. The R package “TCseq” v.1.22.6 [http://www.bioconductor.org/packages/release/bioc/html/TCseq.html] was used to characterize gene expression patterns across sample groups. Functional enrichment analysis of the selected genes was performed using the R package “clusterProfiler” v. 4.6.2 [47] with the Kyoto Encyclopedia of Genes and Genomes (KEGG) and Gene Ontology (GO) databases. Heatmaps were generated with the R package “pheatmap” [https://CRAN.R-project.org/package=pheatmap]. Gene Set Enrichment Analysis (GSEA) [48] was performed using GSEA 4.1.0 with a database containing Hallmarks MSigDB collection signature gene sets and pathway gene sets canons derived from the Reactome Path Database. Significant gene sets with an FDR-adjusted *p* value < 0.05 were selected. Differential expression of genes belonging to the HALLMARK_OXIDATIVE_PHOSPHORYLATION gene set was assessed by calculating the fold change, while statistical significance was determined using the Benjamini–Hochberg method for adjusted *p*-value correction.

### In vivo studies on mice

The study was conducted according to the Guidelines for the Care and Use of Laboratory Animals of the Italian Ministry of Health. All studies involving animal experiments complied with the Animal Research: Reporting of In Vivo Experiments (ARRIVE) guidelines. To comply with Directive 2010/63/EU of the European Parliament and of the Council, which requires the lowest number of experimental animals, G*Power (http://www.gpower.hhu.de/) was used to define the minimum sample size for the experiments. All animals were randomly assigned to different treatment groups at the start of the studies. No blinding was performed. The protocols for animal testing were approved by the Italian Ministry of Health (approval no. 701/2017-PR, no. 645/2021-PR and n. 337/2025-PR). All mice were maintained in ventilated cabinets at 25 °C on a 12-h light-dark cycle, with food and water available ad libitum. For xenotransplantation experiments, wild-type C57BL/6J female mice (6–8 weeks) were obtained from Charles River Laboratories s.r.l. (Calco, IT). To test the therapeutic effect of SR9009 in combination with sorafenib, 5 × 10^4^ Hep55.1C wild type cells and 5 × 10^4^ H55-RES cells were implanted subcutaneously respectively into the left or right side of female C57BL/6 mice as previously described [20]. When the tumors reached a volume of ~50 mm^3^ (10 days after cell injection), the mice were randomly selected for treatment. To test the therapeutic effect of SR9009 in combination with sorafenib in primary liver tumors, TG221 transgenic mice [49] were used. Ten-day-old neonatal TG221 male mice received an i.p. injection of N-Nitrosodiethylamine (DEN) (#N0756, Sigma‒Aldrich) (7.5 mg/kg body weight) to facilitate tumor development. Mice were monitored for the presence of liver lesions using a diagnostic ultrasound device (Philips IU22) as previously described [20]. Mice were randomly assigned to treatment groups at six months of age. At the end of the experiments, the mice were sacrificed and the tumor tissues were collected, immediately frozen in liquid nitrogen and stored at −80 °C.

### In vivo toxicity assessment

Blood samples were collected via a retro-orbital method from mice anesthetized with a mixture of isoflurane [2-chloro-2-(difluoromethoxy)-1,1,1-trifluoroethane] and oxygen. The alanine aminotransferase (ALT) activity test (MAK052, Sigma‒Aldrich) was used to evaluate liver function in the serum of mice according to the manufacturer’s instructions. Serum was obtained by centrifugation at 1000 × *g*/RT for 10 min and stored at −80 °C. All tests were performed in triplicate, and the data were analyzed with a Tecan Infinite 200 Pro M Plex microplate reader (Tecan Austria Gmbh). Red blood cell (RBC) and white blood cell (WBC) counts were evaluated using a Neubauer Counting Chamber (BRAND™ BLAUBRAND™, 10195580), and the number of cells per µl was calculated using the following formula: number of cells/counted area (mm^2^) × chamber depth (mm) × dilution factor. The reference values for the hematological and biochemical parameters of C57BL/6NCrl mice can be found on the Charles River website (Biochemistry and Hematology for C57BL/6NCrl Mouse Colonies in North America).

### Statistical analysis

To evaluate the statistical significance of group similarity, we used the t test when the variance of the two compared samples was equal or the Welch *t* test when two samples showed unequal variances. A *p* value ≤ 0.05 was considered to indicate statistical significance. Variances between groups were assessed using the *F* test. When appropriate, the data are expressed as the mean ± standard deviation (SD). GraphPad Prism 6.0 (GraphPad Software, La Jolla, CA, USA) was used for data analysis. The synergy between two drug pairs was determined using the SynergyFinder version 3.0 package, and the highest single agent (HSA) model was used. With a synergy score less than −10, the interaction between two drugs is likely to be antagonistic; from −10 to 10, it is likely that the interaction between two drugs is additive; and greater than 10, the interaction between two drugs is likely to be synergistic. A waterfall plot was used to evaluate the response of mice to drug treatment based on tumor burden. The percentage change in tumor volume was calculated using the formula [(Xf-Xi)/Xi]*100, where Xi represents the volume of the tumor nodule detected by ultrasound analysis on day 0, while Xf represents the volume of the tumor nodule detected after 21 days of treatment with the therapeutic agent. The waterfall plot was produced using the Phyton programming language with the help of the MATLABPoint library.

## Supplementary information

Supplementary Material

Supplementary Table 1

Supplementary Table 2

Full length Western Blot

## Data Availability

The authors declare that all the data supporting the findings of this study are available within the article and its Supplementary Information files. The RNA-seq raw data generated and analyzed during the current study are available in the NCBI Gene Expression Omnibus (GEO) data repository with the accession number GSE312483.

## References

[1] Bray F, Laversanne M, Sung H, Ferlay J, Siegel RL, Soerjomataram I, et al. Global cancer statistics 2022: GLOBOCAN estimates of incidence and mortality worldwide for 36 cancers in 185 countries. CA Cancer J Clin. 2024;74:229–63. 10.3322/caac.21834 38572751

[2] Llovet JM, Ricci S, Mazzaferro V, Hilgard P, Gane E, Blanc JF, et al. Sorafenib in advanced hepatocellular carcinoma. N Engl J Med. 2008;359:378–90. 18650514 10.1056/NEJMoa0708857

[3] Kudo M, Finn RS, Qin S, Han KH, Ikeda K, Piscaglia F, et al. Lenvatinib versus sorafenib in first-line treatment of patients with unresectable hepatocellular carcinoma: a randomised phase 3 non-inferiority trial. Lancet. 2018;391:1163–73. 29433850 10.1016/S0140-6736(18)30207-1

[4] Finn RS, Qin S, Ikeda M, Galle PR, Ducreux M, Kim TY, et al. Atezolizumab plus Bevacizumab in Unresectable Hepatocellular Carcinoma. N Engl J Med. 2020;382:1894–905. 32402160 10.1056/NEJMoa1915745

[5] Yang X, Lin Wang D, Yang J, Zhao X. H. Atezolizumab plus bevacizumab for unresectable hepatocellular carcinoma. Lancet Oncol. 2020;21:e412. 32888462 10.1016/S1470-2045(20)30430-7

[6] Vogel A, Martinelli E. clinicalguidelines@esmo.org EGCEa, Committee EG. Updated treatment recommendations for hepatocellular carcinoma (HCC) from the ESMO Clinical Practice Guidelines. Ann Oncol. 2021;32:801–5. 33716105 10.1016/j.annonc.2021.02.014

[7] Chan LL, Kwong TT, Yau JCW, Chan SL. Treatment for hepatocellular carcinoma after immunotherapy. Ann Hepatol. 2025;30:101781. 39929474 10.1016/j.aohep.2025.101781

[8] Cappuyns S, Corbett V, Yarchoan M, Finn RS, Llovet JM. Critical appraisal of guideline recommendations on systemic therapies for advanced hepatocellular carcinoma: a review. JAMA Oncol. 2024;10:395–404. 37535375 10.1001/jamaoncol.2023.2677 PMC10837331

[9] Singal AG, Llovet JM, Yarchoan M, Mehta N, Heimbach JK, Dawson LA, et al. AASLD practice Guidance on prevention, diagnosis, and treatment of hepatocellular carcinoma. Hepatology. 2023;78:1922–65. 37199193 10.1097/HEP.0000000000000466 PMC10663390

[10] Jiang X, Ge X, Huang Y, Xie F, Chen C, Wang Z, et al. Drug resistance in TKI therapy for hepatocellular carcinoma: mechanisms and strategies. Cancer Lett. 2025;613:217472. 39832650 10.1016/j.canlet.2025.217472

[11] Noel R, Song X, Shin Y, Banerjee S, Kojetin D, Lin L, et al. Synthesis and SAR of tetrahydroisoquinolines as Rev-erbalpha agonists. Bioorg Med Chem Lett. 2012;22:3739–42. 22560469 10.1016/j.bmcl.2012.04.023 PMC3488456

[12] Burke L, Downes M, Carozzi A, Giguere V, Muscat GE. Transcriptional repression by the orphan steroid receptor RVR/Rev-erb beta is dependent on the signature motif and helix 5 in the E region: functional evidence for a biological role of RVR in myogenesis. Nucleic Acids Res. 1996;24:3481–9. 8836172 10.1093/nar/24.18.3481 PMC146133

[13] Lazar MA, Hodin RA, Cardona G, Chin WW. Gene expression from the c-erbA alpha/Rev-ErbA alpha genomic locus. Potential regulation of alternative splicing by opposite strand transcription. J Biol Chem. 1990;265:12859–63. 2165488

[14] Gomatou G, Karachaliou A, Veloudiou OZ, Karvela A, Syrigos N, Kotteas E. The role of REV-ERB receptors in cancer pathogenesis. Int. J Mol Sci. 2023;24:8980. 10.3390/ijms24108980 PMC10219396 37240325

[15] Raghuram S, Stayrook KR, Huang P, Rogers PM, Nosie AK, McClure DB, et al. Identification of heme as the ligand for the orphan nuclear receptors REV-ERBalpha and REV-ERBbeta. Nat Struct Mol Biol. 2007;14:1207–13. 18037887 10.1038/nsmb1344 PMC2743565

[16] Yin L, Wu N, Curtin JC, Qatanani M, Szwergold NR, Reid RA, et al. Rev-erbalpha, a heme sensor that coordinates metabolic and circadian pathways. Science. 2007;318:1786–9. 18006707 10.1126/science.1150179

[17] Shen W, Zhang W, Ye W, Wang H, Zhang Q, Shen J, et al. SR9009 induces a REV-ERB dependent anti-small-cell lung cancer effect through inhibition of autophagy. Theranostics. 2020;10:4466–80. 32292508 10.7150/thno.42478 PMC7150483

[18] Sulli G, Rommel A, Wang X, Kolar MJ, Puca F, Saghatelian A, et al. Pharmacological activation of REV-ERBs is lethal in cancer and oncogene-induced senescence. Nature. 2018;553:351–5. 29320480 10.1038/nature25170 PMC5924733

[19] Dierickx P, Emmett MJ, Jiang C, Uehara K, Liu M, Adlanmerini M, et al. SR9009 has REV-ERB-independent effects on cell proliferation and metabolism. Proc Natl Acad Sci USA. 2019;116:12147–52. 31127047 10.1073/pnas.1904226116 PMC6589768

[20] Callegari E, Guerriero P, Bassi C, D’Abundo L, Frassoldati A, Simoni E, et al. miR-199a-3p increases the anti-tumor activity of palbociclib in liver cancer models. Mol Ther Nucleic acids. 2022;29:538–49. 36035756 10.1016/j.omtn.2022.07.015 PMC9395755

[21] Li Y, Xia J, Shao F, Zhou Y, Yu J, Wu H, et al. Sorafenib induces mitochondrial dysfunction and exhibits synergistic effect with cysteine depletion by promoting HCC cells ferroptosis. Biochem Biophys Res Commun. 2021;534:877–84. 33162029 10.1016/j.bbrc.2020.10.083

[22] Everett LJ, Lazar MA. Nuclear receptor Rev-erbalpha: up, down, and all around. Trends Endocrinol Metab: TEM. 2014;25:586–92. 25066191 10.1016/j.tem.2014.06.011 PMC4252361

[23] Ni Y, Nan S, Zheng L, Zhang L, Zhao Y, Fu Z. Time-dependent effect of REV-ERBalpha agonist SR9009 on nonalcoholic steatohepatitis and gut microbiota in mice. Chronobiol Int. 2023;40:769–82. 37161366 10.1080/07420528.2023.2207649

[24] Fekry B, Ribas-Latre A, Drunen RV, Santos RB, Shivshankar S, Dai Y, et al. Hepatic circadian and differentiation factors control liver susceptibility for fatty liver disease and tumorigenesis. FASEB J. 2022;36:e22482. 35947136 10.1096/fj.202101398R PMC10062014

[25] Wang J, Cui J, Hao T, Zhang Q, Chen Y, Guo L, et al. Regulation of cyclophosphamide induced hepatotoxicity by REV-ERBalpha modifiers. Expert Opin Drug Metab Toxicol. 2025;21:885–95. 40211567 10.1080/17425255.2025.2490741

[26] Zhang J, Zhang Q. Using seahorse machine to measure OCR and ECAR in cancer cells. Methods Mol Biol. 2019;1928:353–63. 30725464 10.1007/978-1-4939-9027-6_18

[27] Pujalte-Martin M, Belaid A, Bost S, Kahi M, Peraldi P, Rouleau M, et al. Targeting cancer and immune cell metabolism with the complex I inhibitors metformin and IACS-010759. Mol Oncol. 2024;18:1719–38. 38214418 10.1002/1878-0261.13583 PMC11223609

[28] Ercolani L, Ferrari A, De Mei C, Parodi C, Wade M, Grimaldi B. Circadian clock: time for novel anticancer strategies?. Pharmacol Res. 2015;100:288–95. 26319800 10.1016/j.phrs.2015.08.008

[29] Battaglin F, Chan P, Pan Y, Soni S, Qu M, Spiller ER, et al. Clocking cancer: the circadian clock as a target in cancer therapy. Oncogene. 2021;40:3187–3200. 33846572 10.1038/s41388-021-01778-6 PMC8549632

[30] Coventon J. A review of the mechanism of action and clinical applications of sorafenib in advanced osteosarcoma. J Bone Oncol. 2017;8:4–7. 28828294 10.1016/j.jbo.2017.07.001 PMC5552021

[31] Bull VH, Rajalingam K, Thiede B. Sorafenib-induced mitochondrial complex I inactivation and cell death in human neuroblastoma cells. J Proteome Res. 2012;11:1609–20. 22268697 10.1021/pr200790e

[32] Zhang C, Liu Z, Bunker E, Ramirez A, Lee S, Peng Y, et al. Sorafenib targets the mitochondrial electron transport chain complexes and ATP synthase to activate the PINK1-Parkin pathway and modulate cellular drug response. J Biol Chem. 2017;292:15105–20. 28673964 10.1074/jbc.M117.783175 PMC5592685

[33] Di Gregorio J, Petricca S, Iorio R, Toniato E, Flati V. Mitochondrial and metabolic alterations in cancer cells. Eur J Cell Biol. 2022;101:151225. 35453093 10.1016/j.ejcb.2022.151225

[34] Santucci R, Sinibaldi F, Cozza P, Polticelli F, Fiorucci L. Cytochrome c: an extreme multifunctional protein with a key role in cell fate. Int J Biol Macromol. 2019;136:1237–46. 31252007 10.1016/j.ijbiomac.2019.06.180

[35] Chiang SK, Chen SE, Chang LC. The role of HO-1 and its crosstalk with oxidative stress in cancer cell survival. Cells. 2021;10:2401. 10.3390/cells10092401 PMC8471703 34572050

[36] Ryter SW. Heme oxygenase-1: an anti-inflammatory effector in cardiovascular, lung, and related metabolic disorders. Antioxidants. 2022;11:555. 10.3390/antiox11030555 PMC8944973 35326205

[37] Wang H, Cheng Q, Bao L, Li M, Chang K, Yi X. Cytoprotective role of heme oxygenase-1 in cancer chemoresistance: focus on antioxidant, antiapoptotic, and pro-autophagy properties. Antioxidants. 2023;12:1217. 10.3390/antiox12061217 PMC10295073 37371947

[38] Wagener FA, Dankers AC, van Summeren F, Scharstuhl A, van den Heuvel JJ, Koenderink JB, et al. Heme Oxygenase-1 and breast cancer resistance protein protect against heme-induced toxicity. Curr Pharm Des. 2013;19:2698–707. 23092328 10.2174/1381612811319150004

[39] Li H, Song S, Tien CL, Qi L, Graves A, Nasiotis E, et al. SR9009 improves heart function after pressure overload independent of cardiac REV-ERB. Front Cardiovasc Med. 2022;9:952114. 35911512 10.3389/fcvm.2022.952114 PMC9329699

[40] Sitaula S, Billon C, Kamenecka TM, Solt LA, Burris TP. Suppression of atherosclerosis by synthetic REV-ERB agonist. Biochem Biophys Res Commun. 2015;460:566–71. 25800870 10.1016/j.bbrc.2015.03.070 PMC4855281

[41] Meneguzzi G, Chenciner N, Corallini A, Grossi MP, Barbanti-Brodano G, Milanesi G. The arrangement of integrated viral DNA is different in BK virus-transformed mouse and hamster cells. Virology. 1981;111:139–53. 6262997 10.1016/0042-6822(81)90660-7

[42] Kim DH, Behlke MA, Rose SD, Chang MS, Choi S, Rossi JJ. Synthetic dsRNA Dicer substrates enhance RNAi potency and efficacy. Nat Biotechnol. 2005;23:222–6. 15619617 10.1038/nbt1051

[43] Atamna H, Brahmbhatt M, Atamna W, Shanower GA, Dhahbi JM. ApoHRP-based assay to measure intracellular regulatory heme. Metallomics. 2015;7:309–21. 25525887 10.1039/c4mt00246f PMC4326600

[44] Kim D, Langmead B, Salzberg SL. HISAT: a fast spliced aligner with low memory requirements. Nat Methods. 2015;12:357–60. 25751142 10.1038/nmeth.3317 PMC4655817

[45] Pertea M, Pertea GM, Antonescu CM, Chang TC, Mendell JT, Salzberg SL. StringTie enables improved reconstruction of a transcriptome from RNA-seq reads. Nat Biotechnol. 2015;33:290–5. 25690850 10.1038/nbt.3122 PMC4643835

[46] Love MI, Huber W, Anders S. Moderated estimation of fold change and dispersion for RNA-seq data with DESeq2. Genome Biol. 2014;15:550. 25516281 10.1186/s13059-014-0550-8 PMC4302049

[47] Wu T, Hu E, Xu S, Chen M, Guo P, Dai Z, et al. clusterProfiler 4.0: A universal enrichment tool for interpreting omics data. Innovation. 2021;2:100141. 34557778 10.1016/j.xinn.2021.100141 PMC8454663

[48] Subramanian A, Tamayo P, Mootha VK, Mukherjee S, Ebert BL, Gillette MA, et al. Gene set enrichment analysis: a knowledge-based approach for interpreting genome-wide expression profiles. Proc Natl Acad Sci USA. 2005;102:15545–50. 16199517 10.1073/pnas.0506580102 PMC1239896

[49] Callegari E, Elamin BK, Giannone F, Milazzo M, Altavilla G, Fornari F, et al. Liver tumorigenicity promoted by microRNA-221 in a mouse transgenic model. Hepatology. 2012;56:1025–33. 10.1002/hep.25747 22473819

