# Supplementary material for: Targeting metabolic vulnerabilities: REV-ERB agonist SR9009 potentiates sorafenib efficacy in liver cancer
Source: Cell Death Discov. 2026 Jan 19;12:86. doi: 10.1038/s41420-026-02940-3 (PMC12877144; doi:10.1038/s41420-026-02940-3)
Supplement: Supplementary file 1 — Supplementary Material [file 41420_2026_2940_MOESM1_ESM.docx]

**Supplementary Material**

**Supplementary Tables**

**Supplementary Table 1. Normalized expression levels of genes belonging to the HALLMARK_OXIDATIVE_PHOSPHORYLATION gene set.**

**Supplementary Table 2.** Genes induced during early sorafenib exposure in Hep55.1C cells and further upregulated upon acquisition of sorafenib resistance.

**Supplementary Table 3.** Comparative Gene Set Enrichment Analysis (GSEA) of apoptosis-related pathways in Hep55.1C cells treated with the combination of sorafenib and SR9009 (SF+SR) versus sorafenib (SF) alone.

| **SR+SF vs SF enriched pathways** | **Normalized Enrichment Score** | **Adjusted pValue** |
| --- | --- | --- |
| positive regulation of mitochondrial outer membrane permeabilization involved in apoptotic signaling pathway | 1,864 | 2,51E-03 |
| negative regulation of toll-like receptor signaling pathway | 1,650 | 3,32E-02 |
| regulation of mitochondrial outer membrane permeabilization involved in apoptotic signaling pathway | 1,609 | 3,58E-02 |
| positive regulation of TRAIL-activated apoptotic signaling pathway | 1,480 | 6,82E-02 |
| activation of cysteine-type endopeptidase activity involved in apoptotic process | 1,346 | 1,02E-01 |
| podocyte apoptotic process | 1,393 | 1,35E-01 |
| regulation of mitochondrial membrane permeability involved in apoptotic process | 1,313 | 1,43E-01 |
| positive regulation of mitochondrial membrane permeability involved in apoptotic process | 1,316 | 1,46E-01 |
| regulation of TRAIL-activated apoptotic signaling pathway | 1,391 | 1,48E-01 |

**Supplementary Figures**

| **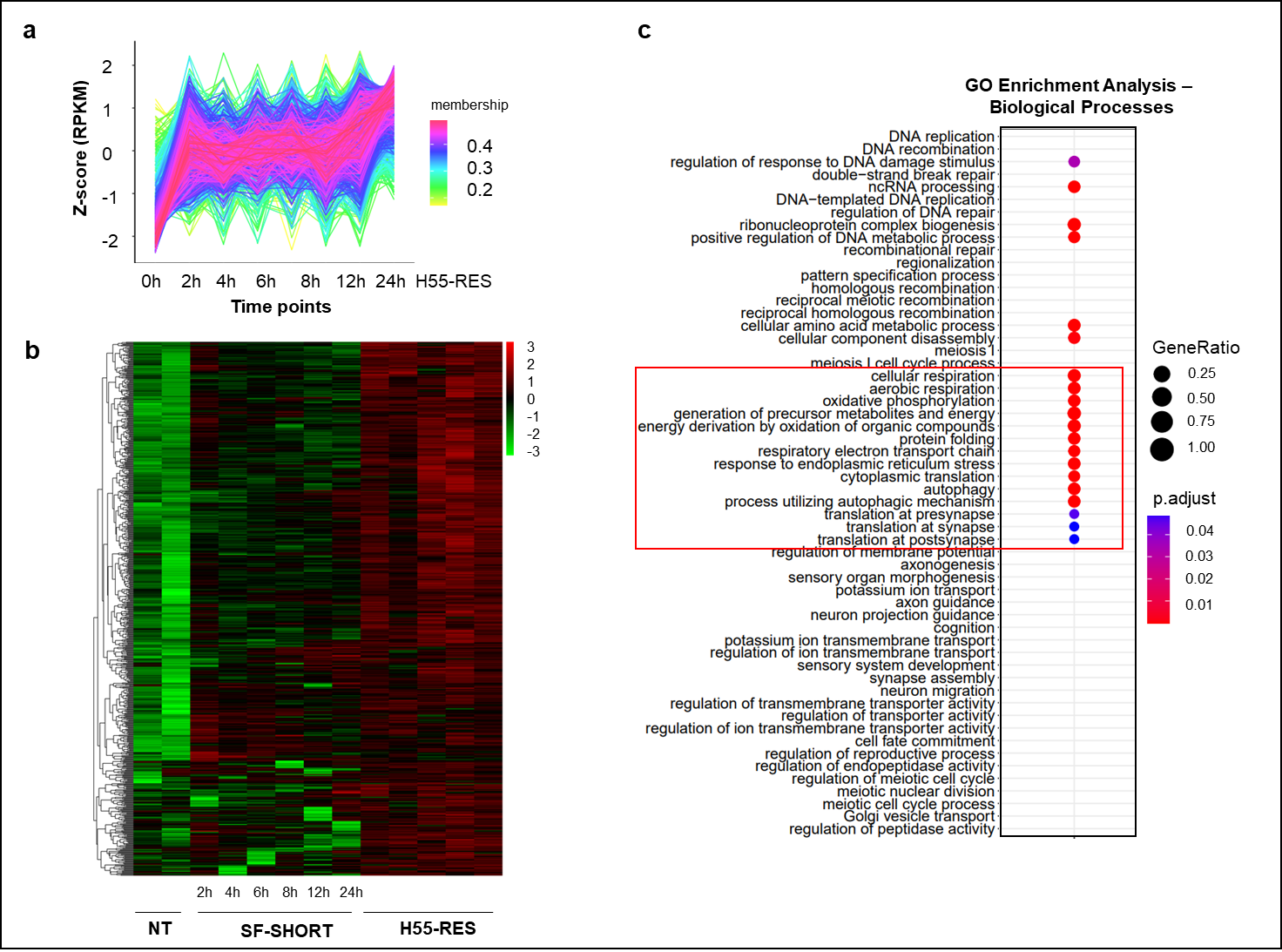** |
| --- |
| **Supplementary Fig. 1. Genes and biological processes up-regulated in Hep55.1C cells by sorafenib short-term treatment.** Time-course transcriptomic analysis of Hep55.1C cells exposed to sorafenib (0–24 h) revealed a progressive upregulation of a gene cluster (Supplementary Table 1) that was further increased in sorafenib-resistant cells (H55-RES). **(a)** TCseq-based analysis of RNA-seq data illustrating gene expression dynamics [<http://www.bioconductor.org/packages/release/bioc/html/TCseq.html>]. The Y-axis represents normalized gene expression values (log₂ scale), while the X-axis indicates treatment time points. **(b)** Heatmap representation of the same sorafenib-upregulated gene cluster, illustrating their expression dynamics across treatment conditions. Red denotes upregulation, and green denotes downregulation. **(c)** Gene Ontology (GO) enrichment analysis of biological processes associated with sorafenib-upregulated genes. Color intensity indicates the adjusted p-value (blue = higher, red = lower), with lower p-adjusted values representing more significantly enriched gene sets. Point size reflects the gene ratio within each GO term. NT = untreated cells; SF-SHORT = Hep55.1C cells treated with sorafenib at different time points (2 h, 4 h, 6 h, 8 h, 12 h, 24 h); H55-RES = cells resistant to 10 µM sorafenib and maintained in the presence of the drug. |

| **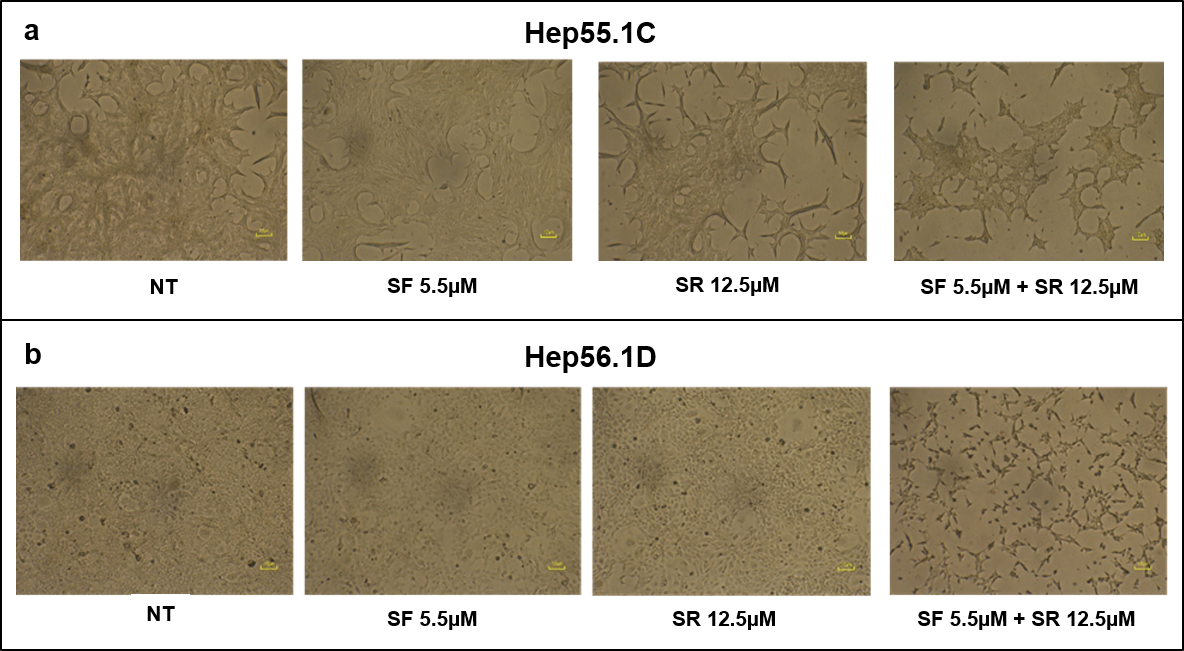** |
| --- |

**Supplementary Fig. 2. Cytotoxic effects of sorafenib, SR9009 and their combination in mouse hepatoma cell lines.** Representative phase-contrast images of Hep55.1C **(a**) and Hep56.1D **(b)** cells captured 48 hours after treatment demonstrate that the combined administration of sorafenib and SR9009 elicited the most pronounced cytotoxic response compared to either agent alone. Treatments were performed using 5.5 µM sorafenib and 12.5 µM SR9009, as described in Figure 1. (NT = untreated; SF = sorafenib; SR = SR9009; SF+SR = sorafenib + SR9009).

| 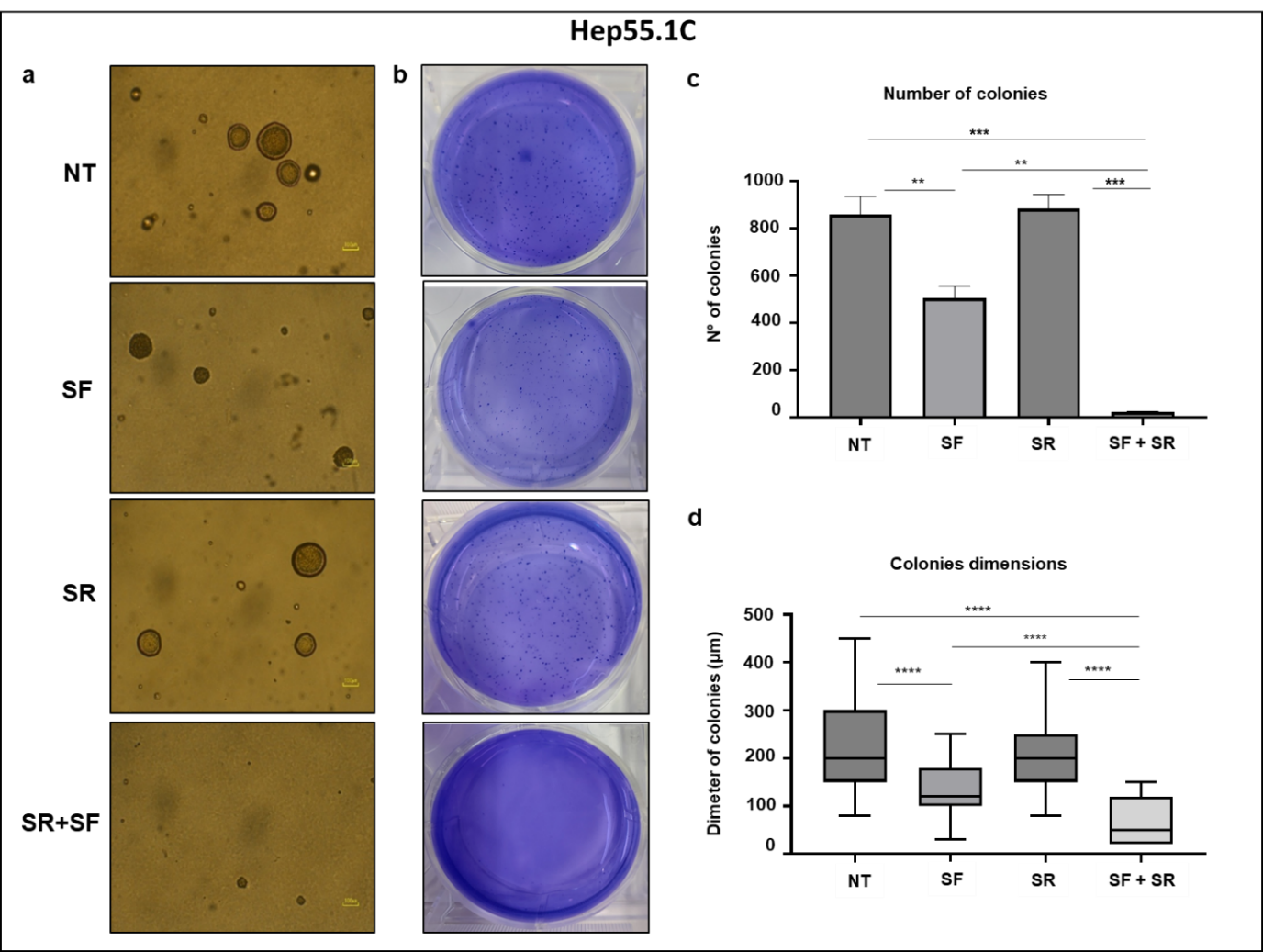 |
| --- |
| **Supplementary Fig. 3. Combined treatment with sorafenib and SR9009 impairs clonogenic growth of Hep55.1C** **cells. (a)** Representative images of Hep55.1C colonies grown in soft agar (**scale bar: 100 µm)** and **(b)** corresponding crystal violet-stained plates after two weeks of treatment with sorafenib (7.5 µM), SR9009 (12.5 µM) or their combination (SF+SR). Each condition was performed in triplicate. The combination treatment markedly reduced both the **number** **(c)** and diameter **size** **(d)** of colonies compared with untreated (NT) and single-agent groups, demonstrating a strong synergistic inhibition of cell proliferation and survival. To assess their number, only colonies ≥100 µm in diameter were considered. ****p value ≤ 0.01; ***p value ≤ 0.001; ****p value ≤ 0.0001.** |

| **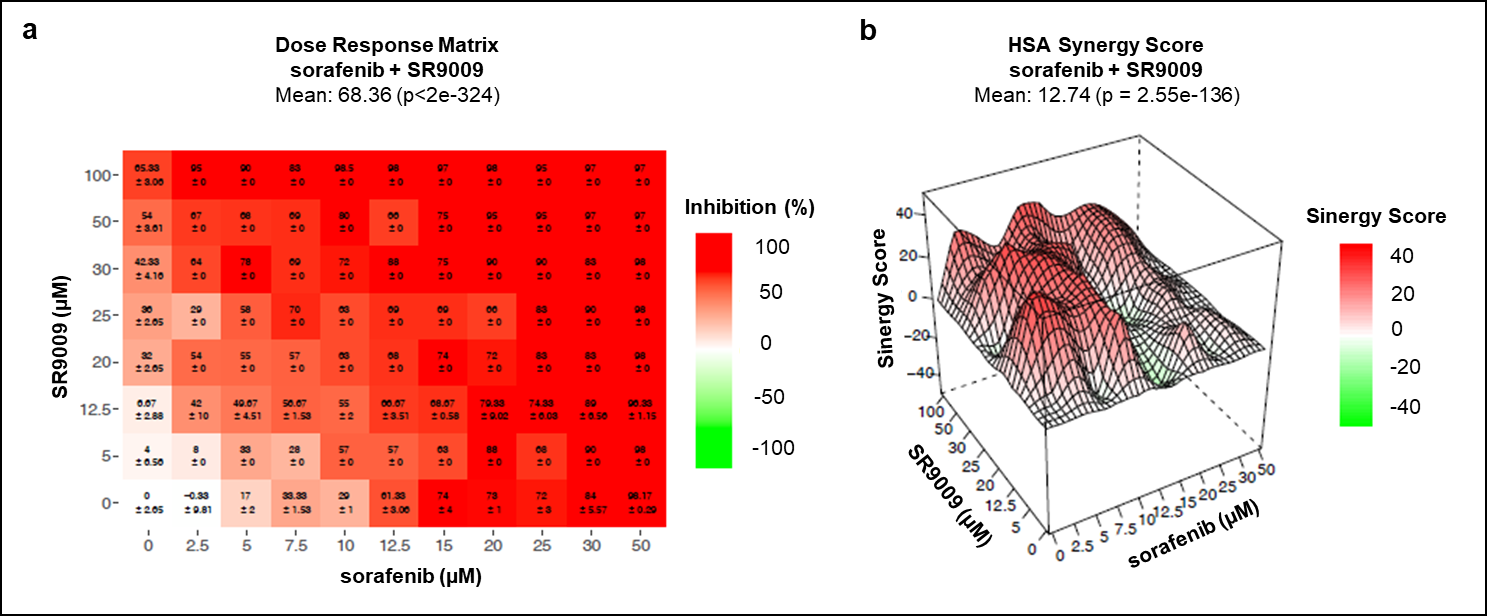** |
| --- |
| **Supplementary Fig. 4. Synergistic interaction between SR9009 and sorafenib in mouse Hep55.1C hepatoma cells assessed using the SynergyFinder platform**. **(a)** The dose-response matrix revealed a pronounced reduction in cell viability even at low drug concentrations. A 50% growth inhibition was predicted at 12.5 µM SR9009 combined with 5 µM sorafenib. **(b)** Drug interaction analysis based on the **Highest Single Agent (HSA)** model demonstrated a highly significant synergy score (p = 2.55 × 10⁻¹³⁶). The 3D synergy surface highlights synergistic (red) and antagonistic (green) dose regions. According to the HSA model, a synergy score >10 indicates a biologically relevant synergistic interaction between the two agents. |

| 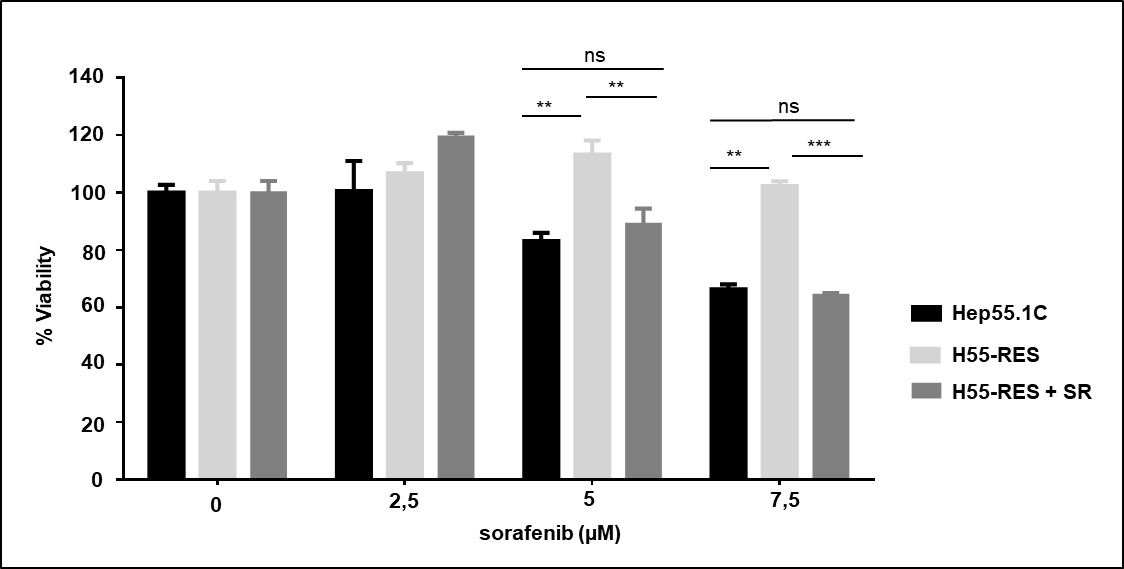 |
| --- |
| **Supplementary Fig. 5. SR9009 enhances sorafenib-induced cytotoxicity in sorafenib-resistant H55-RES cells.** Wild-type Hep55.1C and sorafenib-resistant (H55-RES) cells were treated for 48 hours with increasing concentrations of sorafenib, either alone or in combination with SR9009 (12.5 µM). Cell viability was quantified relative to untreated controls. The combination treatment significantly reduced viability in both wild-type and resistant cells, indicating that SR9009 restores sensitivity to sorafenib. **p ≤ 0.01; *****p ≤ 0.001. Data are presented as mean ± SD.** |

| 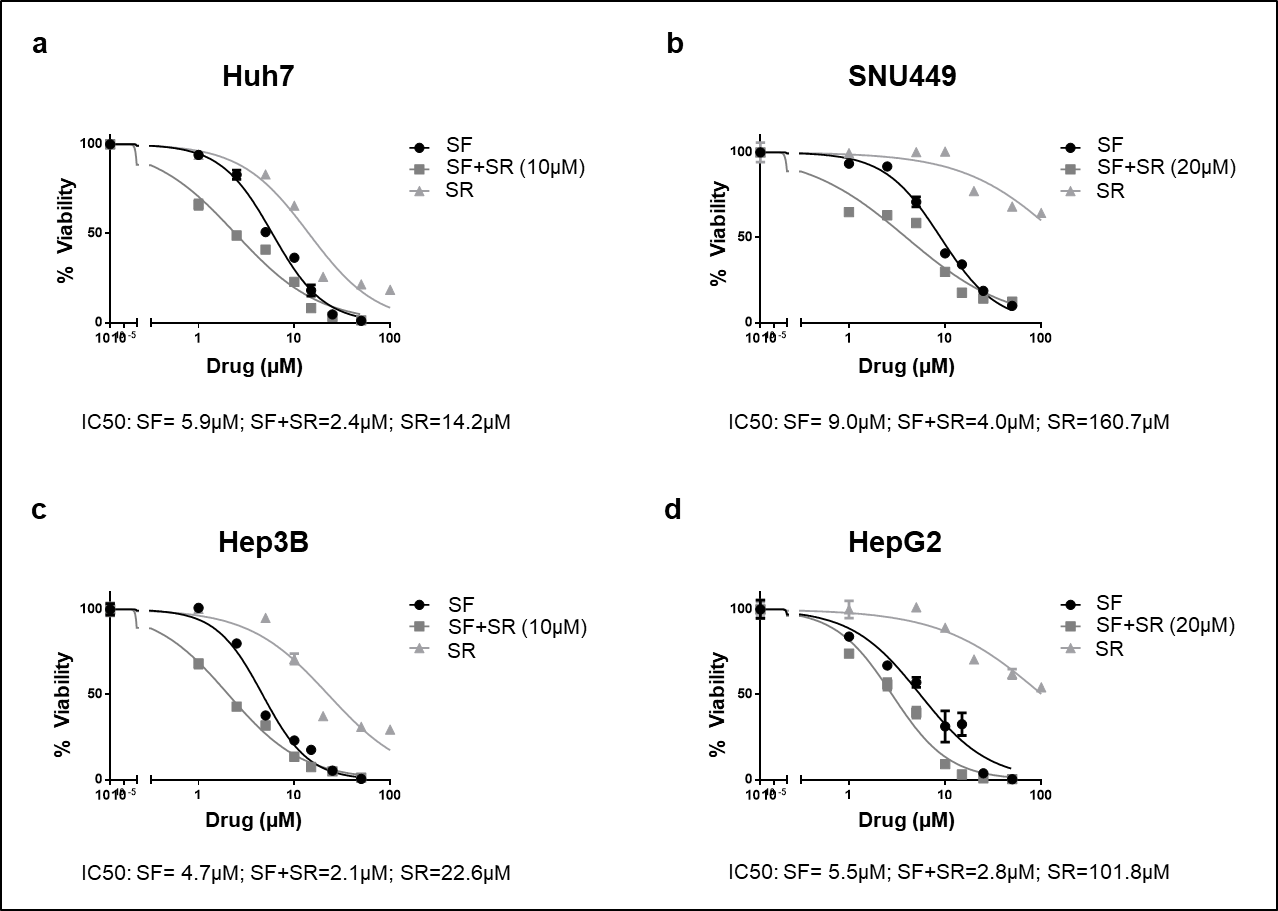 |
| --- |
| **Supplementary Fig. 6.** **Synergistic effect of sorafenib and SR9009 on cell viability confirmed in human HCC cell lines.** Human HCC cell lines Huh7 **(a)**, SNU449 **(b)**, Hep3B **(c)**, and HepG2 **(d)** were treated for 48 hours with increasing concentrations of sorafenib (SF) or SR9009 (SR), alone or in combination. The combination treatment markedly enhanced sorafenib sensitivity across all cell lines, as reflected by a significant reduction in sorafenib IC₅₀ values compared to sorafenib alone, indicating a strong synergistic interaction between the two compounds. Drug concentrations [µM] are plotted on a log₁₀ scale. Data points represent mean cell viability (± SD) at each concentration. |

| 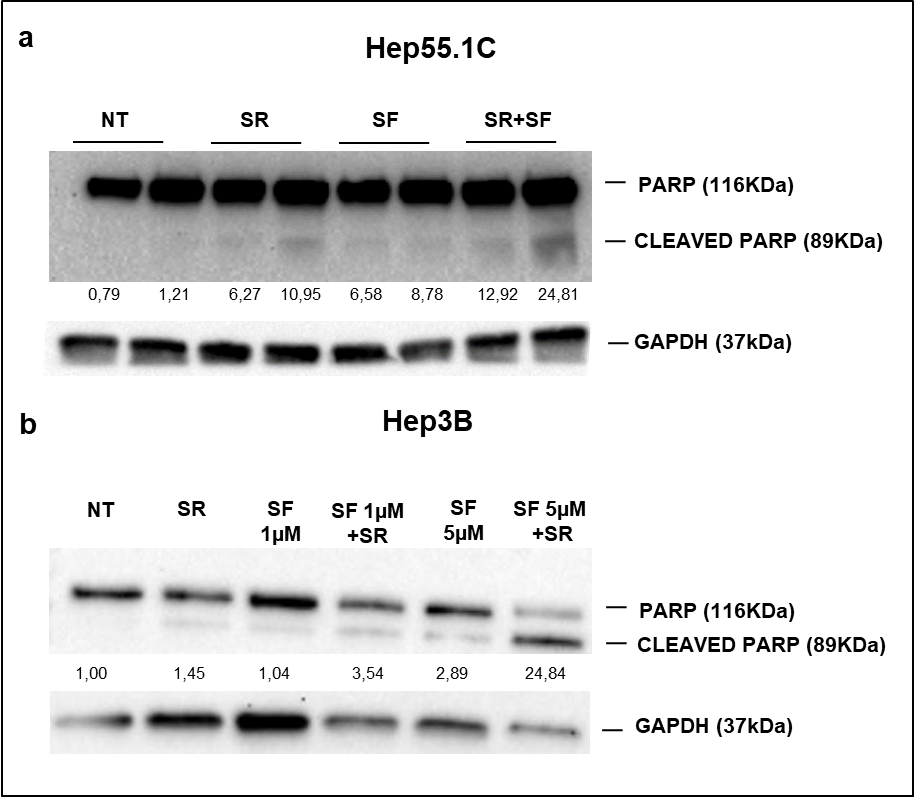 |
| --- |
| **Supplementary Fig. 7.** **Combined treatment with sorafenib and SR9009 enhances apoptosis in vitro**. Western blot analysis and densitometric quantification of cleaved PARP were performed in murine Hep55.1C **(a)** and human Hep3B **(b)** cells after 48 hours of treatment. The combination of sorafenib (SF) and SR9009 (SR, 12.5 µM) markedly increased PARP cleavage compared to single-agent treatments, indicating enhanced apoptotic activity. Protein levels were normalized to GAPDH and expressed relative to untreated controls (NT). |

| 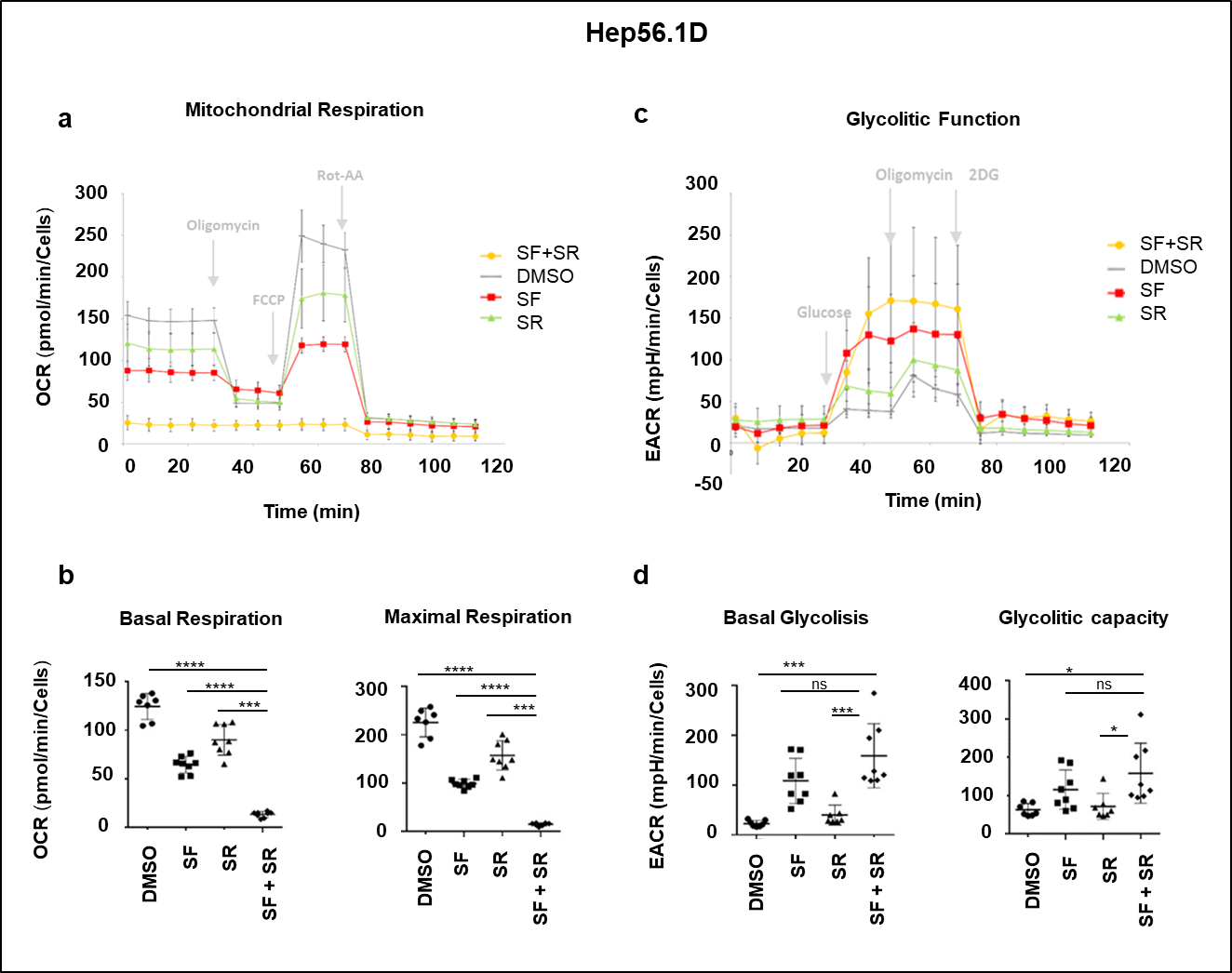 |
| --- |
| **Supplementary Fig. 8. Combined treatment with sorafenib and SR9009 impairs mitochondrial respiration in Hep56.1D cells**. Mitochondrial oxidative phosphorylation (OxPhos) and glycolysis were assessed in Hep56.1D cells under the following conditions: (i) sorafenib (SF, 7.5 µM); (ii) SR9009 (SR, 12.5 µM); (iii) combination of sorafenib and SR9009 (SF+SR); and (iv) DMSO control. **(a)** OxPhos activity was measured as oxygen consumption rate (OCR) using a Seahorse XFe/XF Extracellular Flux Analyzer under basal conditions and following sequential injections of oligomycin, FCCP (carbonyl cyanide-4-(trifluoromethoxy)phenylhydrazone), and a rotenone/antimycin A mixture (Rot/AA). **(b)** Both basal and maximal respiration were profoundly reduced in cells treated with SF+SR, indicating severe mitochondrial dysfunction. **(c)** Glycolytic function was assessed by measuring the extracellular acidification rate (ECAR). **(d)** Combined treatment resulted in a significant increase in both basal glycolysis and glycolytic capacity compared to control, reflecting a metabolic shift toward glycolytic dependence. Data are presented as mean ± SD. *p ≤ 0.05; ***p ≤ 0.001; ****p ≤ 0.0001. |

| 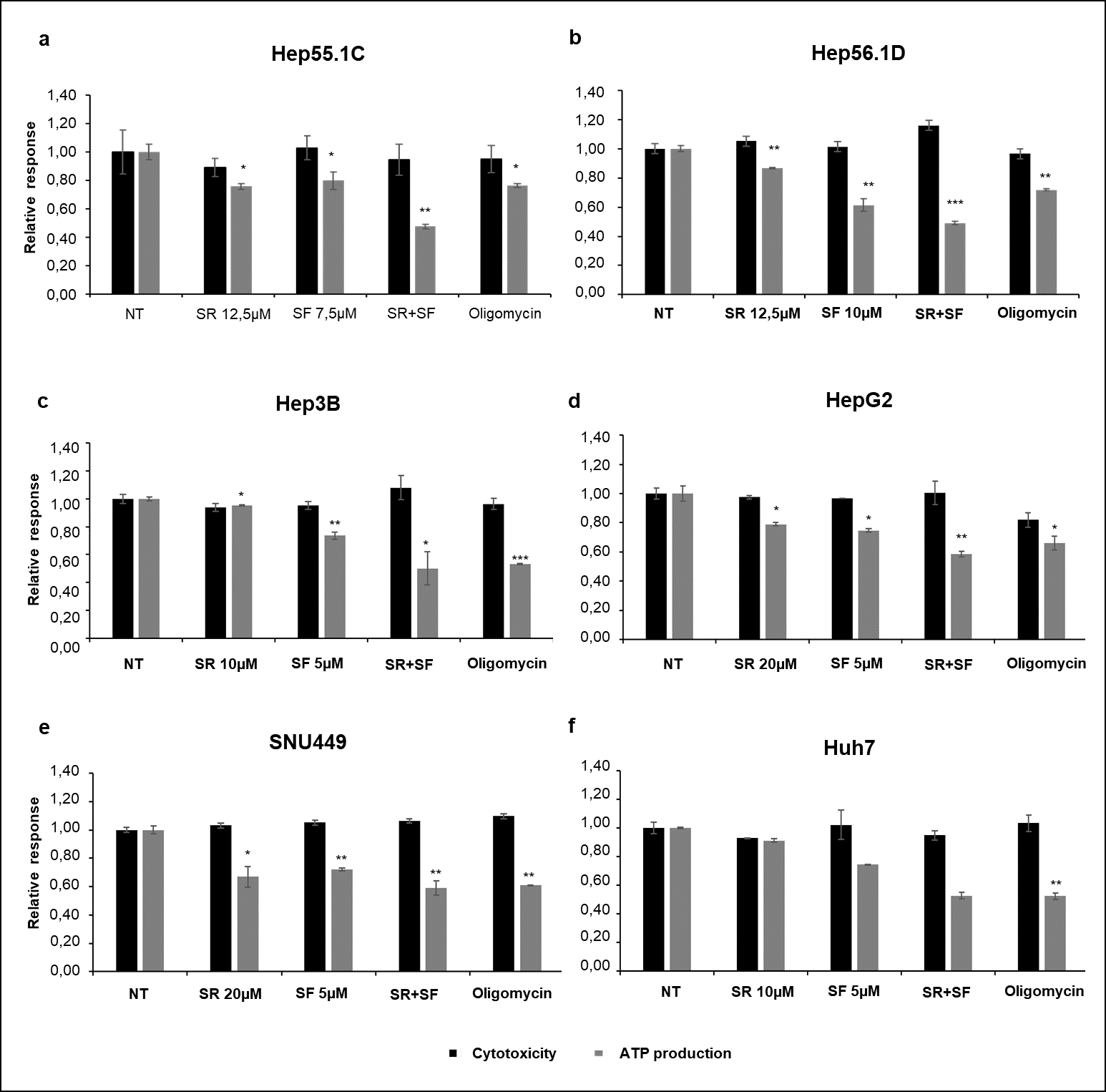 |
| --- |
| **Supplementary Fig. 9.** **The combination of sorafenib and SR9009 inhibits mitochondrial ATP production in murine as well as in human HCC cell lines**. Murine hepatoma cell lines Hep55.1C **(a)** and Hep56.1D **(b**), together with human HCC cell lines Hep3B **(c**), HepG2 **(d**), SNU449 **(e)**, and Huh7 **(f)**, were treated under the following conditions: untreated (NT), sorafenib (SF), SR9009 (SR), or their combination (SF+SR). Mitochondrial function was evaluated using the Mitochondrial ToxGlo™ assay, which simultaneously measures cellular ATP production (luminescent signal) and cytotoxicity (fluorescent signal at 485 nmEx/530 nmEm). Data are expressed as the ratio of each treatment condition to the mean value of the untreated control. Oligomycin, a potent ATP synthase inhibitor, was used as a positive control. Combined treatment with sorafenib and SR9009 markedly reduced mitochondrial ATP levels across all tested cell lines, consistent with a synergistic impairment of mitochondrial function. Data are presented as mean ± SD. *p ≤ 0.05; **p ≤ 0.01; ***p ≤ 0.001. |

| 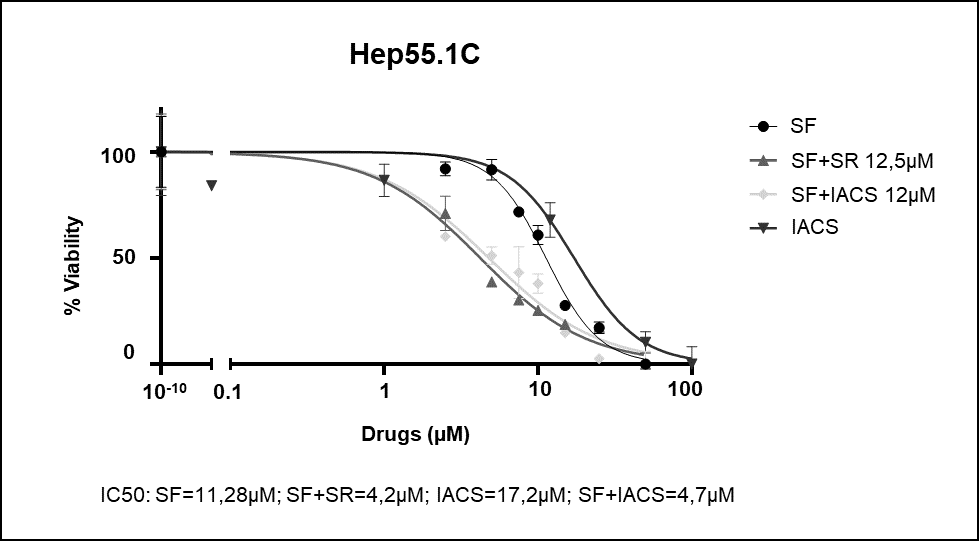 |
| --- |
| **Supplementary Fig. 10.** **Inhibition of mitochondrial respiration by IACS-010759 enhances sorafenib cytotoxicity in Hep55.1C cells**. Dose–response analyses were performed to determine the IC₅₀ values of sorafenib (SF) and the mitochondrial complex I inhibitor IACS-010759 (IACS) in Hep55.1C cells. Cells were treated with increasing concentrations of sorafenib alone, IACS alone, or sorafenib in combination with a fixed concentration of IACS-010759 (12 µM). SR9009 (12.5 µM) was included as a reference compound. Cell viability was assessed 48 hours after treatment and normalized to the mean viability of untreated cells (NT). The combination of sorafenib with IACS-010759 significantly reduced cell viability, resulting in a leftward shift of the sorafenib dose–response curve (sorafenib IC₅₀: values from 11,28 µM to 4,7 µM), comparable to that observed with the sorafenib + SR9009 combination (sorafenib IC₅₀: values from 11,28 µM to 4,2 µM). Drug concentrations [µM] are shown on a log₁₀ scale. Data points represent mean values ± SD from three independent experiments. |
|  |

| 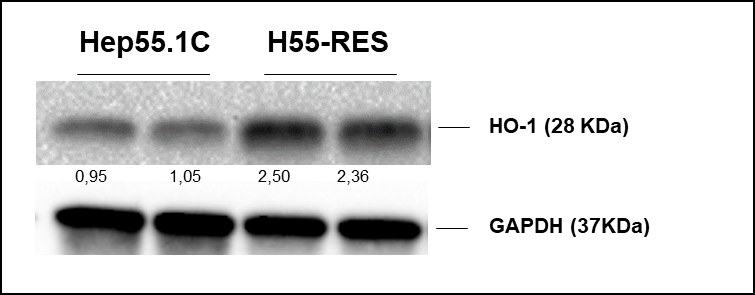 |
| --- |
| **Supplementary Fig. 11.** **Heme oxygenase-1 expression is upregulated in sorafenib-resistant cells.** Western blot analysis and densitometric quantification of Heme oxygenase-1 (HO-1) protein levels in sorafenib-sensitive Hep55.1C cells and sorafenib-resistant H55-RES cells. Protein expression levels were normalized to GAPDH and expressed relative to the mean value of untreated Hep55.1C cells. The results indicate a marked increase in HO-1 abundance in resistant cells compared to sensitive counterparts, consistent with an adaptive cytoprotective response to chronic sorafenib exposure. |

| 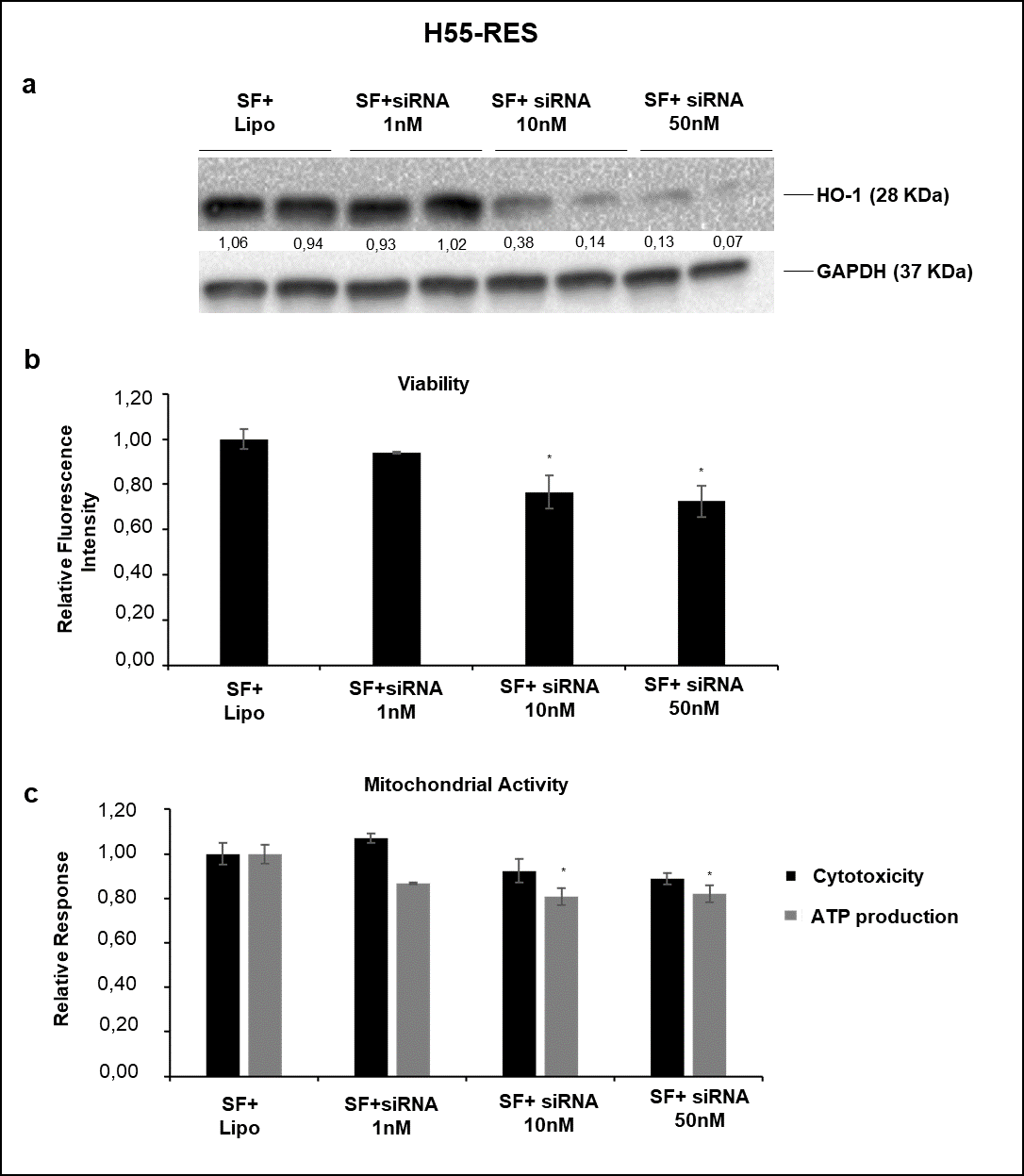 |
| --- |
| **Supplementary Fig. 12.** ***Hmox1* knockdown increases the sensitivity of sorafenib-resistant H55-RES cells to sorafenib.** To assess the role of *Hmox1* in sorafenib resistance, H55-RES cells were transfected with increasing concentrations of siRNA targeting *Hmox1* (1 nM, 10 nM, 50 nM) and collected after 24 hours. **(a)** Efficient Hmox1 silencing was confirmed by Western blot analysis of HO-1 protein levels, normalized to GAPDH and expressed relative to control cells treated with Lipofectamine and sorafenib (SF+Lipo). *Hmox1* knockdown led to a concentration-dependent decrease in both cell viability **(b)** and mitochondrial activity (c) compared with control, indicating that HO-1 contributes to the adaptive resistance phenotype. Data are presented as mean ± SD. *p ≤ 0.05. |

| 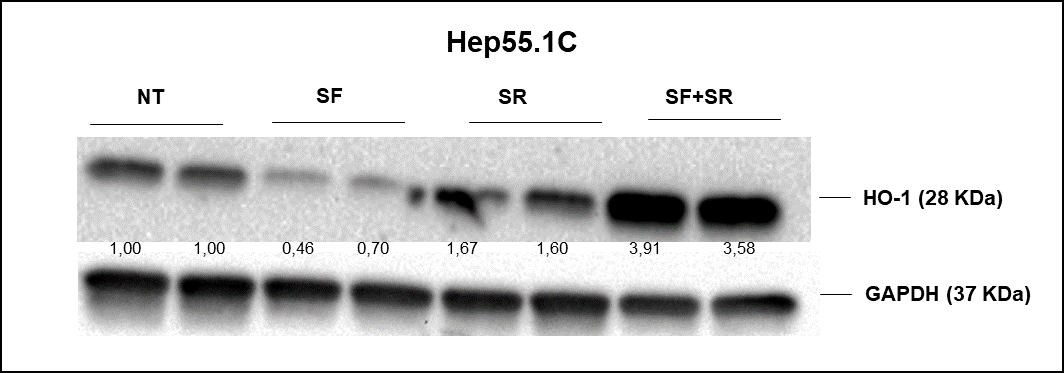 |
| --- |
| **Supplementary Fig. 13.** **Combined treatment with sorafenib and SR9009 increases HO-1 protein levels in Hep55.1C cells.** Western blot analysis and quantification of HO-1 expression in Hep55.1C cells treated with sorafenib (SF), SR9009 (SR), or their combination (SR+SF), compared with untreated controls (NT). HO-1 protein levels were normalized to GAPDH and expressed relative to untreated cells. The combination treatment elicited a pronounced induction of HO-1 compared with single-agent treatments, consistent with activation of a cellular stress response mechanism. |

| 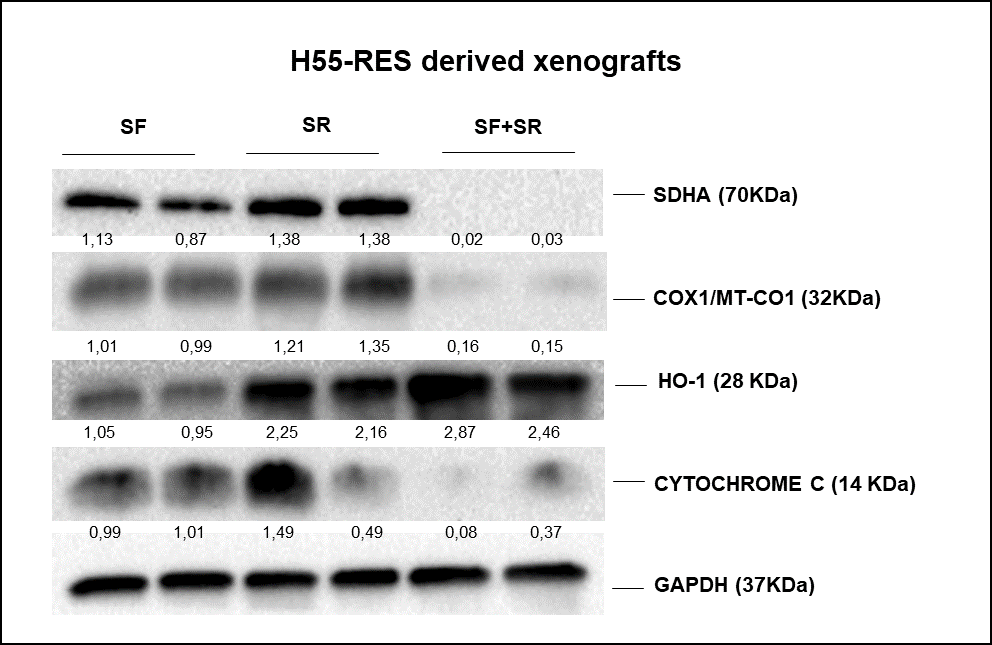 |
| --- |
| **Supplementary Fig. 14.** **Combined treatment with sorafenib and SR9009 reduces mitochondrial respiratory complex components and increases HO-1 protein levels *in vivo***. Western blot analysis was performed on xenograft tumors derived from H55-RES cells treated with sorafenib (SF), SR9009 (SR), or their combination (SF+SR). Protein levels of key components of the mitochondrial electron transport chain, including succinate dehydrogenase subunit A (SDHA), cytochrome c oxidase I (COX1/MT-CO1) and cytochrome c, were markedly reduced in tumors treated with the combination compared to single-agent treatments. Conversely, HO-1 protein levels were significantly upregulated upon combined treatment, suggesting the activation of an adaptive stress response. All values were normalized to GAPDH and expressed relative to untreated controls. |
