# Supplementary material for: Targeting metabolic vulnerabilities: REV-ERB agonist SR9009 potentiates sorafenib efficacy in liver cancer
Source: Cell Death Discov. 2026 Jan 19;12:86. doi: 10.1038/s41420-026-02940-3 (PMC12877144; doi:10.1038/s41420-026-02940-3)
Supplement: Supplementary file 2 — Supplementary Table 1 [file 41420_2026_2940_MOESM2_ESM.docx]

**Supplementary Table 1.** Normalized expression levels of genes belonging to the HALLMARK_OXIDATIVE_PHOSPHORYLATION gene set.

| **Gene symbol** | **Encoded protein** |  | **Average difference (H55-RES vs NT)** | **Average FoldChange (H55-RES vs NT)** | **Regulation** | **Benjamini-Hochberg adjusted p-value** |  | **H55 (NT1)** | **H55 (NT2)** | **H55-RES (191)** | **H55-RES (412)** | **H55-RES (422)** | **H55-RES (423)** | **H55-RES (424)** |
| --- | --- | --- | --- | --- | --- | --- | --- | --- | --- | --- | --- | --- | --- | --- |
|  |  |  |  |  |  |  |  |  |  |  |  |  |  |  |
| Uqcrq | ubiquinol-cytochrome c reductase, complex III subunit VII |  | 450,09 | 7,59 | UP | 2,87E-11 |  | 93,61 | 38,73 | 440,15 | 414,20 | 542,40 | 709,82 | 474,74 |
| Vdac3 | voltage-dependent anion channel 3 |  | 635,18 | 2,65 | UP | 7,14E-10 |  | 396,04 | 372,73 | 1143,22 | 900,94 | 958,45 | 975,85 | 1119,37 |
| Atp6v1g1 | ATPase, H+ transporting, lysosomal V1 subunit G1 |  | 561,98 | 3,97 | UP | 1,62E-08 |  | 216,02 | 159,74 | 623,28 | 545,92 | 774,67 | 963,07 | 842,34 |
| Hspa9 | heat shock protein 9 |  | 5.649,89 | 4,73 | UP | 1,10E-07 |  | 1850,59 | 1176,29 | 6202,47 | 4281,40 | 6457,10 | 11871,14 | 7004,52 |
| Cyc1 | cytochrome c-1 |  | 271,84 | 4,23 | UP | 2,49E-07 |  | 93,61 | 72,61 | 263,82 | 373,03 | 331,82 | 472,82 | 333,27 |
| Aco2 | aconitase 2, mitochondrial |  | 1.289,93 | 3,24 | UP | 2,87E-07 |  | 633,67 | 513,11 | 1413,84 | 1462,29 | 1942,42 | 2613,89 | 1884,17 |
| Opa1 | OPA1, mitochondrial dynamin like GTPase |  | 565,60 | 2,48 | UP | 5,19E-07 |  | 356,44 | 411,46 | 878,04 | 756,87 | 1030,56 | 1065,31 | 1016,97 |
| Ndufb4 | NADH:ubiquinone oxidoreductase subunit B4 |  | 198,62 | 3,20 | UP | 1,37E-06 |  | 108,01 | 67,77 | 243,42 | 283,51 | 301,19 | 307,86 | 296,57 |
| Got2 | glutamatic-oxaloacetic transaminase 2, mitochondrial |  | 823,39 | 1,95 | UP | 2,10E-06 |  | 820,89 | 914,89 | 1537,13 | 1520,95 | 1741,42 | 1870,97 | 1785,91 |
| Nnt | nicotinamide nucleotide transhydrogenase |  | 209,96 | 273,42 | UP | 2,43E-06 |  | 0,00 | 0,00 | 113,32 | 343,71 | 96,99 | 170,19 | 325,57 |
| Ndufa8 | NADH:ubiquinone oxidoreductase subunit A8 |  | 198,04 | 2,97 | UP | 5,13E-06 |  | 126,01 | 67,77 | 269,71 | 289,17 | 292,90 | 332,25 | 290,65 |
| Atp6v1c1 | ATPase, H+ transporting, lysosomal V1 subunit C1 |  | 359,69 | 2,11 | UP | 1,05E-05 |  | 381,64 | 256,56 | 697,63 | 635,44 | 706,39 | 676,13 | 678,37 |
| Mfn2 | mitofusin 2 |  | 271,34 | 2,30 | UP | 1,34E-05 |  | 219,62 | 193,63 | 446,05 | 422,43 | 492,63 | 468,76 | 559,98 |
| Pmpca | peptidase (mitochondrial processing) alpha |  | 307,92 | 1,91 | UP | 2,13E-05 |  | 356,44 | 314,65 | 667,25 | 625,15 | 689,17 | 633,72 | 602,01 |
| Cyb5r3 | cytochrome b5 reductase 3 |  | 879,27 | 2,30 | UP | 3,63E-05 |  | 828,09 | 513,11 | 1721,17 | 1331,09 | 1364,29 | 1513,15 | 1819,65 |
| Uqcrc2 | ubiquinol cytochrome c reductase core protein 2 |  | 317,75 | 2,27 | UP | 4,37E-05 |  | 280,83 | 212,99 | 555,74 | 459,48 | 554,52 | 661,02 | 592,54 |
| Ndufs6 | NADH:ubiquinone oxidoreductase core subunit S6 |  | 98,60 | 4,97 | UP | 5,46E-05 |  | 18,00 | 33,88 | 135,99 | 135,84 | 100,18 | 137,67 | 113,06 |
| Mdh2 | malate dehydrogenase 2, NAD (mitochondrial) |  | 650,94 | 2,31 | UP | 6,11E-05 |  | 626,47 | 353,37 | 1005,87 | 1129,39 | 1139,04 | 1362,13 | 1067,87 |
| Mrps15 | mitochondrial ribosomal protein S15 |  | 145,47 | 4,01 | UP | 8,12E-05 |  | 32,40 | 67,77 | 196,73 | 137,89 | 210,58 | 254,42 | 178,18 |
| Sdhb | succinate dehydrogenase complex, subunit B, iron sulfur (Ip) |  | 463,51 | 3,42 | UP | 8,23E-05 |  | 198,02 | 183,95 | 468,26 | 430,15 | 867,84 | 945,07 | 561,17 |
| Cox8a | cytochrome c oxidase subunit 8A |  | 678,81 | 3,47 | UP | 8,56E-05 |  | 324,03 | 222,67 | 563,00 | 761,50 | 1204,12 | 1430,67 | 801,50 |
| Cycs | cytochrome c, somatic |  | 381,41 | 2,20 | UP | 8,94E-05 |  | 338,44 | 295,28 | 541,69 | 798,04 | 692,99 | 802,76 | 655,88 |
| Cox6a1 | cytochrome c oxidase subunit 6A1 |  | 333,05 | 3,37 | UP | 1,10E-04 |  | 158,42 | 121,02 | 336,80 | 328,78 | 621,52 | 652,31 | 424,43 |
| Cox7a2 | cytochrome c oxidase subunit 7A2 |  | 142,09 | 2,86 | UP | 1,37E-04 |  | 82,81 | 67,77 | 168,17 | 208,38 | 237,38 | 247,45 | 225,53 |
| Atp5pb | ATP synthase peripheral stalk-membrane subunit b |  | 347,05 | 2,02 | UP | 1,84E-04 |  | 388,84 | 285,60 | 649,12 | 784,14 | 750,42 | 626,75 | 610,89 |
| Cox6c | cytochrome c oxidase subunit 6C |  | 191,57 | 2,19 | UP | 2,12E-04 |  | 144,02 | 183,95 | 341,79 | 380,75 | 333,73 | 408,35 | 313,14 |
| Ndufs8 | NADH:ubiquinone oxidoreductase core subunit S8 |  | 154,53 | 3,58 | UP | 3,19E-04 |  | 61,21 | 58,09 | 142,79 | 182,66 | 268,01 | 292,18 | 185,28 |
| Ogdh | oxoglutarate (alpha-ketoglutarate) dehydrogenase (lipoamide) |  | 668,13 | 2,19 | UP | 4,06E-04 |  | 597,66 | 522,80 | 955,55 | 1041,41 | 1360,46 | 1652,56 | 1131,80 |
| Cox5b | cytochrome c oxidase subunit 5B |  | 186,56 | 2,38 | UP | 4,72E-04 |  | 115,21 | 159,74 | 287,39 | 301,00 | 379,68 | 382,21 | 269,93 |
| Tomm22 | translocase of outer mitochondrial membrane 22 |  | 208,01 | 2,31 | UP | 6,82E-04 |  | 205,22 | 101,65 | 334,99 | 394,13 | 404,57 | 315,41 | 358,13 |
| Gpx4 | glutathione peroxidase 4 |  | 523,82 | 2,66 | UP | 6,95E-04 |  | 360,04 | 266,24 | 588,38 | 634,93 | 718,52 | 1205,30 | 1037,68 |
| Nqo2 | N-ribosyldihydronicotinamide quinone reductase 2 |  | 249,60 | 3,40 | UP | 8,57E-04 |  | 158,42 | 43,57 | 403,89 | 286,59 | 239,29 | 414,16 | 409,04 |
| Uqcr10 | ubiquinol-cytochrome c reductase, complex III subunit X |  | 139,10 | 3,39 | UP | 1,03E-03 |  | 54,01 | 62,93 | 145,51 | 135,84 | 287,15 | 236,41 | 182,91 |
| Hccs | holocytochrome c synthetase |  | 116,82 | 2,90 | UP | 1,05E-03 |  | 72,01 | 48,41 | 154,57 | 192,95 | 209,94 | 199,24 | 128,45 |
| Ndufb1 | NADH:ubiquinone oxidoreductase subunit B1 |  | 119,58 | 3,27 | UP | 1,07E-03 |  | 75,61 | 24,20 | 163,19 | 162,08 | 227,81 | 147,54 | 146,80 |
| Cox5a | cytochrome c oxidase subunit 5A |  | 204,71 | 2,62 | UP | 1,19E-03 |  | 176,42 | 67,77 | 296,00 | 294,31 | 324,16 | 417,64 | 301,89 |
| Cox6b1 | cytochrome c oxidase, subunit 6B1 |  | 375,51 | 2,22 | UP | 1,31E-03 |  | 403,24 | 203,31 | 590,19 | 636,47 | 827,64 | 742,93 | 596,68 |
| Pdhb | pyruvate dehydrogenase (lipoamide) beta |  | 307,69 | 2,48 | UP | 1,42E-03 |  | 219,62 | 193,63 | 402,98 | 346,79 | 630,46 | 694,71 | 496,64 |
| Cs | citrate synthase |  | 875,17 | 2,19 | UP | 1,95E-03 |  | 799,28 | 672,86 | 1330,43 | 1556,45 | 1107,13 | 2313,59 | 1748,61 |
| Slc25a5 | solute carrier family 25 (mitochondrial carrier, adenine nucleotide translocator), member 5 |  | 430,19 | 1,89 | UP | 2,34E-03 |  | 579,66 | 372,73 | 848,12 | 1061,48 | 756,17 | 850,97 | 1015,19 |
| Atp6v0e | ATPase, H+ transporting, lysosomal V0 subunit E |  | 441,17 | 2,67 | UP | 2,66E-03 |  | 298,83 | 227,51 | 517,67 | 419,86 | 773,40 | 1115,84 | 694,95 |
| Dlat | dihydrolipoamide S-acetyltransferase |  | 184,84 | 1,71 | UP | 3,08E-03 |  | 252,03 | 271,08 | 468,71 | 443,52 | 381,59 | 469,92 | 468,23 |
| Sdha | succinate dehydrogenase complex, subunit A, flavoprotein (Fp) |  | 258,91 | 1,63 | UP | 3,10E-03 |  | 442,85 | 377,57 | 627,36 | 699,76 | 596,00 | 712,72 | 709,75 |
| Timm8b | translocase of inner mitochondrial membrane 8B |  | 105,29 | 3,22 | UP | 3,15E-03 |  | 50,41 | 43,57 | 116,04 | 97,76 | 179,31 | 213,76 | 154,50 |
| Uqcrc1 | ubiquinol-cytochrome c reductase core protein 1 |  | 330,31 | 2,37 | UP | 3,24E-03 |  | 291,63 | 183,95 | 436,98 | 478,51 | 508,58 | 875,36 | 541,04 |
| Mrps22 | mitochondrial ribosomal protein S22 |  | 102,59 | 2,21 | UP | 3,66E-03 |  | 97,21 | 67,77 | 169,99 | 177,00 | 216,96 | 199,24 | 162,19 |
| Slc25a3 | solute carrier family 25 (mitochondrial carrier, phosphate carrier), member 3 |  | 610,88 | 1,75 | UP | 4,13E-03 |  | 867,69 | 745,47 | 1240,22 | 1113,44 | 1463,20 | 1742,01 | 1528,41 |
| Ndufb6 | NADH:ubiquinone oxidoreductase subunit B6 |  | 134,69 | 3,08 | UP | 5,86E-03 |  | 82,81 | 43,57 | 155,48 | 126,57 | 319,70 | 221,89 | 165,75 |
| Ndufs4 | NADH:ubiquinone oxidoreductase core subunit S4 |  | 108,98 | 2,18 | UP | 6,08E-03 |  | 108,01 | 72,61 | 162,73 | 180,09 | 223,98 | 191,10 | 238,56 |
| Immt | inner membrane protein, mitochondrial |  | -361,99 | -1,45 | DOWN | 6,59E-03 |  | 957,70 | 881,01 | 489,56 | 687,41 | 480,50 | 531,49 | 597,87 |
| Phyh | phytanoyl-CoA hydroxylase |  | 74,59 | 3,00 | UP | 7,18E-03 |  | 36,00 | 38,73 | 105,17 | 90,56 | 109,12 | 166,71 | 88,20 |
| Lrpprc | leucine-rich PPR-motif containing |  | 200,49 | 1,52 | UP | 7,55E-03 |  | 410,44 | 353,37 | 562,09 | 551,06 | 580,69 | 623,85 | 594,32 |
| Atp6v1h | ATPase, H+ transporting, lysosomal V1 subunit H |  | 464,90 | 2,07 | UP | 8,31E-03 |  | 457,25 | 411,46 | 711,68 | 601,49 | 1273,68 | 1065,31 | 844,12 |
| Vdac2 | voltage-dependent anion channel 2 |  | 292,54 | 1,64 | UP | 9,53E-03 |  | 460,85 | 450,19 | 785,57 | 763,05 | 569,84 | 842,25 | 779,60 |
| Grpel1 | GrpE-like 1, mitochondrial |  | 266,25 | 2,42 | UP | 9,89E-03 |  | 158,42 | 217,83 | 314,14 | 302,54 | 705,12 | 559,37 | 390,69 |
| Ndufc1 | NADH:ubiquinone oxidoreductase subunit C1 |  | 111,35 | 2,15 | UP | 9,90E-03 |  | 115,21 | 72,61 | 176,33 | 183,69 | 268,01 | 221,89 | 176,40 |
| Ndufa3 | NADH:ubiquinone oxidoreductase subunit A3 |  | 100,85 | 2,42 | UP | 1,18E-02 |  | 93,61 | 43,57 | 149,14 | 135,32 | 221,43 | 198,66 | 142,66 |
| Ndufa5 | NADH:ubiquinone oxidoreductase subunit A5 |  | 80,57 | 2,81 | UP | 1,24E-02 |  | 64,81 | 19,36 | 140,98 | 150,24 | 127,62 | 110,36 | 84,06 |
| Ndufv2 | NADH:ubiquinone oxidoreductase core subunit V2 |  | 132,42 | 1,85 | UP | 1,24E-02 |  | 180,02 | 125,86 | 277,87 | 323,64 | 235,46 | 329,35 | 260,46 |
| Atp6v1d | ATPase, H+ transporting, lysosomal V1 subunit D |  | 207,29 | 1,86 | UP | 1,27E-02 |  | 223,22 | 261,40 | 384,85 | 364,80 | 605,57 | 489,09 | 403,71 |
| Timm17a | translocase of inner mitochondrial membrane 17a |  | -180,86 | -1,44 | DOWN | 1,29E-02 |  | 381,64 | 532,48 | 276,06 | 289,68 | 308,21 | 230,02 | 277,03 |
| Slc25a4 | solute carrier family 25 (mitochondrial carrier, adenine nucleotide translocator), member 4 |  | 583,80 | 1,70 | UP | 1,30E-02 |  | 957,70 | 701,90 | 1515,83 | 1680,46 | 1280,06 | 1047,88 | 1543,80 |
| Ndufa7 | NADH:ubiquinone oxidoreductase subunit A7 |  | 133,47 | 1,89 | UP | 1,40E-02 |  | 172,82 | 121,02 | 242,97 | 239,26 | 317,78 | 344,45 | 257,50 |
| Ndufa9 | NADH:ubiquinone oxidoreductase subunit A9 |  | 127,83 | 1,63 | UP | 1,61E-02 |  | 183,62 | 227,51 | 297,82 | 348,85 | 375,85 | 318,89 | 325,57 |
| Hsd17b10 | hydroxysteroid (17-beta) dehydrogenase 10 |  | 101,88 | 1,93 | UP | 1,63E-02 |  | 111,61 | 106,50 | 191,29 | 163,11 | 231,00 | 256,16 | 213,10 |
| Ndufb3 | NADH:ubiquinone oxidoreductase subunit B3 |  | 76,32 | 2,23 | UP | 1,73E-02 |  | 75,61 | 43,57 | 114,23 | 107,02 | 148,68 | 154,51 | 155,09 |
| Ndufc2 | NADH:ubiquinone oxidoreductase subunit C2 |  | 143,29 | 1,96 | UP | 1,77E-02 |  | 183,62 | 106,50 | 226,65 | 257,27 | 346,50 | 349,68 | 261,64 |
| Polr2f | polymerase (RNA) II (DNA directed) polypeptide F |  | 63,54 | 2,34 | UP | 1,78E-02 |  | 43,20 | 53,25 | 117,86 | 98,28 | 131,45 | 127,21 | 84,06 |
| Fdx1 | ferredoxin 1 |  | -75,00 | -1,87 | DOWN | 1,80E-02 |  | 122,41 | 188,79 | 68,90 | 94,16 | 81,04 | 80,16 | 78,73 |
| Ndufb2 | NADH:ubiquinone oxidoreductase subunit B2 |  | -93,03 | -2,60 | DOWN | 1,97E-02 |  | 93,61 | 217,83 | 40,34 | 67,40 | 74,02 | 79,58 | 52,09 |
| Idh3b | isocitrate dehydrogenase 3 (NAD+) beta |  | 345,63 | 1,92 | UP | 2,02E-02 |  | 370,84 | 377,57 | 550,30 | 507,84 | 823,17 | 1051,95 | 665,94 |
| Ndufa2 | NADH:ubiquinone oxidoreductase subunit A2 |  | 136,93 | 2,58 | UP | 2,26E-02 |  | 108,01 | 62,93 | 125,56 | 184,20 | 247,59 | 372,92 | 181,73 |
| Afg3l2 | AFG3-like AAA ATPase 2 |  | 353,38 | 1,85 | UP | 2,58E-02 |  | 428,44 | 396,94 | 642,32 | 570,61 | 704,48 | 1190,77 | 722,18 |
| Ndufa6 | NADH:ubiquinone oxidoreductase subunit A6 |  | 219,62 | 2,39 | UP | 2,63E-02 |  | 169,22 | 145,22 | 331,81 | 222,28 | 317,78 | 708,66 | 303,67 |
| Rhot1 | ras homolog family member T1 |  | 228,87 | 1,83 | UP | 2,82E-02 |  | 230,42 | 329,17 | 436,07 | 341,13 | 623,44 | 608,17 | 534,53 |
| Etfb | electron transferring flavoprotein, beta polypeptide |  | 53,41 | 2,07 | UP | 2,96E-02 |  | 57,61 | 38,73 | 110,60 | 102,39 | 86,15 | 103,39 | 105,37 |
| Cyb5a | cytochrome b5 type A (microsomal) |  | -323,44 | -1,48 | DOWN | 3,09E-02 |  | 892,89 | 726,10 | 398,00 | 612,81 | 363,73 | 572,15 | 483,62 |
| Cox7c | cytochrome c oxidase subunit 7C |  | 187,18 | 2,05 | UP | 3,38E-02 |  | 259,23 | 87,13 | 273,79 | 313,86 | 367,55 | 429,26 | 417,32 |
| Etfa | electron transferring flavoprotein, alpha polypeptide |  | 262,76 | 1,76 | UP | 3,55E-02 |  | 478,85 | 198,47 | 583,85 | 648,31 | 536,66 | 625,01 | 613,26 |
| Surf1 | surfeit gene 1 |  | 86,31 | 2,44 | UP | 3,62E-02 |  | 57,61 | 62,93 | 110,60 | 80,27 | 197,82 | 203,30 | 140,88 |
| Eci1 | enoyl-Coenzyme A delta isomerase 1 |  | 51,99 | 2,26 | UP | 3,73E-02 |  | 43,20 | 38,73 | 88,39 | 75,12 | 104,65 | 120,24 | 76,36 |
| Mdh1 | malate dehydrogenase 1, NAD (soluble) |  | 172,27 | 1,54 | UP | 4,00E-02 |  | 367,24 | 261,40 | 413,86 | 536,65 | 518,15 | 526,84 | 437,45 |
| Htra2 | HtrA serine peptidase 2 |  | 63,45 | 1,88 | UP | 4,08E-02 |  | 75,61 | 67,77 | 117,40 | 161,05 | 144,85 | 112,69 | 139,70 |
| Atp6v1f | ATPase, H+ transporting, lysosomal V1 subunit F |  | 122,42 | 2,03 | UP | 4,33E-02 |  | 129,61 | 106,50 | 153,21 | 173,91 | 309,49 | 318,31 | 247,43 |
| Mpc1 | mitochondrial pyruvate carrier 1 |  | 61,69 | 1,88 | UP | 5,05E-02 |  | 72,01 | 67,77 | 103,35 | 144,07 | 119,97 | 163,22 | 127,27 |
| Ndufs7 | NADH:ubiquinone oxidoreductase core subunit S7 |  | 101,87 | 1,81 | UP | 5,11E-02 |  | 122,41 | 130,70 | 164,09 | 191,41 | 266,09 | 297,98 | 222,57 |
| Gpi1 | glucose-6-phosphate isomerase 1 |  | 861,88 | 1,66 | UP | 5,50E-02 |  | 1472,55 | 1137,56 | 1727,97 | 1430,91 | 2522,47 | 2893,29 | 2260,06 |
| Fxn | frataxin |  | -45,95 | -2,52 | DOWN | 5,54E-02 |  | 39,60 | 116,18 | 26,74 | 42,71 | 27,44 | 27,88 | 34,92 |
| Alas1 | aminolevulinic acid synthase 1 |  | 91,91 | 1,83 | UP | 5,79E-02 |  | 136,81 | 77,45 | 190,84 | 172,88 | 259,08 | 151,02 | 221,39 |
| Cox11 | cytochrome c oxidase assembly protein 11, copper chaperone |  | 27,70 | 3,69 | UP | 5,82E-02 |  | 18,00 | 0,00 | 40,80 | 27,78 | 37,01 | 42,98 | 34,92 |
| Mrps12 | mitochondrial ribosomal protein S12 |  | -39,17 | -2,59 | DOWN | 6,00E-02 |  | 90,01 | 43,57 | 19,04 | 42,19 | 29,35 | 22,07 | 25,45 |
| Tomm70a | translocase of outer mitochondrial membrane 70A |  | 382,52 | 1,49 | UP | 7,17E-02 |  | 806,48 | 750,31 | 1329,98 | 1380,48 | 1272,40 | 884,66 | 937,05 |
| Acadsb | acyl-Coenzyme A dehydrogenase, short/branched chain |  | 163,46 | 1,76 | UP | 7,69E-02 |  | 237,62 | 188,79 | 291,92 | 240,29 | 529,00 | 437,97 | 384,17 |
| Retsat | retinol saturase (all trans retinol 13,14 reductase) |  | -52,24 | -2,22 | DOWN | 7,90E-02 |  | 82,81 | 111,34 | 26,74 | 64,32 | 52,96 | 49,95 | 30,19 |
| Ndufb8 | NADH:ubiquinone oxidoreductase subunit B8 |  | 132,01 | 1,56 | UP | 7,96E-02 |  | 237,62 | 232,35 | 336,35 | 341,65 | 301,19 | 504,19 | 351,62 |
| Mrpl34 | mitochondrial ribosomal protein L34 |  | 65,26 | 1,81 | UP | 8,97E-02 |  | 68,41 | 96,81 | 94,29 | 169,28 | 177,40 | 152,77 | 145,62 |
| Aifm1 | apoptosis-inducing factor, mitochondrion-associated 1 |  | -96,70 | -1,37 | DOWN | 9,81E-02 |  | 277,23 | 237,19 | 144,60 | 203,24 | 109,12 | 159,74 | 185,87 |
| Sucla2 | succinate-Coenzyme A ligase, ADP-forming, beta subunit |  | 86,42 | 1,46 | UP | 1,01E-01 |  | 194,42 | 179,11 | 288,75 | 269,61 | 234,19 | 322,38 | 250,99 |
| Echs1 | enoyl Coenzyme A hydratase, short chain, 1, mitochondrial |  | -125,90 | -0,93 | DOWN | 1,01E-01 |  | 428,44 | 479,23 | 316,40 | 393,62 | 283,96 | 315,99 | 329,71 |
| Cox15 | cytochrome c oxidase assembly protein 15 |  | 85,23 | 1,39 | UP | 1,01E-01 |  | 223,22 | 212,99 | 296,46 | 281,96 | 331,18 | 322,38 | 284,73 |
| Ldhd | lactate dehydrogenase D |  | -51,76 | -2,28 | DOWN | 1,06E-01 |  | 108,01 | 82,29 | 29,92 | 19,55 | 53,60 | 50,54 | 63,34 |
| Cox7a2l | cytochrome c oxidase subunit 7A2 like |  | 266,56 | 1,80 | UP | 1,10E-01 |  | 417,64 | 242,03 | 452,39 | 314,38 | 859,54 | 737,12 | 618,59 |
| Hadha | hydroxyacyl-CoA dehydrogenase trifunctional multienzyme complex subunit alpha |  | 156,40 | 1,36 | UP | 1,15E-01 |  | 500,45 | 353,37 | 601,53 | 524,82 | 598,55 | 648,25 | 543,41 |
| Ndufv1 | NADH:ubiquinone oxidoreductase core subunit V1 |  | 98,55 | 1,78 | UP | 1,24E-01 |  | 158,42 | 87,13 | 149,14 | 178,03 | 220,15 | 344,45 | 214,88 |
| Acadvl | acyl-Coenzyme A dehydrogenase, very long chain |  | 91,47 | 1,95 | UP | 1,24E-01 |  | 129,61 | 58,09 | 150,49 | 105,99 | 269,92 | 245,13 | 155,09 |
| Atp1b1 | ATPase, Na+/K+ transporting, beta 1 polypeptide |  | -92,58 | -2,77 | DOWN | 1,24E-01 |  | 154,82 | 145,22 | 77,06 | 111,14 | 11,49 | 37,18 | 50,32 |
| Ndufs3 | NADH:ubiquinone oxidoreductase core subunit S3 |  | 79,79 | 1,66 | UP | 1,26E-01 |  | 129,61 | 111,34 | 168,17 | 157,96 | 254,61 | 262,55 | 158,05 |
| Cox4i1 | cytochrome c oxidase subunit 4I1 |  | 233,25 | 1,55 | UP | 1,36E-01 |  | 435,65 | 416,30 | 937,87 | 713,65 | 699,38 | 441,46 | 503,75 |
| Decr1 | 2,4-dienoyl CoA reductase 1, mitochondrial |  | 66,80 | 1,46 | UP | 1,48E-01 |  | 176,42 | 101,65 | 220,30 | 188,83 | 190,16 | 210,27 | 219,61 |
| Oat | ornithine aminotransferase |  | 95,71 | 1,42 | UP | 1,49E-01 |  | 259,23 | 188,79 | 331,36 | 383,84 | 259,08 | 319,48 | 304,85 |
| Acadm | acyl-Coenzyme A dehydrogenase, medium chain |  | -56,46 | -1,14 | DOWN | 1,70E-01 |  | 183,62 | 164,58 | 114,68 | 150,24 | 88,70 | 116,17 | 118,39 |
| Prdx3 | peroxiredoxin 3 |  | 124,23 | 1,37 | UP | 1,76E-01 |  | 388,84 | 261,40 | 381,22 | 524,82 | 455,61 | 490,25 | 394,83 |
| Cpt1a | carnitine palmitoyltransferase 1a, liver |  | -181,60 | -1,16 | DOWN | 1,80E-01 |  | 586,86 | 508,27 | 305,52 | 561,87 | 293,53 | 277,65 | 391,28 |
| Pdk4 | pyruvate dehydrogenase kinase, isoenzyme 4 |  | -58,88 | -3,05 | DOWN | 1,87E-01 |  | 108,01 | 72,61 | 6,80 | 77,18 | 43,39 | 20,33 | 9,47 |
| Uqcr11 | ubiquinol-cytochrome c reductase, complex III subunit XI |  | 50,97 | 1,69 | UP | 2,01E-01 |  | 93,61 | 48,41 | 84,77 | 96,22 | 123,79 | 173,68 | 131,41 |
| Uqcrfs1 | ubiquinol-cytochrome c reductase, Rieske iron-sulfur polypeptide 1 |  | 74,54 | 1,48 | UP | 2,01E-01 |  | 194,42 | 101,65 | 174,97 | 267,04 | 194,63 | 231,77 | 244,47 |
| Timm9 | translocase of inner mitochondrial membrane 9 |  | -38,39 | -1,07 | DOWN | 2,04E-01 |  | 133,21 | 116,18 | 87,49 | 103,94 | 81,68 | 73,19 | 85,24 |
| Timm13 | translocase of inner mitochondrial membrane 13 |  | 37,06 | 1,67 | UP | 2,12E-01 |  | 50,41 | 62,93 | 82,95 | 84,38 | 77,85 | 141,15 | 82,28 |
| Mrpl35 | mitochondrial ribosomal protein L35 |  | 60,69 | 1,37 | UP | 2,15E-01 |  | 172,82 | 145,22 | 214,41 | 196,04 | 262,27 | 235,83 | 190,02 |
| Slc25a11 | solute carrier family 25 (mitochondrial carrier oxoglutarate carrier), member 11 |  | 79,93 | 1,57 | UP | 2,22E-01 |  | 198,02 | 72,61 | 229,82 | 283,51 | 209,94 | 178,91 | 174,03 |
| Arfip2 | ARF interacting protein 2 |  | 88,79 | 1,51 | UP | 2,57E-01 |  | 187,22 | 154,90 | 196,28 | 177,00 | 297,36 | 404,28 | 224,35 |
| Atp6v0b | ATPase, H+ transporting, lysosomal V0 subunit B |  | 64,04 | 1,46 | UP | 2,69E-01 |  | 151,22 | 121,02 | 150,04 | 189,35 | 163,36 | 299,15 | 198,89 |
| Tcirg1 | T cell, immune regulator 1, ATPase, H+ transporting, lysosomal V0 protein A3 |  | 135,53 | 1,70 | UP | 2,95E-01 |  | 201,62 | 183,95 | 134,18 | 192,95 | 356,71 | 652,31 | 305,45 |
| Supv3l1 | suppressor of var1, 3-like 1 (S. cerevisiae) |  | 36,56 | 1,36 | UP | 3,05E-01 |  | 79,21 | 140,38 | 162,73 | 134,29 | 138,47 | 139,99 | 156,27 |
| Pdha1 | pyruvate dehydrogenase E1 alpha 1 |  | 372,00 | 1,36 | UP | 3,09E-01 |  | 1357,34 | 692,22 | 1596,52 | 1578,58 | 938,03 | 1248,86 | 1621,94 |
| Ech1 | enoyl coenzyme A hydratase 1, peroxisomal |  | 49,99 | 1,41 | UP | 3,25E-01 |  | 97,21 | 154,90 | 121,03 | 166,19 | 166,55 | 235,83 | 190,61 |
| Ndufa1 | NADH:ubiquinone oxidoreductase subunit A1 |  | 36,60 | 1,38 | UP | 3,38E-01 |  | 100,81 | 87,13 | 100,18 | 151,27 | 146,13 | 148,12 | 107,14 |
| Idh3a | isocitrate dehydrogenase 3 (NAD+) alpha |  | 112,06 | 1,42 | UP | 3,46E-01 |  | 277,23 | 261,40 | 363,09 | 629,78 | 234,83 | 299,73 | 379,44 |
| Slc25a12 | solute carrier family 25 (mitochondrial carrier, Aralar), member 12 |  | 43,05 | 1,28 | UP | 3,56E-01 |  | 136,81 | 188,79 | 181,32 | 200,15 | 239,93 | 218,41 | 189,42 |
| Acaa1a | acetyl-Coenzyme A acyltransferase 1A |  | 53,91 | 1,42 | UP | 3,65E-01 |  | 169,22 | 72,61 | 138,71 | 121,94 | 202,92 | 200,98 | 209,55 |
| Mtx2 | metaxin 2 |  | 38,76 | 1,25 | UP | 3,86E-01 |  | 158,42 | 145,22 | 179,51 | 220,22 | 204,20 | 170,19 | 178,77 |
| Atp6ap1 | ATPase, H+ transporting, lysosomal accessory protein 1 |  | 149,30 | 1,41 | UP | 3,87E-01 |  | 442,85 | 271,08 | 332,72 | 397,22 | 421,16 | 475,15 | 905,09 |
| Casp7 | caspase 7 |  | 49,16 | 1,44 | UP | 4,04E-01 |  | 90,01 | 140,38 | 144,60 | 81,30 | 195,26 | 224,79 | 175,81 |
| Idh3g | isocitrate dehydrogenase 3 (NAD+), gamma |  | 59,22 | 1,23 | UP | 4,08E-01 |  | 284,43 | 217,83 | 294,64 | 312,32 | 253,97 | 371,17 | 319,65 |
| Idh1 | isocitrate dehydrogenase 1 (NADP+), soluble |  | -88,90 | -0,77 | DOWN | 4,08E-01 |  | 504,05 | 266,24 | 239,79 | 412,14 | 293,53 | 245,71 | 290,05 |
| Uqcrh | ubiquinol-cytochrome c reductase hinge protein |  | -53,04 | -0,75 | DOWN | 4,38E-01 |  | 288,03 | 183,95 | 141,88 | 180,60 | 155,70 | 263,71 | 172,85 |
| Abcb7 | ATP-binding cassette, sub-family B member 7 |  | 65,81 | 1,19 | UP | 4,50E-01 |  | 370,84 | 285,60 | 366,72 | 475,43 | 346,50 | 365,36 | 416,14 |
| Atp6v0c | ATPase, H+ transporting, lysosomal V0 subunit C |  | 67,05 | 1,22 | UP | 4,99E-01 |  | 309,63 | 295,28 | 354,93 | 281,96 | 389,89 | 510,58 | 310,18 |
| Timm50 | translocase of inner mitochondrial membrane 50 |  | 28,21 | 1,46 | UP | 5,19E-01 |  | 14,40 | 121,02 | 87,49 | 101,36 | 96,36 | 112,69 | 81,69 |
| Aldh6a1 | aldehyde dehydrogenase family 6, subfamily A1 |  | 50,62 | 1,43 | UP | 5,32E-01 |  | 115,21 | 121,02 | 114,23 | 55,57 | 289,70 | 217,82 | 166,34 |
| Mtrf1 | mitochondrial translational release factor 1 |  | -12,83 | -1,07 | DOWN | 5,56E-01 |  | 32,40 | 48,41 | 40,34 | 24,18 | 35,73 | 13,36 | 24,27 |
| Idh2 | isocitrate dehydrogenase 2 (NADP+), mitochondrial |  | 84,49 | 1,22 | UP | 5,60E-01 |  | 486,05 | 275,92 | 390,29 | 603,03 | 320,33 | 512,32 | 501,38 |
| Mtrr | 5-methyltetrahydrofolate-homocysteine methyltransferase reductase |  | 21,25 | 1,27 | UP | 5,82E-01 |  | 46,80 | 130,70 | 129,19 | 86,96 | 123,79 | 96,42 | 113,65 |
| Fh1 | fumarate hydratase 1 |  | -31,16 | -0,40 | DOWN | 5,82E-01 |  | 280,83 | 222,67 | 208,06 | 253,15 | 193,99 | 235,83 | 211,92 |
| Atp6v1e1 | ATPase, H+ transporting, lysosomal V1 subunit E1 |  | 96,57 | 1,17 | UP | 5,86E-01 |  | 651,67 | 484,07 | 556,65 | 584,51 | 857,63 | 785,91 | 537,49 |
| Bckdha | branched chain ketoacid dehydrogenase E1, alpha polypeptide |  | 22,19 | 1,34 | UP | 5,92E-01 |  | 79,21 | 43,57 | 69,81 | 38,59 | 116,78 | 108,04 | 84,65 |
| Pdhx | pyruvate dehydrogenase complex, component X |  | -18,49 | -0,64 | DOWN | 5,93E-01 |  | 79,21 | 101,65 | 83,86 | 83,87 | 72,11 | 45,31 | 74,59 |
| Acat1 | acetyl-Coenzyme A acetyltransferase 1 |  | 25,38 | 1,15 | UP | 6,04E-01 |  | 162,02 | 198,47 | 227,10 | 188,32 | 192,07 | 231,18 | 189,42 |
| Oxa1l | oxidase assembly 1-like |  | 41,00 | 1,15 | UP | 6,35E-01 |  | 234,02 | 329,17 | 279,23 | 255,21 | 321,61 | 413,00 | 343,92 |
| Mgst3 | microsomal glutathione S-transferase 3 |  | 20,04 | 1,28 | UP | 6,38E-01 |  | 46,80 | 106,50 | 82,95 | 146,13 | 58,71 | 76,67 | 118,98 |
| Slc25a20 | solute carrier family 25 (mitochondrial carnitine/acylcarnitine translocase), member 20 |  | -15,44 | -0,53 | DOWN | 6,48E-01 |  | 79,21 | 101,65 | 58,93 | 63,29 | 95,72 | 84,81 | 72,22 |
| Cox17 | cytochrome c oxidase assembly protein 17, copper chaperone |  | -28,17 | -0,42 | DOWN | 6,69E-01 |  | 234,02 | 193,63 | 138,71 | 190,89 | 148,04 | 239,90 | 210,73 |
| Rhot2 | ras homolog family member T2 |  | -22,20 | -0,64 | DOWN | 6,75E-01 |  | 147,62 | 82,29 | 94,74 | 140,47 | 102,10 | 41,24 | 85,24 |
| Mrpl15 | mitochondrial ribosomal protein L15 |  | 31,35 | 1,14 | UP | 6,95E-01 |  | 190,82 | 275,92 | 348,59 | 269,10 | 283,96 | 191,10 | 230,86 |
| Ndufs2 | NADH:ubiquinone oxidoreductase core subunit S2 |  | 37,32 | 1,12 | UP | 7,10E-01 |  | 370,84 | 203,31 | 303,71 | 337,53 | 266,09 | 363,62 | 351,03 |
| Hadhb | hydroxyacyl-CoA dehydrogenase trifunctional multienzyme complex subunit beta |  | 25,56 | 1,09 | UP | 7,21E-01 |  | 291,63 | 242,03 | 270,17 | 278,36 | 282,69 | 293,34 | 337,41 |
| Etfdh | electron transferring flavoprotein, dehydrogenase |  | -24,00 | -0,26 | DOWN | 7,25E-01 |  | 262,83 | 285,60 | 220,76 | 258,29 | 290,98 | 260,23 | 220,80 |
| Sdhc | succinate dehydrogenase complex, subunit C, integral membrane protein |  | 37,83 | 1,11 | UP | 7,25E-01 |  | 388,84 | 266,24 | 274,25 | 346,79 | 392,44 | 431,58 | 381,81 |
| Cox10 | heme A:farnesyltransferase cytochrome c oxidase assembly factor 10 |  | 11,39 | 1,14 | UP | 7,34E-01 |  | 79,21 | 77,45 | 76,61 | 108,57 | 104,01 | 83,06 | 76,36 |
| Mrpl11 | mitochondrial ribosomal protein L11 |  | 12,02 | 1,13 | UP | 7,40E-01 |  | 90,01 | 111,34 | 106,98 | 87,47 | 124,43 | 117,92 | 126,68 |
| Ndufb7 | NADH:ubiquinone oxidoreductase subunit B7 |  | 18,85 | 1,10 | UP | 7,50E-01 |  | 165,62 | 232,35 | 189,93 | 183,69 | 260,35 | 217,82 | 237,37 |
| Phb2 | prohibitin 2 |  | 65,09 | 1,09 | UP | 7,83E-01 |  | 896,49 | 566,36 | 703,07 | 1063,53 | 717,88 | 689,49 | 808,60 |
| Pdp1 | pyruvate dehydrogenase phosphatase catalytic subunit 1 |  | -14,30 | -0,30 | DOWN | 7,85E-01 |  | 136,81 | 150,06 | 106,07 | 184,20 | 117,41 | 121,98 | 116,02 |
| Acaa2 | acetyl-CoA acyltransferase 2 |  | 14,76 | 1,11 | UP | 7,97E-01 |  | 144,02 | 91,97 | 108,79 | 146,13 | 120,60 | 187,04 | 101,22 |
| Ndufs1 | NADH:ubiquinone oxidoreductase core subunit S1 |  | 25,12 | 1,07 | UP | 7,97E-01 |  | 320,43 | 498,59 | 417,94 | 506,81 | 421,79 | 420,55 | 406,08 |
| Ndufab1 | NADH:ubiquinone oxidoreductase subunit AB1 |  | 20,07 | 1,08 | UP | 8,45E-01 |  | 237,62 | 324,33 | 232,54 | 250,58 | 393,72 | 385,11 | 243,29 |
| Isca1 | iron-sulfur cluster assembly 1 |  | -14,05 | -0,17 | DOWN | 8,49E-01 |  | 198,02 | 261,40 | 197,64 | 199,64 | 242,48 | 252,10 | 186,46 |
| Ndufa4 | Ndufa4, mitochondrial complex associated |  | 21,52 | 1,09 | UP | 8,51E-01 |  | 277,23 | 154,90 | 175,88 | 383,84 | 276,30 | 171,36 | 180,54 |
| Mrps30 | mitochondrial ribosomal protein S30 |  | 5,28 | 1,02 | UP | 9,41E-01 |  | 198,02 | 164,58 | 170,89 | 177,00 | 224,62 | 189,94 | 170,48 |
| Mrps11 | mitochondrial ribosomal protein S11 |  | -1,13 | -0,12 | DOWN | 9,47E-01 |  | 46,80 | 29,04 | 32,18 | 55,57 | 27,44 | 25,56 | 43,21 |
| Dlst | dihydrolipoamide S-succinyltransferase |  | -6,69 | -0,06 | DOWN | 9,58E-01 |  | 421,24 | 334,01 | 542,60 | 299,97 | 417,33 | 317,73 | 277,03 |
| Suclg1 | succinate-CoA ligase, GDP-forming, alpha subunit |  | -0,30 | -0,02 | DOWN | 9,75E-01 |  | 309,63 | 242,03 | 282,86 | 281,45 | 279,49 | 283,46 | 250,39 |
