# Supplementary material for: Targeting metabolic vulnerabilities: REV-ERB agonist SR9009 potentiates sorafenib efficacy in liver cancer
Source: Cell Death Discov. 2026 Jan 19;12:86. doi: 10.1038/s41420-026-02940-3 (PMC12877144; doi:10.1038/s41420-026-02940-3)
Supplement: Supplementary file 3 — Supplementary Table 2 [file 41420_2026_2940_MOESM3_ESM.docx]

**SupplementaryTable 2.** Genes induced during early phases of sorafenib exposure in Hep55.1C cells and further upregulated upon acquisition of sorafenib resistance

| **Gene Symbol** | **GeneName** | **Description** | **Chromosome Number** | **Chromosome Strand** | **Cytoband** | **EnsemblID** | **Genbank Accession** | **RefSeq Accession** | **UniGeneID** |
| --- | --- | --- | --- | --- | --- | --- | --- | --- | --- |
| Aars | alanyl-tRNA synthetase | alanyl-tRNA synthetase [Source:MGI Symbol;Acc:MGI:2384560] [ENSMUST00000034441] | chr8 | + | mm\|8qE1 | ENSMUST00000034441 | BC033273 |  | Mm.24174 |
| Aarsd1 | alanyl-tRNA synthetase domain containing 1 | Mus musculus alanyl-tRNA synthetase domain containing 1 (Aarsd1), mRNA [NM_144829] | chr11 | - | mm\|11qD | ENSMUST00000107257 | NM_144829 | NM_144829 | Mm.146283 |
| Abce1 | ATP-binding cassette, sub-family E (OABP), member 1 | Mus musculus ATP-binding cassette, sub-family E (OABP), member 1 (Abce1), mRNA [NM_015751] | chr8 | - | mm\|8qC2 | ENSMUST00000080536 | NM_015751 | NM_015751 | Mm.5831 |
| Abcf2 | ATP-binding cassette, sub-family F (GCN20), member 2 | Mus musculus ATP-binding cassette, sub-family F (GCN20), member 2 (Abcf2), transcript variant 1, mRNA [NM_013853] | chr5 | - | mm\|5qA3 | ENSMUST00000030795 | NM_013853 | NM_013853 | Mm.21629 |
| Abhd12 | abhydrolase domain containing 12 | Mus musculus abhydrolase domain containing 12 (Abhd12), mRNA [NM_024465] | chr2 | - | mm\|2qG3 | ENSMUST00000145826 | NM_024465 | NM_024465 | Mm.112632 |
| Ablim1 | actin-binding LIM protein 1 | Mus musculus actin-binding LIM protein 1 (Ablim1), transcript variant 1, mRNA [NM_178688] | chr19 | - | mm\|19qD2 | ENSMUST00000099294 | NM_178688 | NM_178688 | Mm.217161 |
| Acot7 | acyl-CoA thioesterase 7 | Mus musculus acyl-CoA thioesterase 7 (Acot7), transcript variant 2, mRNA [NM_133348] | chr4 | + | mm\|4qE2 | ENSMUST00000030779 | NM_133348 | NM_133348 | Mm.296191 |
| Acox2 | acyl-Coenzyme A oxidase 2, branched chain | Mus musculus acyl-Coenzyme A oxidase 2, branched chain (Acox2), transcript variant 1, mRNA [NM_053115] | chr14 | - | mm\|14qA1 | ENSMUST00000164598 | NM_053115 | NM_053115 | Mm.28700 |
| Acp1 | acid phosphatase 1, soluble | Mus musculus acid phosphatase 1, soluble (Acp1), transcript variant 1, mRNA [NM_001110239] | chr12 | - | mm\|12qA2 | ENSMUST00000074038 | NM_001110239 | NM_001110239 | Mm.359831 |
| Actb | actin, beta | Mus musculus actin, beta (Actb), mRNA [NM_007393] | chr5 | - | mm\|5qG2 | ENSMUST00000100497 | NM_007393 | NM_007393 | Mm.391967 |
| Actr6 | ARP6 actin-related protein 6 | Mus musculus ARP6 actin-related protein 6 (Actr6), mRNA [NM_025914] | chr10 | - | mm\|10qC2 | ENSMUST00000020109 | NM_025914 | NM_025914 | Mm.335292 |
| Acyp1 | acylphosphatase 1, erythrocyte (common) type | Mus musculus acylphosphatase 1, erythrocyte (common) type (Acyp1), mRNA [NM_025421] | chr12 | - | mm\|12qD2 | ENSMUST00000117138 | NM_025421 | NM_025421 | Mm.311985 |
| Adh5 | alcohol dehydrogenase 5 (class III), chi polypeptide | Mus musculus alcohol dehydrogenase 5 (class III), chi polypeptide (Adh5), transcript variant 1, mRNA [NM_007410] | chr3 | + | mm\|3qG3 | ENSMUST00000005964 | NM_007410 | NM_007410 | Mm.3874 |
| Adh7 | alcohol dehydrogenase 7 (class IV), mu or sigma polypeptide | Mus musculus alcohol dehydrogenase 7 (class IV), mu or sigma polypeptide (Adh7), mRNA [NM_009626] | chr3 | + | mm\|3qG3 | ENSMUST00000090171 | NM_009626 | NM_009626 | Mm.8473 |
| Adnp | activity-dependent neuroprotective protein | Mus musculus activity-dependent neuroprotective protein (Adnp), transcript variant 2, mRNA [NM_009628] | chr2 | - | mm\|2qH3 | ENSMUST00000138667 | NM_009628 | NM_009628 | Mm.201322 |
| Aen | apoptosis enhancing nuclease | Mus musculus apoptosis enhancing nuclease (Aen), transcript variant 1, mRNA [NM_026531] | chr7 | + | mm\|7qD3 | ENSMUST00000107425 | NM_026531 | NM_026531 | Mm.34109 |
| Afp | alpha fetoprotein | Mus musculus alpha fetoprotein (Afp), mRNA [NM_007423] | chr5 | + | mm\|5qE1 | ENSMUST00000202209 | NM_007423 | NM_007423 | Mm.358570 |
| Agps | alkylglycerone phosphate synthase | Mus musculus alkylglycerone phosphate synthase (Agps), mRNA [NM_172666] | chr2 | + | mm\|2qC3 | ENSMUST00000047232 | NM_172666 | NM_172666 | Mm.31227 |
| Ahcy | S-adenosylhomocysteine hydrolase |  | chr2 | - | mm\|2qH1 |  | BC086781 |  | Mm.371964 |
| AI661453 | expressed sequence AI661453 | Mus musculus expressed sequence AI661453 (AI661453), mRNA [NM_145489] | chr17 | + | mm\|17qC | ENSMUST00000037701 | NM_145489 | NM_145489 | Mm.218444 |
| Ajuba | ajuba LIM protein | Mus musculus ajuba LIM protein (Ajuba), mRNA [NM_010590] | chr14 | - | mm\|14qC3 | ENSMUST00000054487 | NM_010590 | NM_010590 | Mm.100253 |
| Akirin2 | akirin 2 | Mus musculus akirin 2 (Akirin2), mRNA [NM_001007589] | chr4 | + | mm\|4qA5 | ENSMUST00000084299 | NM_001007589 | NM_001007589 | Mm.389532 |
| Akna | AT-hook transcription factor | Q8R114_MOUSE (Q8R114) AI597013 protein (Fragment), complete [TC1611432] | chr4 | - | mm\|4qC1 |  | BC036324 |  | Mm.387725 |
| Aldh18a1 | aldehyde dehydrogenase 18 family, member A1 | Mus musculus aldehyde dehydrogenase 18 family, member A1 (Aldh18a1), transcript variant 1, mRNA [NM_019698] | chr19 | - | mm\|19qC3 | ENSMUST00000025979 | NM_019698 | NM_019698 | Mm.233117 |
| Alg13 | asparagine-linked glycosylation 13 | Mus musculus asparagine-linked glycosylation 13 (Alg13), transcript variant 2, non-coding RNA [NR_037145] | chrX | + | mm\|XqF2 | ENSMUST00000197316 | NR_037145 | NR_037145 | Mm.249084 |
| Alg3 | asparagine-linked glycosylation 3 (alpha-1,3-mannosyltransferase) | Mus musculus asparagine-linked glycosylation 3 (alpha-1,3-mannosyltransferase) (Alg3), mRNA [NM_145939] | chr16 | - | mm\|16qB1 | ENSMUST00000123774 | NM_145939 | NM_145939 | Mm.24950 |
| Amfr | autocrine motility factor receptor | Mus musculus autocrine motility factor receptor (Amfr), mRNA [NM_011787] | chr8 | - | mm\|8qC5 | ENSMUST00000053766 | NM_011787 | NM_011787 | Mm.34641 |
| Anapc1 | anaphase promoting complex subunit 1 | anaphase promoting complex subunit 1 [Source:MGI Symbol;Acc:MGI:103097] [ENSMUST00000110332] | chr2 | - | mm\|2qF1 | ENSMUST00000110332 | BC057201 |  | Mm.277408 |
| Angptl8 | angiopoietin-like 8 | Mus musculus angiopoietin-like 8 (Angptl8), mRNA [NM_001080940] | chr9 | + | mm\|9qA3 | ENSMUST00000058777 | NM_001080940 | NM_001080940 | Mm.440264 |
| Ankra2 | ankyrin repeat, family A (RFXANK-like), 2 | Mus musculus ankyrin repeat, family A (RFXANK-like), 2 (Ankra2), transcript variant 1, mRNA [NM_001271388] | chr13 | + | mm\|13qD1 | ENSMUST00000022164 | NM_001271388 | NM_001271388 | Mm.209642 |
| Ankrd13d | ankyrin repeat domain 13 family, member D | Mus musculus ankyrin repeat domain 13 family, member D (Ankrd13d), mRNA [NM_026720] | chr19 | - | mm\|19qA | ENSMUST00000163858 | NM_026720 | NM_026720 | Mm.23642 |
| Antxr2 | anthrax toxin receptor 2 | Mus musculus anthrax toxin receptor 2 (Antxr2), mRNA [NM_133738] | chr5 | - | mm\|5qE3 | ENSMUST00000031281 | NM_133738 | NM_133738 | Mm.24842 |
| Anxa2 | annexin A2 | Mus musculus annexin A2 (Anxa2), mRNA [NM_007585] | chr9 | + | mm\|9qC | ENSMUST00000034756 | NM_007585 | NM_007585 | Mm.238343 |
| Ap1g1 | adaptor protein complex AP-1, gamma 1 subunit | Mus musculus adaptor protein complex AP-1, gamma 1 subunit (Ap1g1), transcript variant 1, mRNA [NM_009677] | chr8 | + | mm\|8qD3 | ENSMUST00000034171 | NM_009677 | NM_009677 | Mm.37210 |
| Ap2a2 | adaptor-related protein complex 2, alpha 2 subunit | Mus musculus adaptor-related protein complex 2, alpha 2 subunit (Ap2a2), mRNA [NM_007459] | chr7 | + | mm\|7qF5 | ENSMUST00000003038 | NM_007459 | NM_007459 | Mm.253090 |
| Apoo | apolipoprotein O | Mus musculus apolipoprotein O (Apoo), transcript variant 2, mRNA [NM_001199337] | chrX | + | mm\|XqC3 | ENSMUST00000113897 | NM_001199337 | NM_001199337 | Mm.379156 |
| Aprt | adenine phosphoribosyl transferase | Mus musculus adenine phosphoribosyl transferase (Aprt), mRNA [NM_009698] | chr8 | - | mm\|8qE1 | ENSMUST00000006764 | NM_009698 | NM_009698 | Mm.1786 |
| Arglu1 | arginine and glutamate rich 1 | Mus musculus arginine and glutamate rich 1 (Arglu1), mRNA [NM_176849] | chr8 | - | mm\|8qA1.1 | ENSMUST00000208252 | NM_176849 | NM_176849 | Mm.251655 |
| Arhgdia | Rho GDP dissociation inhibitor (GDI) alpha | Mus musculus Rho GDP dissociation inhibitor (GDI) alpha (Arhgdia), mRNA [NM_133796] | chr11 | - | mm\|11qE2 | ENSMUST00000067936 | NM_133796 | NM_133796 | Mm.30016 |
| Arhgef2 | rho/rac guanine nucleotide exchange factor (GEF) 2 | Mus musculus rho/rac guanine nucleotide exchange factor (GEF) 2 (Arhgef2), transcript variant 2, mRNA [NM_001198911] | chr3 | + | mm\|3qF1 | ENSMUST00000170653 | NM_001198911 | NM_001198911 | Mm.239329 |
| Arih1 | ariadne RBR E3 ubiquitin protein ligase 1 | Mus musculus ariadne RBR E3 ubiquitin protein ligase 1 (Arih1), mRNA [NM_019927] | chr9 | - | mm\|9qB | ENSMUST00000171975 | NM_019927 | NM_019927 | Mm.305925 |
| Arl14ep | ADP-ribosylation factor-like 14 effector protein | ADP-ribosylation factor-like 14 effector protein [Source:MGI Symbol;Acc:MGI:1926020] [ENSMUST00000124598] | chr2 | - | mm\|2qE3 | ENSMUST00000124598 | AK165082 |  | Mm.381231 |
| Arl6ip4 | ADP-ribosylation factor-like 6 interacting protein 4 | Mus musculus ADP-ribosylation factor-like 6 interacting protein 4 (Arl6ip4), mRNA [NM_144509] | chr5 | + | mm\|5qF | ENSMUST00000145667 | NM_144509 | NM_144509 | Mm.29430 |
| Arrdc3 | arrestin domain containing 3 | Mus musculus arrestin domain containing 3 (Arrdc3), mRNA [NM_001042591] | chr13 | + | mm\|13qC3 | ENSMUST00000099356 | NM_001042591 | NM_001042591 | Mm.423137 |
| Asah2 | N-acylsphingosine amidohydrolase 2 | Mus musculus N-acylsphingosine amidohydrolase 2 (Asah2), mRNA [NM_018830] | chr19 | - | mm\|19qC1 | ENSMUST00000096119 | NM_018830 | NM_018830 | Mm.491229 |
| Ascc2 | activating signal cointegrator 1 complex subunit 2 | Mus musculus activating signal cointegrator 1 complex subunit 2 (Ascc2), mRNA [NM_029291] | chr11 | + | mm\|11qA1 | ENSMUST00000109930 | NM_029291 | NM_029291 | Mm.439906 |
| Asns | asparagine synthetase | Mus musculus asparagine synthetase (Asns), mRNA [NM_012055] | chr6 | - | mm\|6qA1 | ENSMUST00000115542 | NM_012055 | NM_012055 | Mm.2942 |
| Atad1 | ATPase family, AAA domain containing 1 | Mus musculus ATPase family, AAA domain containing 1 (Atad1), mRNA [NM_026487] | chr19 | - | mm\|19qC1 | ENSMUST00000070210 | NM_026487 | NM_026487 | Mm.27123 |
| Atf1 | activating transcription factor 1 | Mus musculus activating transcription factor 1 (Atf1), mRNA [NM_007497] | chrX | - | mm\|XqA5 | ENSMUST00000023769 | NM_007497 | NM_007497 | Mm.676 |
| Atf3 | activating transcription factor 3 | Mus musculus activating transcription factor 3 (Atf3), mRNA [NM_007498] | chr1 | - | mm\|1qH6 | ENSMUST00000027941 | NM_007498 | NM_007498 | Mm.2706 |
| Atf4 | activating transcription factor 4 | Mus musculus activating transcription factor 4 (Atf4), transcript variant 1, mRNA [NM_009716] | chr15 | + | mm\|15qE1 | ENSMUST00000109605 | NM_009716 | NM_009716 | Mm.641 |
| Atf6b | activating transcription factor 6 beta | Mus musculus activating transcription factor 6 beta (Atf6b), mRNA [NM_017406] | chr17 | + | mm\|17qB1 | ENSMUST00000173984 | NM_017406 | NM_017406 | Mm.4068 |
| Atg101 | autophagy related 101 | Mus musculus autophagy related 101 (Atg101), mRNA [NM_026566] | chr15 | + | mm\|15qF2 | ENSMUST00000048393 | NM_026566 | NM_026566 | Mm.482360 |
| Atg3 | autophagy related 3 | Mus musculus autophagy related 3 (Atg3), mRNA [NM_026402] | chr16 | + | mm\|16qB5 | ENSMUST00000150390 | NM_026402 | NM_026402 | Mm.41775 |
| Atp5b | ATP synthase, H+ transporting mitochondrial F1 complex, beta subunit | Mus musculus ATP synthase, H+ transporting mitochondrial F1 complex, beta subunit (Atp5b), mRNA [NM_016774] | chr10 | + | mm\|10qD3 | ENSMUST00000125992 | NM_016774 | NM_016774 | Mm.238973 |
| Atp5e | ATP synthase, H+ transporting, mitochondrial F1 complex, epsilon subunit | Mus musculus ATP synthase, H+ transporting, mitochondrial F1 complex, epsilon subunit (Atp5e), mRNA [NM_025983] | chr2 | - | mm\|2qH4 | ENSMUST00000149191 | NM_025983 | NM_025983 | Mm.20841 |
| Atp5pb |  |  |  |  |  |  |  |  |  |
| Azin1 | antizyme inhibitor 1 | Mus musculus antizyme inhibitor 1 (Azin1), transcript variant 2, mRNA [NM_018745] | chr15 | - | mm\|15qB3.1 | ENSMUST00000110328 | NM_018745 | NM_018745 | Mm.250214 |
| Babam2 | BRISC and BRCA1 A complex member 2 | brain and reproductive organ-expressed protein [Source:MGI Symbol;Acc:MGI:1333875] [ENSMUST00000201352] | chr5 | + | mm\|5qB1 | ENSMUST00000201352 | XM_011240687 | XM_011240687 | Mm.482126 |
| Bbs4 | Bardet-Biedl syndrome 4 (human) | Mus musculus Bardet-Biedl syndrome 4 (human) (Bbs4), mRNA [NM_175325] | chr9 | - | mm\|9qB | ENSMUST00000026265 | NM_175325 | NM_175325 | Mm.200714 |
| Bnip2 | BCL2/adenovirus E1B interacting protein 2 | Mus musculus BCL2/adenovirus E1B interacting protein 2 (Bnip2), transcript variant alpha, mRNA [NM_001008238] | chr9 | + | mm\|9qD | ENSMUST00000212900 | NM_001008238 | NM_001008238 | Mm.159777 |
| Brms1 | breast cancer metastasis-suppressor 1 | Mus musculus breast cancer metastasis-suppressor 1 (Brms1), mRNA [NM_134155] | chr19 | + | mm\|19qA | ENSMUST00000116567 | NM_134155 | NM_134155 | Mm.29628 |
| Btf3-ps1 |  |  |  |  |  |  |  |  |  |
| C1d | C1D nuclear receptor co-repressor | Mus musculus C1D nuclear receptor co-repressor (C1d), transcript variant 2, mRNA [NM_001330649] | chr11 | + | mm\|11qA2 | ENSMUST00000000594 | NM_001330649 | NM_001330649 | Mm.287982 |
| C1qbp | complement component 1, q subcomponent binding protein | Mus musculus complement component 1, q subcomponent binding protein (C1qbp), mRNA [NM_007573] | chr11 | - | mm\|11qB4 | ENSMUST00000155371 | NM_007573 | NM_007573 | Mm.30049 |
| Cacybp | calcyclin binding protein | Mus musculus calcyclin binding protein (Cacybp), mRNA [NM_009786] | chr1 | - | mm\|1qH2.1 | ENSMUST00000014370 | NM_009786 | NM_009786 | Mm.10702 |
| Cad | carbamoyl-phosphate synthetase 2, aspartate transcarbamylase, and dihydroorotase | Mus musculus carbamoyl-phosphate synthetase 2, aspartate transcarbamylase, and dihydroorotase (Cad), transcript variant 2, mRNA [NM_001289522] | chr5 | + | mm\|5qB1 | ENSMUST00000201838 | NM_001289522 | NM_001289522 | Mm.305535 |
| Calm1 | calmodulin 1 | Mus musculus calmodulin 1 (Calm1), transcript variant 2, mRNA [NM_009790] | chr12 | + | mm\|12qE | ENSMUST00000110082 | NM_009790 | NM_009790 | Mm.285993 |
| Calm3 | calmodulin 3 | Mus musculus calmodulin 3 (Calm3), mRNA [NM_007590] | chr7 | - | mm\|7qA2 | ENSMUST00000019514 | NM_007590 | NM_007590 | Mm.288630 |
| Calr | calreticulin | Mus musculus calreticulin (Calr), mRNA [NM_007591] | chr8 | - | mm\|8qC3 | ENSMUST00000003912 | NM_007591 | NM_007591 | Mm.1971 |
| Calu | calumenin | Mus musculus calumenin (Calu), transcript variant 1, mRNA [NM_007594] | chr6 | + | mm\|6qA3.3 | ENSMUST00000090481 | NM_007594 | NM_007594 | Mm.392075 |
| Cars | cysteinyl-tRNA synthetase | Mus musculus cysteinyl-tRNA synthetase (Cars), transcript variant 1, mRNA [NM_013742] | chr7 | - | mm\|7qF5 | ENSMUST00000105909 | NM_013742 | NM_013742 | Mm.125659 |
| Cask | calcium/calmodulin-dependent serine protein kinase (MAGUK family) | calcium/calmodulin-dependent serine protein kinase (MAGUK family) [Source:MGI Symbol;Acc:MGI:1309489] [ENSMUST00000152358] | chrX | - | mm\|XqA1.1 | ENSMUST00000152358 |  |  |  |
| Ccdc107 | coiled-coil domain containing 107 | Mus musculus coiled-coil domain containing 107 (Ccdc107), mRNA [NM_001037913] | chr4 | + | mm\|4qB1 | ENSMUST00000107922 | NM_001037913 | NM_001037913 | Mm.289109 |
| Ccdc28a | coiled-coil domain containing 28A | Mus musculus coiled-coil domain containing 28A (Ccdc28a), transcript variant 2, mRNA [NM_001346751] | chr10 | - | mm\|10qA3 | ENSMUST00000052648 | NM_001346751 | NM_001346751 | Mm.296565 |
| Ccdc47 | coiled-coil domain containing 47 | Mus musculus coiled-coil domain containing 47 (Ccdc47), mRNA [NM_026009] | chr11 | - | mm\|11qA4 | ENSMUST00000002043 | NM_026009 | NM_026009 | Mm.22109 |
| Ccdc90b | coiled-coil domain containing 90B | Mus musculus coiled-coil domain containing 90B (Ccdc90b), transcript variant 1, mRNA [NM_025515] | chr7 | + | mm\|7qE1 | ENSMUST00000032842 | NM_025515 | NM_025515 | Mm.21173 |
| Ccnd1 | cyclin D1 | Mus musculus cyclin D1 (Ccnd1), mRNA [NM_007631] | chr7 | - | mm\|7qF5 | ENSMUST00000093962 | NM_007631 | NM_007631 | Mm.273049 |
| Ccnl1 | cyclin L1 | Mus musculus cyclin L1 (Ccnl1), mRNA [NM_019937] | chr3 | - | mm\|3qE1 | ENSMUST00000149729 | NM_019937 | NM_019937 | Mm.175612 |
| Ccp110 | centriolar coiled coil protein 110 | Mus musculus centriolar coiled coil protein 110 (Ccp110), mRNA [NM_182995] | chr7 | + | mm\|7qF2 | ENSMUST00000038650 | NM_182995 | NM_182995 | Mm.23279 |
| Cct4 | chaperonin containing Tcp1, subunit 4 (delta) | Mus musculus chaperonin containing Tcp1, subunit 4 (delta) (Cct4), mRNA [NM_009837] | chr11 | + | mm\|11qA3.2 | ENSMUST00000145912 | NM_009837 | NM_009837 | Mm.296985 |
| Cct5 | chaperonin containing Tcp1, subunit 5 (epsilon) | Mus musculus chaperonin containing Tcp1, subunit 5 (epsilon) (Cct5), transcript variant 1, mRNA [NM_007637] | chr15 | - | mm\|15qB2 | ENSMUST00000022842 | NM_007637 | NM_007637 | Mm.282158 |
| Cct6a | chaperonin containing Tcp1, subunit 6a (zeta) | Mus musculus chaperonin containing Tcp1, subunit 6a (zeta) (Cct6a), mRNA [NM_009838] | chr5 | + | mm\|5qG1.3 |  | NM_009838 | NM_009838 | Mm.360232 |
| Cct7 | chaperonin containing Tcp1, subunit 7 (eta) | Mus musculus chaperonin containing Tcp1, subunit 7 (eta) (Cct7), mRNA [NM_007638] | chr6 | + | mm\|6qC3 | ENSMUST00000204489 | NM_007638 | NM_007638 | Mm.289900 |
| Cct8 | chaperonin containing Tcp1, subunit 8 (theta) | Mus musculus chaperonin containing Tcp1, subunit 8 (theta) (Cct8), mRNA [NM_009840] | chr16 | - | mm\|16qC3.3 | ENSMUST00000176241 | NM_009840 | NM_009840 | Mm.328673 |
| Cd47 | CD47 antigen (Rh-related antigen, integrin-associated signal transducer) | CD47 antigen (Rh-related antigen, integrin-associated signal transducer) [Source:MGI Symbol;Acc:MGI:96617] [ENSMUST00000084838] | chr16 | + | mm\|16qB5 | ENSMUST00000084838 | AK164165 |  | Mm.31752 |
| Cdc42se2 | CDC42 small effector 2 | Mus musculus CDC42 small effector 2 (Cdc42se2), mRNA [NM_178626] | chr11 | - | mm\|11qB1.3 | ENSMUST00000064104 | NM_178626 | NM_178626 | Mm.29476 |
| Cdk11b | cyclin-dependent kinase 11B | Mus musculus cyclin-dependent kinase 11B (Cdk11b), transcript variant 1, mRNA [NM_007661] | chr4 | + | mm\|4qE2 | ENSMUST00000105600 | NM_007661 | NM_007661 | Mm.267410 |
| Cdk17 | cyclin-dependent kinase 17 | Mus musculus cyclin-dependent kinase 17 (Cdk17), mRNA [NM_146239] | chr10 | + | mm\|10qC2 | ENSMUST00000069965 | NM_146239 | NM_146239 | Mm.217404 |
| Cdpf1 | cysteine rich, DPF motif domain containing 1 | Mus musculus cysteine rich, DPF motif domain containing 1 (Cdpf1), transcript variant 2, mRNA [NM_197998] | chr15 | - | mm\|15qE2 | ENSMUST00000071876 | NM_197998 | NM_197998 | Mm.33706 |
| Cep83 | centrosomal protein 83 | Mus musculus centrosomal protein 83 (Cep83), mRNA [NM_029852] | chr10 | + | mm\|10qC2 | ENSMUST00000218076 | NM_029852 | NM_029852 | Mm.296971 |
| Cers2 | ceramide synthase 2 | Mus musculus ceramide synthase 2 (Cers2), transcript variant 1, mRNA [NM_029789] | chr3 | + | mm\|3qF2.1 | ENSMUST00000015858 | NM_029789 | NM_029789 | Mm.181009 |
| Cetn2 | centrin 2 | Mus musculus centrin 2 (Cetn2), mRNA [NM_019405] | chrX | - | mm\|XqA7.3 | ENSMUST00000114550 | NM_019405 | NM_019405 | Mm.24643 |
| Cfap36 | cilia and flagella associated protein 36 | Mus musculus cilia and flagella associated protein 36 (Cfap36), mRNA [NM_025740] | chr11 | - | mm\|11qA3.3 | ENSMUST00000020754 | NM_025740 | NM_025740 | Mm.128663 |
| Cfdp1 | craniofacial development protein 1 | Mus musculus craniofacial development protein 1 (Cfdp1), mRNA [NM_011801] | chr8 | - | mm\|8qE1 | ENSMUST00000034432 | NM_011801 | NM_011801 | Mm.279437 |
| Cfl1 | cofilin 1, non-muscle | Mus musculus cofilin 1, non-muscle (Cfl1), mRNA [NM_007687] | chr19 | + | mm\|19qA | ENSMUST00000065243 | NM_007687 | NM_007687 | Mm.329655 |
| Cgnl1 | cingulin-like 1 | Mus musculus cingulin-like 1 (Cgnl1), transcript variant 1, mRNA [NM_001304362] | chr9 | - | mm\|9qD | ENSMUST00000122065 | NM_001304362 | NM_001304362 | Mm.99961 |
| Chchd1 | coiled-coil-helix-coiled-coil-helix domain containing 1 | Mus musculus coiled-coil-helix-coiled-coil-helix domain containing 1 (Chchd1), mRNA [NM_025366] | chr14 | + | mm\|14qA3 | ENSMUST00000071215 | NM_025366 | NM_025366 | Mm.246996 |
| Chmp4b | charged multivesicular body protein 4B | Mus musculus charged multivesicular body protein 4B (Chmp4b), mRNA [NM_029362] | chr2 | + | mm\|2qH1 | ENSMUST00000151668 | NM_029362 | NM_029362 | Mm.262480 |
| Chuk | conserved helix-loop-helix ubiquitous kinase | Mus musculus conserved helix-loop-helix ubiquitous kinase (Chuk), transcript variant 1, mRNA [NM_007700] | chr19 | - | mm\|19qC3 | ENSMUST00000119591 | NM_007700 | NM_007700 | Mm.3996 |
| Cib1 | calcium and integrin binding 1 (calmyrin) | Mus musculus calcium and integrin binding 1 (calmyrin) (Cib1), transcript variant 1, mRNA [NM_011870] | chr7 | - | mm\|7qD3 | ENSMUST00000071457 | NM_011870 | NM_011870 | Mm.30217 |
| Clcc1 | chloride channel CLIC-like 1 | Mus musculus chloride channel CLIC-like 1 (Clcc1), transcript variant 3, mRNA [NM_001177771] | chr3 | + | mm\|3qF3 | ENSMUST00000029483 | NM_001177771 | NM_001177771 | Mm.214545 |
| Cldn12 | claudin 12 | Mus musculus claudin 12 (Cldn12), transcript variant 2, mRNA [NM_001193659] | chr5 | - | mm\|5qA1 | ENSMUST00000115446 | NM_001193659 | NM_001193659 | Mm.40132 |
| Cldnd1 | claudin domain containing 1 | Mus musculus claudin domain containing 1 (Cldnd1), transcript variant 1, mRNA [NM_171826] | chr16 | + | mm\|16qC1.2 | ENSMUST00000023426 | NM_171826 | NM_171826 | Mm.29482 |
| Cln6 | ceroid-lipofuscinosis, neuronal 6 | Mus musculus ceroid-lipofuscinosis, neuronal 6 (Cln6), mRNA [NM_001033175] | chr9 | + | mm\|9qB | ENSMUST00000034776 | NM_001033175 | NM_001033175 | Mm.283636 |
| Clock | circadian locomotor output cycles kaput | Mus musculus circadian locomotor output cycles kaput (Clock), transcript variant 1, mRNA [NM_007715] | chr5 | - | mm\|5qC3.3 | ENSMUST00000075159 | NM_007715 | NM_007715 | Mm.3552 |
| Cmc4 | C-x(9)-C motif containing 4 | C-x(9)-C motif containing 4 [Source:MGI Symbol;Acc:MGI:5637812] [ENSMUST00000120286] | chrX | - | mm\|XqA7.3 | ENSMUST00000120286 |  |  |  |
| Cmss1 | cms small ribosomal subunit 1 | Mus musculus cms small ribosomal subunit 1 (Cmss1), mRNA [NM_025599] | chr16 | - | mm\|16qC1.1 | ENSMUST00000114371 | NM_025599 | NM_025599 | Mm.425976 |
| Cnih1 | cornichon family AMPA receptor auxiliary protein 1 | Mus musculus cornichon family AMPA receptor auxiliary protein 1 (Cnih1), mRNA [NM_009919] | chr14 | - | mm\|14qC1 | ENSMUST00000146629 | NM_009919 | NM_009919 | Mm.3261 |
| Cnpy4 | canopy FGF signaling regulator 4 | canopy FGF signaling regulator 4 [Source:MGI Symbol;Acc:MGI:1913705] [ENSMUST00000110932] | chr5 | + | mm\|5qG2 | ENSMUST00000110932 |  |  |  |
| Coa3 | cytochrome C oxidase assembly factor 3 | Mus musculus cytochrome C oxidase assembly factor 3 (Coa3), mRNA [NM_026618] | chr11 | - | mm\|11qD | ENSMUST00000017332 | NM_026618 | NM_026618 | Mm.52 |
| Coa6 | cytochrome c oxidase assembly factor 6 | Mus musculus cytochrome c oxidase assembly factor 6 (Coa6), mRNA [NM_174987] | chr8 | + | mm\|8qE2 | ENSMUST00000211868 | NM_174987 | NM_174987 | Mm.31946 |
| Coq6 | coenzyme Q6 monooxygenase | Mus musculus coenzyme Q6 monooxygenase (Coq6), mRNA [NM_172582] | chr12 | + | mm\|12qD1 | ENSMUST00000150391 | NM_172582 | NM_172582 | Mm.280062 |
| Cox11 | cytochrome c oxidase assembly protein 11 | cytochrome c oxidase assembly protein 11 [Source:MGI Symbol;Acc:MGI:1917052] [ENSMUST00000099960] | chr11 | + | mm\|11qD | ENSMUST00000099960 |  |  |  |
| Cox5a | cytochrome c oxidase subunit Va | Mus musculus cytochrome c oxidase subunit Va (Cox5a), mRNA [NM_007747] | chr9 | + | mm\|9qB | ENSMUST00000213678 | NM_007747 | NM_007747 | Mm.273403 |
| Cox5b | cytochrome c oxidase subunit Vb | Mus musculus cytochrome c oxidase subunit Vb (Cox5b), mRNA [NM_009942] | chr1 | + | mm\|1qB | ENSMUST00000081180 | NM_009942 | NM_009942 | Mm.180182 |
| Cox6a2 | cytochrome c oxidase subunit VIa polypeptide 2 | Mus musculus cytochrome c oxidase subunit VIa polypeptide 2 (Cox6a2), mRNA [NM_009943] | chr7 | - | mm\|7qF3 | ENSMUST00000033049 | NM_009943 | NM_009943 | Mm.43824 |
| Cox7a2 | cytochrome c oxidase subunit VIIa 2 | Mus musculus cytochrome c oxidase subunit VIIa 2 (Cox7a2), mRNA [NM_009945] | chr9 | - | mm\|9qE1 | ENSMUST00000215933 | NM_009945 | NM_009945 | Mm.152627 |
| Cpox | coproporphyrinogen oxidase | Mus musculus coproporphyrinogen oxidase (Cpox), mRNA [NM_007757] | chr16 | + | mm\|16qC1.2 | ENSMUST00000060077 | NM_007757 | NM_007757 | Mm.291519 |
| Cpsf3 | cleavage and polyadenylation specificity factor 3 | Mus musculus cleavage and polyadenylation specificity factor 3 (Cpsf3), mRNA [NM_018813] | chr12 | + | mm\|12qA1.2 | ENSMUST00000221042 | NM_018813 | NM_018813 | Mm.356778 |
| Cpsf4 | cleavage and polyadenylation specific factor 4 | Mus musculus cleavage and polyadenylation specific factor 4 (Cpsf4), transcript variant 3, mRNA [NM_178576] | chr5 | + | mm\|5qG2 | ENSMUST00000160762 | NM_178576 | NM_178576 | Mm.196884 |
| Crcp | calcitonin gene-related peptide-receptor component protein | Mus musculus calcitonin gene-related peptide-receptor component protein (Crcp), mRNA [NM_007761] | chr5 | + | mm\|5qG1.3 | ENSMUST00000026608 | NM_007761 | NM_007761 | Mm.18072 |
| Creld1 | cysteine-rich with EGF-like domains 1 | Mus musculus cysteine-rich with EGF-like domains 1 (Creld1), transcript variant 1, mRNA [NM_133930] | chr6 | + | mm\|6qE3 | ENSMUST00000129125 | NM_133930 | NM_133930 | Mm.41593 |
| Crls1 | cardiolipin synthase 1 | Mus musculus cardiolipin synthase 1 (Crls1), transcript variant 1, mRNA [NM_001024385] | chr2 | + | mm\|2qF2 | ENSMUST00000028835 | NM_001024385 | NM_001024385 | Mm.357342 |
| Cry1 | cryptochrome 1 (photolyase-like) | Mus musculus cryptochrome 1 (photolyase-like) (Cry1), mRNA [NM_007771] | chr10 | - | mm\|10qC1 | ENSMUST00000020227 | NM_007771 | NM_007771 | Mm.26237 |
| Cs | citrate synthase | Mus musculus citrate synthase (Cs), mRNA [NM_026444] | chr10 | + | mm\|10qD3 | ENSMUST00000005826 | NM_026444 | NM_026444 | Mm.58836 |
| Cth | cystathionase (cystathionine gamma-lyase) | Mus musculus cystathionase (cystathionine gamma-lyase) (Cth), mRNA [NM_145953] | chr3 | - | mm\|3qH4 | ENSMUST00000129805 | NM_145953 | NM_145953 | Mm.28301 |
| Ctps | cytidine 5'-triphosphate synthase | Mus musculus cytidine 5'-triphosphate synthase (Ctps), mRNA [NM_016748] | chr4 | - | mm\|4qD2.2 | ENSMUST00000030381 | NM_016748 | NM_016748 | Mm.1815 |
| Cul2 | cullin 2 | Mus musculus cullin 2 (Cul2), mRNA [NM_029402] | chr18 | + | mm\|18qA1 |  | NM_029402 | NM_029402 | Mm.291707 |
| Cyb5r1 | cytochrome b5 reductase 1 | Mus musculus cytochrome b5 reductase 1 (Cyb5r1), mRNA [NM_028057] | chr1 | + | mm\|1qE4 | ENSMUST00000154237 | NM_028057 | NM_028057 | Mm.280230 |
| Cyc1 | cytochrome c-1 | Mus musculus cytochrome c-1 (Cyc1), mRNA [NM_025567] | chr15 | + | mm\|15qD3 | ENSMUST00000023210 | NM_025567 | NM_025567 | Mm.29196 |
| Cycs | cytochrome c, somatic | Mus musculus cytochrome c, somatic (Cycs), mRNA [NM_007808] | chr6 | - | mm\|6qB2.3 | ENSMUST00000073080 | NM_007808 | NM_007808 | Mm.35389 |
| Cyrib |  |  |  |  |  |  |  |  |  |
| Daam1 | dishevelled associated activator of morphogenesis 1 | Mus musculus dishevelled associated activator of morphogenesis 1 (Daam1), transcript variant 3, mRNA [NM_001286452] | chr12 | + | mm\|12qC3 |  | NM_001286452 | NM_001286452 | Mm.87417 |
| Dad1 | defender against cell death 1 | Mus musculus defender against cell death 1 (Dad1), transcript variant 2, mRNA [NM_010015] | chr14 | - | mm\|14qC2 | ENSMUST00000128231 | NM_010015 | NM_010015 |  |
| Dctn5 | dynactin 5 | Mus musculus dynactin 5 (Dctn5), mRNA [NM_021608] | chr7 | + | mm\|7qF3 | ENSMUST00000123602 | NM_021608 | NM_021608 | Mm.478285 |
| Dcun1d5 | DCN1, defective in cullin neddylation 1, domain containing 5 (S. cerevisiae) | Mus musculus DCN1, defective in cullin neddylation 1, domain containing 5 (S. cerevisiae) (Dcun1d5), mRNA [NM_029775] | chr9 | + | mm\|9qA1 | ENSMUST00000034499 | NM_029775 | NM_029775 | Mm.27293 |
| Ddah2 | dimethylarginine dimethylaminohydrolase 2 | Mus musculus dimethylarginine dimethylaminohydrolase 2 (Ddah2), transcript variant 2, mRNA [NM_016765] | chr17 | + | mm\|17qB1 | ENSMUST00000007255 | NM_016765 | NM_016765 | Mm.1457 |
| Ddias | DNA damage-induced apoptosis suppressor | Mus musculus DNA damage-induced apoptosis suppressor (Ddias), mRNA [NM_001080995] | chr7 | - | mm\|7qE1 | ENSMUST00000032877 | NM_001080995 | NM_001080995 | Mm.26468 |
| Ddost | dolichyl-di-phosphooligosaccharide-protein glycotransferase | Mus musculus dolichyl-di-phosphooligosaccharide-protein glycotransferase (Ddost), mRNA [NM_007838] | chr4 | + | mm\|4qD3 | ENSMUST00000030538 | NM_007838 | NM_007838 | Mm.7236 |
| Ddrgk1 | DDRGK domain containing 1 | Mus musculus DDRGK domain containing 1 (Ddrgk1), mRNA [NM_029832] | chr2 | - | mm\|2qF1 | ENSMUST00000124559 | NM_029832 | NM_029832 | Mm.440063 |
| Ddt | D-dopachrome tautomerase | Mus musculus D-dopachrome tautomerase (Ddt), mRNA [NM_010027] | chr10 | - | mm\|10qC1 | ENSMUST00000001716 | NM_010027 | NM_010027 | Mm.298947 |
| Ddx20 | DEAD (Asp-Glu-Ala-Asp) box polypeptide 20 | Mus musculus DEAD (Asp-Glu-Ala-Asp) box polypeptide 20 (Ddx20), mRNA [NM_017397] | chr3 | - | mm\|3qF2.2 | ENSMUST00000200078 | NM_017397 | NM_017397 | Mm.272826 |
| Ddx24 | DEAD (Asp-Glu-Ala-Asp) box polypeptide 24 | Mus musculus DEAD (Asp-Glu-Ala-Asp) box polypeptide 24 (Ddx24), transcript variant 1, mRNA [NM_001159502] | chr12 | - | mm\|12qE | ENSMUST00000110001 | NM_001159502 | NM_001159502 | Mm.3935 |
| Dennd4a | DENN/MADD domain containing 4A |  | chr9 | + | mm\|9qC |  | AK158840 |  |  |
| Desi2 | desumoylating isopeptidase 2 | desumoylating isopeptidase 2 [Source:MGI Symbol;Acc:MGI:1926075] [ENSMUST00000069568] | chr1 | + | mm\|1qH4 | ENSMUST00000069568 | AK078283 |  | Mm.39442 |
| Dgat1 | diacylglycerol O-acyltransferase 1 | Mus musculus diacylglycerol O-acyltransferase 1 (Dgat1), mRNA [NM_010046] | chr15 | - | mm\|15qD3 | ENSMUST00000162354 | NM_010046 | NM_010046 | Mm.22633 |
| Dhh | desert hedgehog | Mus musculus desert hedgehog (Dhh), mRNA [NM_007857] | chr15 | - | mm\|15qF1 | ENSMUST00000023737 | NM_007857 | NM_007857 | Mm.384073 |
| Dhps | deoxyhypusine synthase | Mus musculus deoxyhypusine synthase (Dhps), mRNA [NM_001039514] | chr8 | + | mm\|8qC3 | ENSMUST00000142210 | NM_001039514 | NM_001039514 | Mm.193086 |
| Dhrs7 | dehydrogenase/reductase (SDR family) member 7 | Mus musculus dehydrogenase/reductase (SDR family) member 7 (Dhrs7), mRNA [NM_025522] | chr12 | - | mm\|12qC3 | ENSMUST00000220821 | NM_025522 | NM_025522 | Mm.289653 |
| Dhx38 | DEAH (Asp-Glu-Ala-His) box polypeptide 38 | Mus musculus DEAH (Asp-Glu-Ala-His) box polypeptide 38 (Dhx38), mRNA [NM_178380] | chr8 | - | mm\|8qD3 | ENSMUST00000212667 | NM_178380 | NM_178380 | Mm.23705 |
| Dhx8 | DEAH (Asp-Glu-Ala-His) box polypeptide 8 | Mus musculus DEAH (Asp-Glu-Ala-His) box polypeptide 8 (Dhx8), mRNA [NM_144831] | chr11 | + | mm\|11qD | ENSMUST00000039152 | NM_144831 | NM_144831 | Mm.28186 |
| Dip2b | disco interacting protein 2 homolog B | Mus musculus disco interacting protein 2 homolog B (Dip2b), transcript variant 1, mRNA [NM_001159361] | chr15 | + | mm\|15qF1 | ENSMUST00000100203 | NM_001159361 | NM_001159361 | Mm.243658 |
| Dmac1 |  |  |  |  |  |  |  |  |  |
| Dmap1 | DNA methyltransferase 1-associated protein 1 | Mus musculus DNA methyltransferase 1-associated protein 1 (Dmap1), mRNA [NM_023178] | chr4 | - | mm\|4qD2.1 | ENSMUST00000129510 | NM_023178 | NM_023178 | Mm.29142 |
| Dnaja2 | DnaJ heat shock protein family (Hsp40) member A2 | Mus musculus DnaJ heat shock protein family (Hsp40) member A2 (Dnaja2), mRNA [NM_019794] | chr8 | - | mm\|8qC3 | ENSMUST00000034138 | NM_019794 | NM_019794 | Mm.475573 |
| Dnaja3 | DnaJ heat shock protein family (Hsp40) member A3 | Mus musculus DnaJ heat shock protein family (Hsp40) member A3 (Dnaja3), transcript variant 1, mRNA [NM_023646] | chr16 | + | mm\|16qA1 | ENSMUST00000060067 | NM_023646 | NM_023646 | Mm.248337 |
| Dnajb11 | DnaJ heat shock protein family (Hsp40) member B11 | Mus musculus DnaJ heat shock protein family (Hsp40) member B11 (Dnajb11), transcript variant 2, mRNA [NM_001190804] | chr16 | + | mm\|16qB1 | ENSMUST00000004574 | NM_001190804 | NM_001190804 | Mm.37516 |
| Dnajc2 | DnaJ heat shock protein family (Hsp40) member C2 | DnaJ heat shock protein family (Hsp40) member C2 [Source:MGI Symbol;Acc:MGI:99470] [ENSMUST00000115192] | chr5 | - | mm\|5qA3 | ENSMUST00000115192 | AK162409 |  | Mm.266312 |
| Dnajc8 | DnaJ heat shock protein family (Hsp40) member C8 | Mus musculus DnaJ heat shock protein family (Hsp40) member C8 (Dnajc8), mRNA [NM_172400] | chr4 | + | mm\|4qD2.3 | ENSMUST00000105937 | NM_172400 | NM_172400 | Mm.29685 |
| Dnajc9 | DnaJ heat shock protein family (Hsp40) member C9 | Mus musculus DnaJ heat shock protein family (Hsp40) member C9 (Dnajc9), mRNA [NM_134081] | chr14 | - | mm\|14qA3 | ENSMUST00000022345 | NM_134081 | NM_134081 | Mm.2871 |
| Dnal4 | dynein, axonemal, light chain 4 | Mus musculus dynein, axonemal, light chain 4 (Dnal4), mRNA [NM_017470] | chr15 | - | mm\|15qE1 | ENSMUST00000069877 | NM_017470 | NM_017470 | Mm.288159 |
| Dnttip2 | deoxynucleotidyltransferase, terminal, interacting protein 2 | Mus musculus deoxynucleotidyltransferase, terminal, interacting protein 2 (Dnttip2), mRNA [NM_153806] | chr3 | + | mm\|3qG1 | ENSMUST00000196338 | NM_153806 | NM_153806 | Mm.398647 |
| Dock5 | dedicator of cytokinesis 5 | Mus musculus dedicator of cytokinesis 5 (Dock5), mRNA [NM_177780] | chr14 | - | mm\|14qD1 | ENSMUST00000039135 | NM_177780 | NM_177780 | Mm.258155 |
| Dph1 | diphthamide biosynthesis 1 | Mus musculus diphthamide biosynthesis 1 (Dph1), mRNA [NM_144491] | chr11 | - | mm\|11qB5 | ENSMUST00000123489 | NM_144491 | NM_144491 | Mm.41496 |
| Dpm1 | dolichol-phosphate (beta-D) mannosyltransferase 1 | Mus musculus dolichol-phosphate (beta-D) mannosyltransferase 1 (Dpm1), transcript variant 1, mRNA [NM_010072] | chr2 | - | mm\|2qH3 | ENSMUST00000136582 | NM_010072 | NM_010072 | Mm.422657 |
| Dpm3 | dolichyl-phosphate mannosyltransferase polypeptide 3 | Mus musculus dolichyl-phosphate mannosyltransferase polypeptide 3 (Dpm3), mRNA [NM_026767] | chr3 | + | mm\|3qF1 | ENSMUST00000107462 | NM_026767 | NM_026767 | Mm.272927 |
| Drg2 | developmentally regulated GTP binding protein 2 | Mus musculus developmentally regulated GTP binding protein 2 (Drg2), mRNA [NM_021354] | chr11 | + | mm\|11qB2 | ENSMUST00000018568 | NM_021354 | NM_021354 | Mm.41803 |
| Dus1l | dihydrouridine synthase 1-like (S. cerevisiae) | Mus musculus dihydrouridine synthase 1-like (S. cerevisiae) (Dus1l), mRNA [NM_026824] | chr11 | - | mm\|11qE2 | ENSMUST00000146008 | NM_026824 | NM_026824 | Mm.180622 |
| Dxo | decapping exoribonuclease | Mus musculus decapping exoribonuclease (Dxo), transcript variant 1, mRNA [NM_033613] | chr17 | + | mm\|17qB1 | ENSMUST00000174684 | NM_033613 | NM_033613 | Mm.275309 |
| Eea1 | early endosome antigen 1 | Mus musculus early endosome antigen 1 (Eea1), mRNA [NM_001001932] | chr10 | + | mm\|10qC2 | ENSMUST00000053484 | NM_001001932 | NM_001001932 | Mm.490373 |
| Eif1b | eukaryotic translation initiation factor 1B | Mus musculus eukaryotic translation initiation factor 1B (Eif1b), mRNA [NM_026892] | chr9 | + | mm\|9qF4 | ENSMUST00000007139 | NM_026892 | NM_026892 | Mm.28753 |
| Eif2b1 | eukaryotic translation initiation factor 2B, subunit 1 (alpha) | Mus musculus eukaryotic translation initiation factor 2B, subunit 1 (alpha) (Eif2b1), mRNA [NM_145371] | chr5 | - | mm\|5qF | ENSMUST00000031334 | NM_145371 | NM_145371 | Mm.28839 |
| Eif2b5 | eukaryotic translation initiation factor 2B, subunit 5 epsilon | Mus musculus eukaryotic translation initiation factor 2B, subunit 5 epsilon (Eif2b5), mRNA [NM_172265] | chr16 | + | mm\|16qA3 | ENSMUST00000148714 | NM_172265 | NM_172265 | Mm.233855 |
| Eif2s1 | eukaryotic translation initiation factor 2, subunit 1 alpha | Mus musculus eukaryotic translation initiation factor 2, subunit 1 alpha (Eif2s1), mRNA [NM_026114] | chr12 | + | mm\|12qC3 | ENSMUST00000071230 | NM_026114 | NM_026114 | Mm.196220 |
| Eif2s2 | eukaryotic translation initiation factor 2, subunit 2 (beta) | Mus musculus eukaryotic translation initiation factor 2, subunit 2 (beta) (Eif2s2), mRNA [NM_026030] | chr2 | - | mm\|2qH1 | ENSMUST00000099173 | NM_026030 | NM_026030 | Mm.377134 |
| Eif2s3x | eukaryotic translation initiation factor 2, subunit 3, structural gene X-linked | Mus musculus eukaryotic translation initiation factor 2, subunit 3, structural gene X-linked (Eif2s3x), mRNA [NM_012010] | chrX | - | mm\|XqC3 | ENSMUST00000112099 | NM_012010 | NM_012010 | Mm.218851 |
| Eif3b | eukaryotic translation initiation factor 3, subunit B | Mus musculus eukaryotic translation initiation factor 3, subunit B (Eif3b), mRNA [NM_133916] | chr5 | + | mm\|5qG2 | ENSMUST00000199377 | NM_133916 | NM_133916 | Mm.21671 |
| Eif3c | eukaryotic translation initiation factor 3, subunit C | eukaryotic translation initiation factor 3, subunit C [Source:MGI Symbol;Acc:MGI:1926966] [ENSMUST00000032992] | chr7 | - | mm\|7qF3 | ENSMUST00000032992 |  |  |  |
| Eif3g | eukaryotic translation initiation factor 3, subunit G | Mus musculus eukaryotic translation initiation factor 3, subunit G (Eif3g), mRNA [NM_016876] | chr9 | - | mm\|9qA3 | ENSMUST00000004206 | NM_016876 | NM_016876 | Mm.427309 |
| Eif3j2 | eukaryotic translation initiation factor 3, subunit J2 | Mus musculus eukaryotic translation initiation factor 3, subunit J2 (Eif3j2), mRNA [NM_001256055] | chr2 | + | mm\|2qE5 | ENSMUST00000057110 | NM_001256055 | NM_001256055 | Mm.329403 |
| Eif4a1 | eukaryotic translation initiation factor 4A1 | Mus musculus eukaryotic translation initiation factor 4A1 (Eif4a1), transcript variant 1, mRNA [NM_144958] | chr11 | - | mm\|11qB3 | ENSMUST00000123995 | NM_144958 | NM_144958 | Mm.371557 |
| Eif4a2 | eukaryotic translation initiation factor 4A2 | Mus musculus eukaryotic translation initiation factor 4A2 (Eif4a2), transcript variant 3, mRNA [NM_001123038] | chr16 | + | mm\|16qB1 | ENSMUST00000115341 | NM_001123038 | NM_001123038 | Mm.260084 |
| Eif4a-ps4 |  |  |  |  |  |  |  |  |  |
| Eif4e | eukaryotic translation initiation factor 4E | Mus musculus eukaryotic translation initiation factor 4E (Eif4e), transcript variant 2, mRNA [NM_001313980] | chr3 | + | mm\|3qH1 | ENSMUST00000029803 | NM_001313980 | NM_001313980 | Mm.3941 |
| Eif4g1 | eukaryotic translation initiation factor 4, gamma 1 | Mus musculus eukaryotic translation initiation factor 4, gamma 1 (Eif4g1), transcript variant 1, mRNA [NM_145941] | chr16 | + | mm\|16qB1 | ENSMUST00000115460 | NM_145941 | NM_145941 | Mm.260256 |
| Eif5a | eukaryotic translation initiation factor 5A | Mus musculus eukaryotic translation initiation factor 5A (Eif5a), transcript variant 1, mRNA [NM_001166589] | chr11 | - | mm\|11qB3 | ENSMUST00000043419 | NM_001166589 | NM_001166589 | Mm.18026 |
| Elp3 | elongator acetyltransferase complex subunit 3 | Mus musculus elongator acetyltransferase complex subunit 3 (Elp3), transcript variant 2, mRNA [NM_028811] | chr14 | - | mm\|14qD1 | ENSMUST00000022609 | NM_028811 | NM_028811 | Mm.29719 |
| Emc1 | ER membrane protein complex subunit 1 | Mus musculus ER membrane protein complex subunit 1 (Emc1), transcript variant 1, mRNA [NM_146157] | chr4 | + | mm\|4qD3 | ENSMUST00000082262 | NM_146157 | NM_146157 | Mm.394288 |
| Emc6 | ER membrane protein complex subunit 6 | Mus musculus ER membrane protein complex subunit 6 (Emc6), transcript variant 2, mRNA [NM_025318] | chr11 | - | mm\|11qB4 | ENSMUST00000054952 | NM_025318 | NM_025318 | Mm.73608 |
| Emd | emerin | PREDICTED: Mus musculus emerin (Emd), transcript variant X1, mRNA [XM_011247521] | chrX | + | mm\|XqA7.3 |  | XM_011247521 | XM_011247521 | Mm.13886 |
| Eny2 | enhancer of yellow 2 homolog (Drosophila) | Mus musculus enhancer of yellow 2 homolog (Drosophila) (Eny2), mRNA [NM_175009] | chr15 | + | mm\|15qB3.2 | ENSMUST00000060652 | NM_175009 | NM_175009 | Mm.291828 |
| Eprs | glutamyl-prolyl-tRNA synthetase | Mus musculus glutamyl-prolyl-tRNA synthetase (Eprs), mRNA [NM_029735] | chr1 | + | mm\|1qH5 | ENSMUST00000046514 | NM_029735 | NM_029735 | Mm.154511 |
| Etf1 | eukaryotic translation termination factor 1 | Mus musculus eukaryotic translation termination factor 1 (Etf1), mRNA [NM_144866] | chr18 | - | mm\|18qB1 | ENSMUST00000025218 | NM_144866 | NM_144866 | Mm.329353 |
| Etv5 | ets variant 5 | Mus musculus ets variant 5 (Etv5), mRNA [NM_023794] | chr16 | - | mm\|16qB1 | ENSMUST00000079601 | NM_023794 | NM_023794 | Mm.155708 |
| Exosc1 | exosome component 1 | Mus musculus exosome component 1 (Exosc1), transcript variant 1, mRNA [NM_025644] | chr19 | - | mm\|19qC3 | ENSMUST00000075280 | NM_025644 | NM_025644 | Mm.289086 |
| Exosc4 | exosome component 4 | Mus musculus exosome component 4 (Exosc4), mRNA [NM_175399] | chr15 | + | mm\|15qD3 | ENSMUST00000059045 | NM_175399 | NM_175399 | Mm.322752 |
| F3 | coagulation factor III | Mus musculus coagulation factor III (F3), mRNA [NM_010171] | chr3 | + | mm\|3qG1 | ENSMUST00000029771 | NM_010171 | NM_010171 | Mm.273188 |
| Faf1 | Fas-associated factor 1 | Mus musculus Fas-associated factor 1 (Faf1), mRNA [NM_007983] | chr4 | + | mm\|4qC7 | ENSMUST00000102724 | NM_007983 | NM_007983 | Mm.318259 |
| Fahd2a | fumarylacetoacetate hydrolase domain containing 2A | Mus musculus fumarylacetoacetate hydrolase domain containing 2A (Fahd2a), mRNA [NM_029629] | chr2 | - | mm\|2qF1 | ENSMUST00000123327 | NM_029629 | NM_029629 | Mm.288676 |
| Fam107b | family with sequence similarity 107, member B | Mus musculus family with sequence similarity 107, member B (Fam107b), mRNA [NM_025626] | chr2 | + | mm\|2qA1 | ENSMUST00000027965 | NM_025626 | NM_025626 | Mm.277864 |
| Fam135a | family with sequence similarity 135, member A | Mus musculus family with sequence similarity 135, member A (Fam135a), mRNA [NM_026604] | chr1 | - | mm\|1qA5 | ENSMUST00000027337 | NM_026604 | NM_026604 | Mm.87130 |
| Fam151b | family with sequence similarity 151, member B | Mus musculus family with sequence similarity 151, member B (Fam151b), mRNA [NM_001163627] | chr13 | - | mm\|13qC3 | ENSMUST00000040106 | NM_001163627 | NM_001163627 | Mm.85935 |
| Fam174c |  |  |  |  |  |  |  |  |  |
| Fam89b | family with sequence similarity 89, member B | Mus musculus family with sequence similarity 89, member B (Fam89b), transcript variant 1, mRNA [NM_181452] | chr19 | - | mm\|19qA | ENSMUST00000116558 | NM_181452 | NM_181452 | Mm.391142 |
| Fbln2 | fibulin 2 | Mus musculus fibulin 2 (Fbln2), transcript variant 1, mRNA [NM_007992] | chr6 | + | mm\|6qD1 | ENSMUST00000137029 | NM_007992 | NM_007992 | Mm.249146 |
| Fbxl12 | F-box and leucine-rich repeat protein 12 | Mus musculus F-box and leucine-rich repeat protein 12 (Fbxl12), transcript variant 1, mRNA [NM_013911] | chr9 | - | mm\|9qA3 | ENSMUST00000086459 | NM_013911 | NM_013911 | Mm.489685 |
| Fbxl6 | F-box and leucine-rich repeat protein 6 | Mus musculus F-box and leucine-rich repeat protein 6 (Fbxl6), mRNA [NM_013909] | chr15 | - | mm\|15qD3 | ENSMUST00000023219 | NM_013909 | NM_013909 | Mm.275279 |
| Fbxo3 | F-box protein 3 | Mus musculus F-box protein 3 (Fbxo3), transcript variant 2, mRNA [NM_020593] | chr2 | + | mm\|2qE2 | ENSMUST00000102565 | NM_020593 | NM_020593 | Mm.143768 |
| Fbxw17 | F-box and WD-40 domain protein 17 | Mus musculus F-box and WD-40 domain protein 17 (Fbxw17), mRNA [NM_175401] | chr13 | + | mm\|13qA5 | ENSMUST00000176543 | NM_175401 | NM_175401 | Mm.18638 |
| Fhl2 | four and a half LIM domains 2 | Mus musculus four and a half LIM domains 2 (Fhl2), transcript variant 1, mRNA [NM_010212] | chr1 | - | mm\|1qB | ENSMUST00000008280 | NM_010212 | NM_010212 | Mm.6799 |
| Fkbp1a | FK506 binding protein 1a | Mus musculus FK506 binding protein 1a (Fkbp1a), transcript variant 4, mRNA [NM_001302078] | chr2 | + | mm\|2qG3 | ENSMUST00000144171 | NM_001302078 | NM_001302078 | Mm.278458 |
| Flnc | filamin C, gamma | Mus musculus filamin C, gamma (Flnc), transcript variant 1, mRNA [NM_001081185] | chr6 | + | mm\|6qA3.3 | ENSMUST00000065090 | NM_001081185 | NM_001081185 | Mm.39046 |
| Fosl1 | fos-like antigen 1 | Mus musculus fos-like antigen 1 (Fosl1), mRNA [NM_010235] | chr19 | + | mm\|19qA | ENSMUST00000025850 | NM_010235 | NM_010235 | Mm.6215 |
| Fra10ac1 | FRA10AC1 homolog (human) | Mus musculus FRA10AC1 homolog (human) (Fra10ac1), mRNA [NM_001081075] | chr19 | - | mm\|19qC3 | ENSMUST00000067167 | NM_001081075 | NM_001081075 | Mm.30607 |
| Gabpb1 | GA repeat binding protein, beta 1 | Mus musculus GA repeat binding protein, beta 1 (Gabpb1), transcript variant 2, mRNA [NM_010249] | chr2 | - | mm\|2qF1 | ENSMUST00000130263 | NM_010249 | NM_010249 | Mm.293266 |
| Gadd45a | growth arrest and DNA-damage-inducible 45 alpha | Mus musculus growth arrest and DNA-damage-inducible 45 alpha (Gadd45a), mRNA [NM_007836] | chr6 | - | mm\|6qC1 | ENSMUST00000043098 | NM_007836 | NM_007836 | Mm.72235 |
| Gadd45b | growth arrest and DNA-damage-inducible 45 beta | Mus musculus growth arrest and DNA-damage-inducible 45 beta (Gadd45b), mRNA [NM_008655] | chr10 | + | mm\|10qC1 | ENSMUST00000219449 | NM_008655 | NM_008655 | Mm.1360 |
| Galnt3 | UDP-N-acetyl-alpha-D-galactosamine:polypeptide N-acetylgalactosaminyltransferase 3 | Mus musculus UDP-N-acetyl-alpha-D-galactosamine:polypeptide N-acetylgalactosaminyltransferase 3 (Galnt3), mRNA [NM_015736] | chr2 | - | mm\|2qC1.3 | ENSMUST00000028378 | NM_015736 | NM_015736 | Mm.439760 |
| Gars | glycyl-tRNA synthetase | Mus musculus glycyl-tRNA synthetase (Gars), mRNA [NM_180678] | chr6 | + | mm\|6qB3 | ENSMUST00000003572 | NM_180678 | NM_180678 | Mm.250004 |
| Gatad1 | GATA zinc finger domain containing 1 | GATA zinc finger domain containing 1 [Source:MGI Symbol;Acc:MGI:1914460] [ENSMUST00000119783] | chr5 | - | mm\|5qA1 | ENSMUST00000119783 | BC019449 |  | Mm.178424 |
| Gdi2 | guanosine diphosphate (GDP) dissociation inhibitor 2 | Mus musculus guanosine diphosphate (GDP) dissociation inhibitor 2 (Gdi2), mRNA [NM_008112] | chr13 | + | mm\|13qA1 | ENSMUST00000059515 | NM_008112 | NM_008112 | Mm.153226 |
| Gfer | growth factor, erv1 (S. cerevisiae)-like (augmenter of liver regeneration) | Mus musculus growth factor, erv1 (S. cerevisiae)-like (augmenter of liver regeneration) (Gfer), mRNA [NM_023040] | chr17 | - | mm\|17qA3.3 | ENSMUST00000046839 | NM_023040 | NM_023040 | Mm.28124 |
| Gfm1 | G elongation factor, mitochondrial 1 | Mus musculus G elongation factor, mitochondrial 1 (Gfm1), mRNA [NM_138591] | chr3 | + | mm\|3qE1 | ENSMUST00000077271 | NM_138591 | NM_138591 | Mm.122466 |
| Gja1 | gap junction protein, alpha 1 | Mus musculus gap junction protein, alpha 1 (Gja1), mRNA [NM_010288] | chr10 | + | mm\|10qB4 | ENSMUST00000220069 | NM_010288 | NM_010288 | Mm.378921 |
| Glce | glucuronyl C5-epimerase | Mus musculus glucuronyl C5-epimerase (Glce), mRNA [NM_033320] | chr9 | - | mm\|9qB | ENSMUST00000185675 | NM_033320 | NM_033320 | Mm.24411 |
| Gltp | glycolipid transfer protein | Mus musculus glycolipid transfer protein (Gltp), mRNA [NM_019821] | chr5 | - | mm\|5qF | ENSMUST00000112214 | NM_019821 | NM_019821 | Mm.275766 |
| Gna12 | guanine nucleotide binding protein, alpha 12 | Mus musculus guanine nucleotide binding protein, alpha 12 (Gna12), mRNA [NM_010302] | chr5 | - | mm\|5qG2 | ENSMUST00000000153 | NM_010302 | NM_010302 | Mm.370185 |
| Gnb5 | guanine nucleotide binding protein (G protein), beta 5 | Mus musculus guanine nucleotide binding protein (G protein), beta 5 (Gnb5), transcript variant 1, mRNA [NM_010313] | chr9 | + | mm\|9qD | ENSMUST00000215346 | NM_010313 | NM_010313 | Mm.17604 |
| Gng12 | guanine nucleotide binding protein (G protein), gamma 12 | Mus musculus guanine nucleotide binding protein (G protein), gamma 12 (Gng12), transcript variant 2, mRNA [NM_025278] | chr6 | + | mm\|6qC1 | ENSMUST00000043148 | NM_025278 | NM_025278 | Mm.234342 |
| Gng5 | guanine nucleotide binding protein (G protein), gamma 5 | Mus musculus guanine nucleotide binding protein (G protein), gamma 5 (Gng5), mRNA [NM_010318] | chr3 | + | mm\|3qH2 | ENSMUST00000118280 | NM_010318 | NM_010318 | Mm.140804 |
| Gnpnat1 | glucosamine-phosphate N-acetyltransferase 1 | Mus musculus glucosamine-phosphate N-acetyltransferase 1 (Gnpnat1), mRNA [NM_019425] | chr14 | - | mm\|14qC1 | ENSMUST00000046191 | NM_019425 | NM_019425 | Mm.312945 |
| Got1 | glutamic-oxaloacetic transaminase 1, soluble | Mus musculus glutamic-oxaloacetic transaminase 1, soluble (Got1), mRNA [NM_010324] | chr19 | - | mm\|19qC3 | ENSMUST00000026196 | NM_010324 | NM_010324 | Mm.19039 |
| Got2 | glutamatic-oxaloacetic transaminase 2, mitochondrial | Mus musculus glutamatic-oxaloacetic transaminase 2, mitochondrial (Got2), mRNA [NM_010325] | chr8 | - | mm\|8qD1 | ENSMUST00000034097 | NM_010325 | NM_010325 | Mm.230169 |
| Gpam | glycerol-3-phosphate acyltransferase, mitochondrial | PREDICTED: Mus musculus glycerol-3-phosphate acyltransferase, mitochondrial (Gpam), transcript variant X3, mRNA [XM_011247147] | chr19 | - | mm\|19qD2 |  | XM_011247147 | XM_011247147 | Mm.210196 |
| Gpat3 | glycerol-3-phosphate acyltransferase 3 | Mus musculus glycerol-3-phosphate acyltransferase 3 (Gpat3), mRNA [NM_172715] | chr5 | + | mm\|5qE4 | ENSMUST00000112887 | NM_172715 | NM_172715 | Mm.271911 |
| Gpcpd1 | glycerophosphocholine phosphodiesterase 1 | Mus musculus glycerophosphocholine phosphodiesterase 1 (Gpcpd1), transcript variant 4, mRNA [NM_001042672] | chr2 | - | mm\|2qF2 | ENSMUST00000110135 | NM_001042672 | NM_001042672 | Mm.211211 |
| Gpr108 | G protein-coupled receptor 108 | Mus musculus G protein-coupled receptor 108 (Gpr108), transcript variant 1, mRNA [NM_030084] | chr17 | - | mm\|17qD | ENSMUST00000005975 | NM_030084 | NM_030084 | Mm.28468 |
| Gps1 | G protein pathway suppressor 1 | Mus musculus G protein pathway suppressor 1 (Gps1), transcript variant 1, mRNA [NM_145370] | chr11 | + | mm\|11qE2 | ENSMUST00000100134 | NM_145370 | NM_145370 | Mm.30195 |
| Gpt2 | glutamic pyruvate transaminase (alanine aminotransferase) 2 | Mus musculus glutamic pyruvate transaminase (alanine aminotransferase) 2 (Gpt2), mRNA [NM_173866] | chr8 | + | mm\|8qC3 | ENSMUST00000034136 | NM_173866 | NM_173866 | Mm.200423 |
| Grk2 | G protein-coupled receptor kinase 2 | Mus musculus adrenergic receptor kinase, beta 1 (Adrbk1), transcript variant 2, mRNA [NM_130863] | chr19 | - | mm\|19qA | ENSMUST00000171123 | NM_130863 | NM_130863 | Mm.254144 |
| Gspt1 | G1 to S phase transition 1 | Mus musculus G1 to S phase transition 1 (Gspt1), transcript variant 1, mRNA [NM_146066] | chr16 | - | mm\|16qA1 | ENSMUST00000080030 | NM_146066 | NM_146066 | Mm.325827 |
| Gsta5 |  |  |  |  |  |  |  |  |  |
| Gtf2f1 | general transcription factor IIF, polypeptide 1 | Mus musculus general transcription factor IIF, polypeptide 1 (Gtf2f1), mRNA [NM_133801] | chr17 | - | mm\|17qD | ENSMUST00000002733 | NM_133801 | NM_133801 | Mm.24632 |
| Gtf2f2 | general transcription factor IIF, polypeptide 2 | Mus musculus general transcription factor IIF, polypeptide 2 (Gtf2f2), mRNA [NM_026816] | chr14 | - | mm\|14qD3 | ENSMUST00000088922 | NM_026816 | NM_026816 | Mm.20415 |
| Gtf2h1 | general transcription factor II H, polypeptide 1 | Mus musculus general transcription factor II H, polypeptide 1 (Gtf2h1), transcript variant 2, mRNA [NM_008186] | chr7 | + | mm\|7qB4 | ENSMUST00000107644 | NM_008186 | NM_008186 | Mm.22700 |
| Gtf3a | general transcription factor III A | Mus musculus general transcription factor III A (Gtf3a), mRNA [NM_025652] | chr5 | + | mm\|5qG3 | ENSMUST00000146511 | NM_025652 | NM_025652 | Mm.196528 |
| Guf1 | GUF1 homolog, GTPase | Mus musculus GUF1 homolog, GTPase (Guf1), transcript variant 1, mRNA [NM_172711] | chr5 | + | mm\|5qC3.1 | ENSMUST00000125660 | NM_172711 | NM_172711 | Mm.21105 |
| Guk1 | guanylate kinase 1 | Mus musculus guanylate kinase 1 (Guk1), transcript variant 1, mRNA [NM_008193] | chr11 | - | mm\|11qB1.3 | ENSMUST00000151211 | NM_008193 | NM_008193 | Mm.3624 |
| Gzmm | granzyme M (lymphocyte met-ase 1) | Mus musculus granzyme M (lymphocyte met-ase 1) (Gzmm), transcript variant 1, mRNA [NM_008504] | chr10 | + | mm\|10qC1 | ENSMUST00000151213 | NM_008504 | NM_008504 | Mm.378960 |
| Hars | histidyl-tRNA synthetase | Mus musculus histidyl-tRNA synthetase (Hars), mRNA [NM_008214] | chr18 | - | mm\|18qB2 | ENSMUST00000001416 | NM_008214 | NM_008214 | Mm.10528 |
| Hccs | holocytochrome c synthetase | Mus musculus holocytochrome c synthetase (Hccs), transcript variant 1, mRNA [NM_001331049] | chrX | - | mm\|XqF5 | ENSMUST00000033717 | NM_001331049 | NM_001331049 | Mm.284033 |
| Hcfc2 | host cell factor C2 | Mus musculus host cell factor C2 (Hcfc2), mRNA [NM_001081218] | chr10 | + | mm\|10qC1 | ENSMUST00000020478 | NM_001081218 | NM_001081218 | Mm.127405 |
| Hdac8 | histone deacetylase 8 | Mus musculus histone deacetylase 8 (Hdac8), transcript variant 1, mRNA [NM_027382] | chrX | - | mm\|XqD | ENSMUST00000154872 | NM_027382 | NM_027382 | Mm.328128 |
| Heatr1 | HEAT repeat containing 1 | Mus musculus HEAT repeat containing 1 (Heatr1), mRNA [NM_144835] | chr13 | + | mm\|13qA1 | ENSMUST00000059270 | NM_144835 | NM_144835 | Mm.171186 |
| Hgs | HGF-regulated tyrosine kinase substrate | Mus musculus HGF-regulated tyrosine kinase substrate (Hgs), transcript variant 1, mRNA [NM_001159328] | chr11 | + | mm\|11qE2 | ENSMUST00000106203 | NM_001159328 | NM_001159328 | Mm.7919 |
| Hif1a | hypoxia inducible factor 1, alpha subunit | Mus musculus hypoxia inducible factor 1, alpha subunit (Hif1a), transcript variant 2, mRNA [NM_010431] | chr12 | + | mm\|12qC3 | ENSMUST00000021530 | NM_010431 | NM_010431 | Mm.3879 |
| Hint1 | histidine triad nucleotide binding protein 1 | Mus musculus histidine triad nucleotide binding protein 1 (Hint1), mRNA [NM_008248] | chr11 | + | mm\|11qB1.3 | ENSMUST00000020504 | NM_008248 | NM_008248 | Mm.425 |
| Hk2 | hexokinase 2 | Mus musculus hexokinase 2 (Hk2), mRNA [NM_013820] | chr6 | - | mm\|6qC3 | ENSMUST00000168725 | NM_013820 | NM_013820 | Mm.255848 |
| Hmga1 | high mobility group AT-hook 1 | Mus musculus high mobility group AT-hook 1 (Hmga1), transcript variant 14, mRNA [NM_001166546] | chr17 | + | mm\|17qA3.3 | ENSMUST00000118599 | NM_001166546 | NM_001166546 | Mm.4438 |
| Hmgn1 | high mobility group nucleosomal binding domain 1 | Mus musculus high mobility group nucleosomal binding domain 1 (Hmgn1), mRNA [NM_008251] | chr16 | - | mm\|16qC4 | ENSMUST00000145713 | NM_008251 | NM_008251 | Mm.2756 |
| Hnrnph3 | heterogeneous nuclear ribonucleoprotein H3 | heterogeneous nuclear ribonucleoprotein H3 [Source:MGI Symbol;Acc:MGI:1926462] [ENSMUST00000119814] | chr10 | - | mm\|10qB4 | ENSMUST00000119814 | BC049652 |  | Mm.274784 |
| Hsp90aa1 | heat shock protein 90, alpha (cytosolic), class A member 1 | 602797395F1 NCI_CGAP_Mam4 Mus musculus cDNA clone IMAGE:4918487 5', mRNA sequence [BG864327] | chr12 | - | mm\|12qF1 |  | BG864327 |  | Mm.315997 |
| Hsp90b1 | heat shock protein 90, beta (Grp94), member 1 | Mus musculus heat shock protein 90, beta (Grp94), member 1 (Hsp90b1), mRNA [NM_011631] | chr10 | - | mm\|10qC1 | ENSMUST00000020238 | NM_011631 | NM_011631 | Mm.87773 |
| Hspa4 | heat shock protein 4 | Mus musculus heat shock protein 4 (Hspa4), mRNA [NM_008300] | chr11 | - | mm\|11qB1.3 | ENSMUST00000020630 | NM_008300 | NM_008300 | Mm.239865 |
| Hspa5 | heat shock protein 5 | Mus musculus heat shock protein 5 (Hspa5), transcript variant 2, mRNA [NM_022310] | chr2 | + | mm\|2qB | ENSMUST00000100171 | NM_022310 | NM_022310 | Mm.330160 |
| Hspa9 | heat shock protein 9 | Mus musculus heat shock protein 9 (Hspa9), mRNA [NM_010481] | chr18 | - | mm\|18qB1 | ENSMUST00000025217 | NM_010481 | NM_010481 | Mm.209419 |
| Hspd1 | heat shock protein 1 (chaperonin) | Mus musculus heat shock protein 1 (chaperonin) (Hspd1), mRNA [NM_010477] | chr1 | - | mm\|1qC1.2 | ENSMUST00000027123 | NM_010477 | NM_010477 | Mm.1777 |
| Hspd1-ps3 |  |  |  |  |  |  |  |  |  |
| Hspe1 | heat shock protein 1 (chaperonin 10) | Mus musculus heat shock protein 1 (chaperonin 10) (Hspe1), mRNA [NM_008303] | chr1 | + | mm\|1qC1.2 | ENSMUST00000075242 | NM_008303 | NM_008303 | Mm.215667 |
| Htatip2 | HIV-1 Tat interactive protein 2 | Mus musculus HIV-1 Tat interactive protein 2 (Htatip2), transcript variant 1, mRNA [NM_016865] | chr7 | + | mm\|7qB5 | ENSMUST00000208048 | NM_016865 | NM_016865 | Mm.20801 |
| Huwe1 | HECT, UBA and WWE domain containing 1 | Mus musculus HECT, UBA and WWE domain containing 1 (Huwe1), mRNA [NM_021523] | chrX | + | mm\|XqF3 | ENSMUST00000026292 | NM_021523 | NM_021523 | Mm.27372 |
| Iars | isoleucine-tRNA synthetase | Mus musculus isoleucine-tRNA synthetase (Iars), mRNA [NM_172015] | chr13 | + | mm\|13qA5 | ENSMUST00000165316 | NM_172015 | NM_172015 | Mm.21118 |
| Ice1 | interactor of little elongation complex ELL subunit 1 | Mus musculus interactor of little elongation complex ELL subunit 1 (Ice1), mRNA [NM_144837] | chr13 | - | mm\|13qC1 | ENSMUST00000043493 | NM_144837 | NM_144837 | Mm.139738 |
| Icmt | isoprenylcysteine carboxyl methyltransferase | Mus musculus isoprenylcysteine carboxyl methyltransferase (Icmt), transcript variant 1, mRNA [NM_133788] | chr4 | + | mm\|4qE2 | ENSMUST00000125617 | NM_133788 | NM_133788 | Mm.277464 |
| Ifi47 | interferon gamma inducible protein 47 | Mus musculus interferon gamma inducible protein 47 (Ifi47), transcript variant 2, mRNA [NM_001271676] | chr11 | + | mm\|11qB1.2 | ENSMUST00000109202 | NM_001271676 | NM_001271676 | Mm.24769 |
| Ifih1 | interferon induced with helicase C domain 1 | Mus musculus interferon induced with helicase C domain 1 (Ifih1), transcript variant 1, mRNA [NM_027835] | chr2 | - | mm\|2qC1.3 | ENSMUST00000112459 | NM_027835 | NM_027835 | Mm.136224 |
| Ifrd1 | interferon-related developmental regulator 1 | Mus musculus interferon-related developmental regulator 1 (Ifrd1), mRNA [NM_013562] | chr12 | - | mm\|12qB1 | ENSMUST00000170119 | NM_013562 | NM_013562 | Mm.168 |
| Ift172 | intraflagellar transport 172 | Mus musculus intraflagellar transport 172 (Ift172), mRNA [NM_026298] | chr5 | - | mm\|5qB1 | ENSMUST00000202560 | NM_026298 | NM_026298 | Mm.293023 |
| Immp2l | IMP2 inner mitochondrial membrane peptidase-like (S. cerevisiae) | Mus musculus IMP2 inner mitochondrial membrane peptidase-like (S. cerevisiae) (Immp2l), mRNA [NM_053122] | chr12 | + | mm\|12qB1 | ENSMUST00000134965 | NM_053122 | NM_053122 | Mm.363813 |
| Inca1 | inhibitor of CDK, cyclin A1 interacting protein 1 | Mus musculus inhibitor of CDK, cyclin A1 interacting protein 1 (Inca1), transcript variant 5, mRNA [NM_001252485] | chr11 | - | mm\|11qB3 | ENSMUST00000108543 | NM_001252485 | NM_001252485 | Mm.101581 |
| Ints1 | integrator complex subunit 1 | Mus musculus integrator complex subunit 1 (Ints1), mRNA [NM_026748] | chr5 | - | mm\|5qG2 | ENSMUST00000200393 | NM_026748 | NM_026748 | Mm.292942 |
| Ipo5 | importin 5 | Mus musculus importin 5 (Ipo5), mRNA [NM_023579] | chr14 | + | mm\|14qE5 | ENSMUST00000032898 | NM_023579 | NM_023579 | Mm.221452 |
| Ipo7 | importin 7 | Mus musculus importin 7 (Ipo7), mRNA [NM_181517] | chr7 | + | mm\|7qF1 | ENSMUST00000207277 | NM_181517 | NM_181517 | Mm.222328 |
| Itga3 | integrin alpha 3 | Mus musculus integrin alpha 3 (Itga3), transcript variant 1, mRNA [NM_013565] | chr11 | - | mm\|11qD | ENSMUST00000001548 | NM_013565 | NM_013565 | Mm.57035 |
| Itgb1 | integrin beta 1 (fibronectin receptor beta) | Mus musculus integrin beta 1 (fibronectin receptor beta) (Itgb1), mRNA [NM_010578] | chr8 | + | mm\|8qE2 | ENSMUST00000124826 | NM_010578 | NM_010578 | Mm.263396 |
| Jkamp | JNK1/MAPK8-associated membrane protein | Mus musculus JNK1/MAPK8-associated membrane protein (Jkamp), transcript variant 1, mRNA [NM_024205] | chr12 | + | mm\|12qC3 | ENSMUST00000117449 | NM_024205 | NM_024205 | Mm.258935 |
| Jrkl | Jrk-like | Jrk-like [Source:MGI Symbol;Acc:MGI:1924782] [ENSMUST00000110582] | chr9_random | - |  | ENSMUST00000110582 |  |  |  |
| Junb | jun B proto-oncogene | Mus musculus jun B proto-oncogene (Junb), mRNA [NM_008416] | chr8 | - | mm\|8qC3 | ENSMUST00000064922 | NM_008416 | NM_008416 | Mm.1167 |
| Kansl2 | KAT8 regulatory NSL complex subunit 2 | PREDICTED: Mus musculus KAT8 regulatory NSL complex subunit 2 (Kansl2), transcript variant X1, mRNA [XM_006521360] | chr15 | - | mm\|15qF1 |  | XM_006521360 | XM_006521360 |  |
| Katnbl1 | katanin p80 subunit B like 1 | Mus musculus katanin p80 subunit B like 1 (Katnbl1), mRNA [NM_024254] | chr2 | + | mm\|2qE3 | ENSMUST00000028552 | NM_024254 | NM_024254 | Mm.425091 |
| Kdm7a | lysine (K)-specific demethylase 7A | Mus musculus lysine (K)-specific demethylase 7A (Kdm7a), mRNA [NM_001033430] | chr6 | - | mm\|6qB1 | ENSMUST00000002305 | NM_001033430 | NM_001033430 | Mm.293175 |
| Khdrbs1 | KH domain containing, RNA binding, signal transduction associated 1 | KH domain containing, RNA binding, signal transduction associated 1 [Source:MGI Symbol;Acc:MGI:893579] [ENSMUST00000129342] | chr4 | - | mm\|4qD2.2 | ENSMUST00000129342 | XR_001784112 | XR_001784112 |  |
| Kif2a | kinesin family member 2A | Mus musculus kinesin family member 2A (Kif2a), transcript variant 1, mRNA [NM_008442] | chr13 | - | mm\|13qD2.1 | ENSMUST00000159772 | NM_008442 | NM_008442 | Mm.355686 |
| Kif5b | kinesin family member 5B | Mus musculus kinesin family member 5B (Kif5b), mRNA [NM_008448] | chr18 | - | mm\|18qA1 | ENSMUST00000025083 | NM_008448 | NM_008448 | Mm.223744 |
| Kin | Kin17 DNA and RNA binding protein | Mus musculus Kin17 DNA and RNA binding protein (Kin), mRNA [NM_025280] | chr2 | + | mm\|2qA1 | ENSMUST00000042512 | NM_025280 | NM_025280 | Mm.35498 |
| Klhdc10 | kelch domain containing 10 | Mus musculus kelch domain containing 10 (Klhdc10), transcript variant 1, mRNA [NM_029742] | chr6 | + | mm\|6qA3.3 | ENSMUST00000068259 | NM_029742 | NM_029742 | Mm.277308 |
| Kpna1 | karyopherin (importin) alpha 1 | Mus musculus karyopherin (importin) alpha 1 (Kpna1), mRNA [NM_008465] | chr16 | + | mm\|16qB3 | ENSMUST00000004054 | NM_008465 | NM_008465 | Mm.6952 |
| Kpnb1 | karyopherin (importin) beta 1 | Mus musculus karyopherin (importin) beta 1 (Kpnb1), mRNA [NM_008379] | chr11 | - | mm\|11qD | ENSMUST00000001479 | NM_008379 | NM_008379 | Mm.251013 |
| Krtcap2 | keratinocyte associated protein 2 | Mus musculus keratinocyte associated protein 2 (Krtcap2), transcript variant 1, mRNA [NM_025327] | chr3 | + | mm\|3qF1 | ENSMUST00000168900 | NM_025327 | NM_025327 | Mm.177991 |
| Lactb | lactamase, beta | Mus musculus lactamase, beta (Lactb), mRNA [NM_030717] | chr9 | - | mm\|9qC | ENSMUST00000034929 | NM_030717 | NM_030717 | Mm.157882 |
| Lamtor2 | late endosomal/lysosomal adaptor, MAPK and MTOR activator 2 | Mus musculus late endosomal/lysosomal adaptor, MAPK and MTOR activator 2 (Lamtor2), mRNA [NM_031248] | chr3 | - | mm\|3qF1 | ENSMUST00000119002 | NM_031248 | NM_031248 | Mm.41680 |
| Larp4 | La ribonucleoprotein domain family, member 4 | Mus musculus La ribonucleoprotein domain family, member 4 (Larp4), transcript variant 2, mRNA [NM_001080948] | chr15 | + | mm\|15qF1 | ENSMUST00000057632 | NM_001080948 | NM_001080948 | Mm.28811 |
| Lars | leucyl-tRNA synthetase | Mus musculus leucyl-tRNA synthetase (Lars), mRNA [NM_134137] | chr18 | - | mm\|18qB3 | ENSMUST00000097590 | NM_134137 | NM_134137 | Mm.312170 |
| Leprotl1 | leptin receptor overlapping transcript-like 1 | Mus musculus leptin receptor overlapping transcript-like 1 (Leprotl1), mRNA [NM_026609] | chr8 | - | mm\|8qA4 | ENSMUST00000033910 | NM_026609 | NM_026609 | Mm.34212 |
| Lgals1 | lectin, galactose binding, soluble 1 | Mus musculus lectin, galactose binding, soluble 1 (Lgals1), mRNA [NM_008495] | chr15 | + | mm\|15qE1 | ENSMUST00000089377 | NM_008495 | NM_008495 | Mm.43831 |
| Lgals3 | lectin, galactose binding, soluble 3 | Mus musculus lectin, galactose binding, soluble 3 (Lgals3), transcript variant 1, mRNA [NM_001145953] | chr14 | + | mm\|14qC1 | ENSMUST00000150290 | NM_001145953 | NM_001145953 | Mm.248615 |
| Lgals8 | lectin, galactose binding, soluble 8 | Mus musculus lectin, galactose binding, soluble 8 (Lgals8), transcript variant 4, mRNA [NM_001291057] | chr13 | - | mm\|13qA1 | ENSMUST00000099820 | NM_001291057 | NM_001291057 | Mm.474138 |
| Lims1 | LIM and senescent cell antigen-like domains 1 | Mus musculus LIM and senescent cell antigen-like domains 1 (Lims1), transcript variant 1, mRNA [NM_026148] | chr10 | + | mm\|10qB4 | ENSMUST00000105468 | NM_026148 | NM_026148 | Mm.57734 |
| Llph | LLP homolog, long-term synaptic facilitation (Aplysia) | Mus musculus LLP homolog, long-term synaptic facilitation (Aplysia) (Llph), mRNA [NM_025431] | chr10 | + | mm\|10qD2 | ENSMUST00000130198 | NM_025431 | NM_025431 | Mm.291755 |
| Llph-ps2 |  |  |  |  |  |  |  |  |  |
| Lonp1 | lon peptidase 1, mitochondrial | Mus musculus lon peptidase 1, mitochondrial (Lonp1), mRNA [NM_028782] | chr17 | - | mm\|17qD | ENSMUST00000047226 | NM_028782 | NM_028782 | Mm.329136 |
| Lrrc41 | leucine rich repeat containing 41 | Mus musculus leucine rich repeat containing 41 (Lrrc41), mRNA [NM_153521] | chr4 | + | mm\|4qD1 | ENSMUST00000030471 | NM_153521 | NM_153521 | Mm.260786 |
| Lrrc59 | leucine rich repeat containing 59 | Mus musculus leucine rich repeat containing 59 (Lrrc59), mRNA [NM_133807] | chr11 | + | mm\|11qD | ENSMUST00000021239 | NM_133807 | NM_133807 | Mm.172720 |
| Ltv1 | LTV1 ribosome biogenesis factor | Mus musculus LTV1 ribosome biogenesis factor (Ltv1), mRNA [NM_181470] | chr10 | - | mm\|10qA2 | ENSMUST00000019950 | NM_181470 | NM_181470 | Mm.117581 |
| Lxn | latexin | Mus musculus latexin (Lxn), mRNA [NM_016753] | chr3 | - | mm\|3qE1 | ENSMUST00000160827 | NM_016753 | NM_016753 | Mm.2632 |
| Mafg | v-maf musculoaponeurotic fibrosarcoma oncogene family, protein G (avian) | Mus musculus v-maf musculoaponeurotic fibrosarcoma oncogene family, protein G (avian) (Mafg), mRNA [NM_010756] | chr11 | - | mm\|11qE2 | ENSMUST00000058162 | NM_010756 | NM_010756 | Mm.268010 |
| Man2a1 | mannosidase 2, alpha 1 | Mus musculus mannosidase 2, alpha 1 (Man2a1), mRNA [NM_008549] | chr17 | + | mm\|17qE1.1 | ENSMUST00000086723 | NM_008549 | NM_008549 | Mm.2433 |
| Manea | mannosidase, endo-alpha | Mus musculus mannosidase, endo-alpha (Manea), mRNA [NM_172865] | chr4 | - | mm\|4qA3 | ENSMUST00000041374 | NM_172865 | NM_172865 | Mm.245602 |
| Manf | mesencephalic astrocyte-derived neurotrophic factor | Mus musculus mesencephalic astrocyte-derived neurotrophic factor (Manf), mRNA [NM_029103] | chr9 | - | mm\|9qF1 | ENSMUST00000069036 | NM_029103 | NM_029103 | Mm.29778 |
| Map2k3os | mitogen-activated protein kinase kinase 3, opposite strand | Mus musculus mitogen-activated protein kinase kinase 3, opposite strand (Map2k3os), long non-coding RNA [NR_027800] | chr11 | - | mm\|11qB2 |  | NR_027800 | NR_027800 | Mm.46270 |
| Mapk6 | mitogen-activated protein kinase 6 | Mus musculus mitogen-activated protein kinase 6 (Mapk6), transcript variant 2, mRNA [NM_027418] | chr9 | - | mm\|9qD | ENSMUST00000049355 | NM_027418 | NM_027418 | Mm.480076 |
| Marchf2 |  |  |  |  |  |  |  |  |  |
| Mars1 |  |  |  |  |  |  |  |  |  |
| Mcm3ap | minichromosome maintenance complex component 3 associated protein | Mus musculus minichromosome maintenance complex component 3 associated protein (Mcm3ap), mRNA [NM_019434] | chr10 | + | mm\|10qC1 | ENSMUST00000170795 | NM_019434 | NM_019434 | Mm.30098 |
| Mdh2 | malate dehydrogenase 2, NAD (mitochondrial) | Mus musculus malate dehydrogenase 2, NAD (mitochondrial) (Mdh2), mRNA [NM_008617] | chr5 | + | mm\|5qG2 | ENSMUST00000019323 | NM_008617 | NM_008617 | Mm.297096 |
| Med31 | mediator complex subunit 31 | Mus musculus mediator complex subunit 31 (Med31), mRNA [NM_026068] | chr11 | - | mm\|11qB4 | ENSMUST00000021157 | NM_026068 | NM_026068 | Mm.159496 |
| Med7 | mediator complex subunit 7 | Mus musculus mediator complex subunit 7 (Med7), transcript variant 1, mRNA [NM_025426] | chr11 | + | mm\|11qB1.1 | ENSMUST00000020665 | NM_025426 | NM_025426 | Mm.24159 |
| Mesd | mesoderm development LRP chaperone | Mus musculus mesoderm development candidate 2 (Mesdc2), mRNA [NM_023403] | chr7 | + | mm\|7qD3 | ENSMUST00000094215 | NM_023403 | NM_023403 | Mm.117365 |
| Mettl9 | methyltransferase like 9 | Mus musculus methyltransferase like 9 (Mettl9), mRNA [NM_021554] | chr7 | + | mm\|7qF2 | ENSMUST00000033163 | NM_021554 | NM_021554 | Mm.29122 |
| Mfsd5 | major facilitator superfamily domain containing 5 | Mus musculus major facilitator superfamily domain containing 5 (Mfsd5), mRNA [NM_134100] | chr15 | + | mm\|15qF3 | ENSMUST00000051341 | NM_134100 | NM_134100 | Mm.287740 |
| Mib1 | mindbomb E3 ubiquitin protein ligase 1 | Mus musculus mindbomb E3 ubiquitin protein ligase 1 (Mib1), mRNA [NM_144860] | chr18 | + | mm\|18qA1 | ENSMUST00000165555 | NM_144860 | NM_144860 | Mm.21500 |
| Mier1 | MEIR1 treanscription regulator | Mus musculus MEIR1 treanscription regulator (Mier1), transcript variant 1, mRNA [NM_027696] | chr4 | + | mm\|4qC6 | ENSMUST00000106855 | NM_027696 | NM_027696 | Mm.288721 |
| Mlf2 | myeloid leukemia factor 2 | Mus musculus myeloid leukemia factor 2 (Mlf2), transcript variant 1, mRNA [NM_145385] | chr6 | + | mm\|6qF2 | ENSMUST00000032214 | NM_145385 | NM_145385 | Mm.490270 |
| Mlkl | mixed lineage kinase domain-like | Mus musculus mixed lineage kinase domain-like (Mlkl), transcript variant 1, mRNA [NM_001310613] | chr8 | - | mm\|8qE1 | ENSMUST00000120432 | NM_001310613 | NM_001310613 | Mm.207971 |
| Mllt11 | myeloid/lymphoid or mixed-lineage leukemia; translocated to, 11 | Mus musculus myeloid/lymphoid or mixed-lineage leukemia; translocated to, 11 (Mllt11), mRNA [NM_019914] | chr3 | - | mm\|3qF2.1 | ENSMUST00000065482 | NM_019914 | NM_019914 | Mm.331208 |
| Mnd1 | meiotic nuclear divisions 1 | Mus musculus meiotic nuclear divisions 1 (Mnd1), mRNA [NM_029797] | chr3 | - | mm\|3qF1 | ENSMUST00000047368 | NM_029797 | NM_029797 | Mm.274829 |
| Mogat2 | monoacylglycerol O-acyltransferase 2 | Mus musculus monoacylglycerol O-acyltransferase 2 (Mogat2), mRNA [NM_177448] | chr7 | - | mm\|7qE2 | ENSMUST00000064231 | NM_177448 | NM_177448 | Mm.208030 |
| Morf4l1-ps1 | mortality factor 4 like 1, pseudogene 1 | Mus musculus mortality factor 4 like 1, pseudogene 1 (Morf4l1-ps1), non-coding RNA [NR_038091] | chr16 | + | mm\|16qB1 |  | NR_038091 | NR_038091 | Mm.439775 |
| Mprip | myosin phosphatase Rho interacting protein | Mus musculus myosin phosphatase Rho interacting protein (Mprip), transcript variant 2, mRNA [NM_012027] | chr11 | + | mm\|11qB1.3 | ENSMUST00000066330 | NM_012027 | NM_012027 | Mm.2402 |
| Mrfap1 | Morf4 family associated protein 1 | Mus musculus Morf4 family associated protein 1 (Mrfap1), mRNA [NM_026242] | chr5 | - | mm\|5qB3 | ENSMUST00000068795 | NM_026242 | NM_026242 | Mm.490257 |
| Mrpl22 | mitochondrial ribosomal protein L22 | Mus musculus mitochondrial ribosomal protein L22 (Mrpl22), mRNA [NM_175001] | chr11 | + | mm\|11qB1.3 | ENSMUST00000020820 | NM_175001 | NM_175001 | Mm.259907 |
| Mrpl24 | mitochondrial ribosomal protein L24 | Mus musculus mitochondrial ribosomal protein L24 (Mrpl24), mRNA [NM_026591] | chr3 | + | mm\|3qF1 | ENSMUST00000119968 | NM_026591 | NM_026591 | Mm.393802 |
| Mrpl28 | mitochondrial ribosomal protein L28 | Mus musculus mitochondrial ribosomal protein L28 (Mrpl28), mRNA [NM_024227] | chr17 | + | mm\|17qA3.3 | ENSMUST00000123132 | NM_024227 | NM_024227 | Mm.273102 |
| Mrpl30 | mitochondrial ribosomal protein L30 | Mus musculus mitochondrial ribosomal protein L30 (Mrpl30), mRNA [NM_027098] | chr1 | + | mm\|1qB | ENSMUST00000027256 | NM_027098 | NM_027098 | Mm.26614 |
| Mrpl4 | mitochondrial ribosomal protein L4 | Mus musculus mitochondrial ribosomal protein L4 (Mrpl4), mRNA [NM_023167] | chr9 | + | mm\|9qA3 | ENSMUST00000213977 | NM_023167 | NM_023167 | Mm.427576 |
| Mrpl41 | mitochondrial ribosomal protein L41 | Mus musculus mitochondrial ribosomal protein L41 (Mrpl41), mRNA [NM_001031808] | chr2 | - | mm\|2qA3 |  | NM_001031808 | NM_001031808 | Mm.23010 |
| Mrpl43 | mitochondrial ribosomal protein L43 | Mus musculus mitochondrial ribosomal protein L43 (Mrpl43), mRNA [NM_053164] | chr19 | - | mm\|19qC3 | ENSMUST00000097715 | NM_053164 | NM_053164 | Mm.44174 |
| Mrpl57 | mitochondrial ribosomal protein L57 | Mus musculus mitochondrial ribosomal protein L57 (Mrpl57), mRNA [NM_026401] | chr14 | + | mm\|14qC3 | ENSMUST00000022538 | NM_026401 | NM_026401 | Mm.319530 |
| Mrps15 | mitochondrial ribosomal protein S15 | Mus musculus mitochondrial ribosomal protein S15 (Mrps15), mRNA [NM_025544] | chr4 | + | mm\|4qD2.2 | ENSMUST00000030675 | NM_025544 | NM_025544 | Mm.195628 |
| Mrps18b | mitochondrial ribosomal protein S18B | Mus musculus mitochondrial ribosomal protein S18B (Mrps18b), transcript variant 1, mRNA [NM_025878] | chr17 | - | mm\|17qB1 | ENSMUST00000172642 | NM_025878 | NM_025878 | Mm.29484 |
| Mrps18c | mitochondrial ribosomal protein S18C | Mus musculus mitochondrial ribosomal protein S18C (Mrps18c), mRNA [NM_026826] | chr5 | + | mm\|5qE4 | ENSMUST00000112898 | NM_026826 | NM_026826 | Mm.25585 |
| Mrps22 | mitochondrial ribosomal protein S22 | Mus musculus mitochondrial ribosomal protein S22 (Mrps22), mRNA [NM_025485] | chr9 | - | mm\|9qE3.3 | ENSMUST00000035034 | NM_025485 | NM_025485 | Mm.17949 |
| Mrps23 | mitochondrial ribosomal protein S23 | Mus musculus mitochondrial ribosomal protein S23 (Mrps23), transcript variant 3, mRNA [NM_001291271] | chr11 | + | mm\|11qC | ENSMUST00000107915 | NM_001291271 | NM_001291271 | Mm.168680 |
| Msh6 | mutS homolog 6 | Mus musculus mutS homolog 6 (Msh6), mRNA [NM_010830] | chr17 | + | mm\|17qE4 | ENSMUST00000005503 | NM_010830 | NM_010830 | Mm.18210 |
| Msrb1 | methionine sulfoxide reductase B1 | Mus musculus methionine sulfoxide reductase B1 (Msrb1), transcript variant 2, mRNA [NM_001346668] | chr17 | + | mm\|17qA3.3 | ENSMUST00000115262 | NM_001346668 | NM_001346668 | Mm.28212 |
| Mta1 | metastasis associated 1 | Mus musculus metastasis associated 1 (Mta1), transcript variant 2, mRNA [NM_001346696] | chr12 | + | mm\|12qF1 | ENSMUST00000130926 | NM_001346696 | NM_001346696 | Mm.212577 |
| Mtfr1l | mitochondrial fission regulator 1-like | Mus musculus mitochondrial fission regulator 1-like (Mtfr1l), transcript variant 1, mRNA [NM_001256112] | chr4 | - | mm\|4qD3 | ENSMUST00000146808 | NM_001256112 | NM_001256112 | Mm.30153 |
| Mtg1 | mitochondrial ribosome-associated GTPase 1 | Mus musculus mitochondrial ribosome-associated GTPase 1 (Mtg1), mRNA [NM_199301] | chr7 | + | mm\|7qF4 | ENSMUST00000036977 | NM_199301 | NM_199301 | Mm.296088 |
| Mthfd1 | methylenetetrahydrofolate dehydrogenase (NADP+ dependent), methenyltetrahydrofolate cyclohydrolase, formyltetrahydrofolate synthase | Mus musculus methylenetetrahydrofolate dehydrogenase (NADP+ dependent), methenyltetrahydrofolate cyclohydrolase, formyltetrahydrofolate synthase (Mthfd1), mRNA [NM_138745] | chr12 | + | mm\|12qC3 | ENSMUST00000220046 | NM_138745 | NM_138745 | Mm.29584 |
| Mthfd2 | methylenetetrahydrofolate dehydrogenase (NAD+ dependent), methenyltetrahydrofolate cyclohydrolase | Mus musculus methylenetetrahydrofolate dehydrogenase (NAD+ dependent), methenyltetrahydrofolate cyclohydrolase (Mthfd2), mRNA [NM_008638] | chr6 | - | mm\|6qC3 | ENSMUST00000203847 | NM_008638 | NM_008638 | Mm.443 |
| Mtln |  |  |  |  |  |  |  |  |  |
| Mtmr1 | myotubularin related protein 1 | Mus musculus myotubularin related protein 1 (Mtmr1), transcript variant 1, mRNA [NM_016985] | chrX | + | mm\|XqA7.2 | ENSMUST00000130179 | NM_016985 | NM_016985 | Mm.219672 |
| Mtmr2 | myotubularin related protein 2 | Mus musculus myotubularin related protein 2 (Mtmr2), transcript variant 1, mRNA [NM_023858] | chr9 | + | mm\|9qA1 | ENSMUST00000034396 | NM_023858 | NM_023858 | Mm.210405 |
| Mtmr6 | myotubularin related protein 6 | Mus musculus myotubularin related protein 6 (Mtmr6), mRNA [NM_144843] | chr14 | + | mm\|14qD1 | ENSMUST00000022563 | NM_144843 | NM_144843 | Mm.247007 |
| Mybbp1a | MYB binding protein (P160) 1a | Mus musculus MYB binding protein (P160) 1a (Mybbp1a), mRNA [NM_016776] | chr11 | + | mm\|11qB4 | ENSMUST00000152894 | NM_016776 | NM_016776 | Mm.147946 |
| Myl6 | myosin, light polypeptide 6, alkali, smooth muscle and non-muscle | Mus musculus myosin, light polypeptide 6, alkali, smooth muscle and non-muscle (Myl6), transcript variant 1, mRNA [NM_001317217] | chr10 | - | mm\|10qD3 | ENSMUST00000218813 | NM_001317217 | NM_001317217 | Mm.337074 |
| Nars | asparaginyl-tRNA synthetase | Mus musculus asparaginyl-tRNA synthetase (Nars), transcript variant 2, mRNA [NM_027350] | chr18 | - | mm\|18qE1 | ENSMUST00000025483 | NM_027350 | NM_027350 | Mm.29192 |
| Ncoa4-ps |  |  |  |  |  |  |  |  |  |
| Ndufa12 | NADH dehydrogenase (ubiquinone) 1 alpha subcomplex, 12 | Mus musculus NADH dehydrogenase (ubiquinone) 1 alpha subcomplex, 12 (Ndufa12), mRNA [NM_025551] | chr10 | + | mm\|10qC2 | ENSMUST00000179990 | NM_025551 | NM_025551 | Mm.27886 |
| Ndufa2 | NADH dehydrogenase (ubiquinone) 1 alpha subcomplex, 2 | Mus musculus NADH dehydrogenase (ubiquinone) 1 alpha subcomplex, 2 (Ndufa2), mRNA [NM_010885] | chr18 | - | mm\|18qB2 | ENSMUST00000014438 | NM_010885 | NM_010885 | Mm.29867 |
| Ndufa3 | NADH dehydrogenase (ubiquinone) 1 alpha subcomplex, 3 | NADH dehydrogenase (ubiquinone) 1 alpha subcomplex, 3 [Source:MGI Symbol;Acc:MGI:1913341] [ENSMUST00000150497] | chr7 | + | mm\|7qA1 | ENSMUST00000150497 | AK006243 |  | Mm.17851 |
| Ndufa5 | NADH dehydrogenase (ubiquinone) 1 alpha subcomplex, 5 | Mus musculus NADH dehydrogenase (ubiquinone) 1 alpha subcomplex, 5 (Ndufa5), transcript variant 1, mRNA [NM_026614] | chr6 | - | mm\|6qA3.1 | ENSMUST00000118558 | NM_026614 | NM_026614 | Mm.275780 |
| Ndufa8 | NADH dehydrogenase (ubiquinone) 1 alpha subcomplex, 8 | Mus musculus NADH dehydrogenase (ubiquinone) 1 alpha subcomplex, 8 (Ndufa8), mRNA [NM_026703] | chr2 | - | mm\|2qB | ENSMUST00000070112 | NM_026703 | NM_026703 | Mm.19834 |
| Ndufaf3 | NADH dehydrogenase (ubiquinone) 1 alpha subcomplex, assembly factor 3 | Mus musculus NADH dehydrogenase (ubiquinone) 1 alpha subcomplex, assembly factor 3 (Ndufaf3), mRNA [NM_023247] | chr9 | - | mm\|9qF2 | ENSMUST00000194666 | NM_023247 | NM_023247 | Mm.29471 |
| Ndufaf6 | NADH dehydrogenase (ubiquinone) complex I, assembly factor 6 | Mus musculus NADH dehydrogenase (ubiquinone) complex I, assembly factor 6 (Ndufaf6), mRNA [NM_001085493] | chr4 | - | mm\|4qA1 | ENSMUST00000058183 | NM_001085493 | NM_001085493 | Mm.275265 |
| Ndufb1 |  |  |  |  |  |  |  |  |  |
| Ndufb4 | NADH dehydrogenase (ubiquinone) 1 beta subcomplex 4 | Mus musculus NADH dehydrogenase (ubiquinone) 1 beta subcomplex 4 (Ndufb4), mRNA [NM_026610] | chr16 | - | mm\|16qB3 | ENSMUST00000023514 | NM_026610 | NM_026610 | Mm.379154 |
| Ndufs4 | NADH dehydrogenase (ubiquinone) Fe-S protein 4 | Mus musculus NADH dehydrogenase (ubiquinone) Fe-S protein 4 (Ndufs4), mRNA [NM_010887] | chr13 | - | mm\|13qD2.2 | ENSMUST00000022286 | NM_010887 | NM_010887 | Mm.253142 |
| Ndufs6 | NADH dehydrogenase (ubiquinone) Fe-S protein 6 | Mus musculus NADH dehydrogenase (ubiquinone) Fe-S protein 6 (Ndufs6), transcript variant 1, mRNA [NM_010888] | chr13 | - | mm\|13qC1 | ENSMUST00000022097 | NM_010888 | NM_010888 | Mm.29897 |
| Ndufs8 | NADH dehydrogenase (ubiquinone) Fe-S protein 8 | Mus musculus NADH dehydrogenase (ubiquinone) Fe-S protein 8 (Ndufs8), transcript variant 1, mRNA [NM_144870] | chr19 | - | mm\|19qA | ENSMUST00000075092 | NM_144870 | NM_144870 | Mm.44227 |
| Nek1 | NIMA (never in mitosis gene a)-related expressed kinase 1 | Mus musculus NIMA (never in mitosis gene a)-related expressed kinase 1 (Nek1), transcript variant 1, mRNA [NM_001293637] | chr8 | + | mm\|8qB3.1 | ENSMUST00000211256 | NM_001293637 | NM_001293637 | Mm.486881 |
| Nek3 | NIMA (never in mitosis gene a)-related expressed kinase 3 | Mus musculus NIMA (never in mitosis gene a)-related expressed kinase 3 (Nek3), transcript variant 1, mRNA [NM_001162947] | chr8 | - | mm\|8qA2 | ENSMUST00000110730 | NM_001162947 | NM_001162947 | Mm.41413 |
| Nenf | neuron derived neurotrophic factor | Mus musculus neuron derived neurotrophic factor (Nenf), mRNA [NM_025424] | chr1 | - | mm\|1qH6 | ENSMUST00000046770 | NM_025424 | NM_025424 | Mm.46444 |
| Nfkbil1 | nuclear factor of kappa light polypeptide gene enhancer in B cells inhibitor like 1 | Mus musculus nuclear factor of kappa light polypeptide gene enhancer in B cells inhibitor like 1 (Nfkbil1), mRNA [NM_010909] | chr17 | - | mm\|17qB1 | ENSMUST00000048994 | NM_010909 | NM_010909 | Mm.300795 |
| Nfu1 | NFU1 iron-sulfur cluster scaffold | Mus musculus NFU1 iron-sulfur cluster scaffold (Nfu1), transcript variant 1, mRNA [NM_001170591] | chr6 | + | mm\|6qD1 | ENSMUST00000120240 | NM_001170591 | NM_001170591 | Mm.23809 |
| Ngdn | neuroguidin, EIF4E binding protein | Mus musculus neuroguidin, EIF4E binding protein (Ngdn), mRNA [NM_026890] | chr14 | + | mm\|14qC3 | ENSMUST00000022815 | NM_026890 | NM_026890 | Mm.21214 |
| Nmt1 | N-myristoyltransferase 1 | Mus musculus N-myristoyltransferase 1 (Nmt1), mRNA [NM_008707] | chr11 | + | mm\|11qE1 | ENSMUST00000021314 | NM_008707 | NM_008707 | Mm.10265 |
| Nol11 | nucleolar protein 11 | Mus musculus nucleolar protein 11 (Nol11), transcript variant 1, mRNA [NM_133702] | chr11 | - | mm\|11qE1 | ENSMUST00000106757 | NM_133702 | NM_133702 | Mm.294617 |
| Nomo1 | nodal modulator 1 | Mus musculus nodal modulator 1 (Nomo1), mRNA [NM_153057] | chr7 | + | mm\|7qB4 | ENSMUST00000033121 | NM_153057 | NM_153057 | Mm.274811 |
| Nop14 | NOP14 nucleolar protein | Mus musculus NOP14 nucleolar protein (Nop14), mRNA [NM_029278] | chr5 | - | mm\|5qB2 | ENSMUST00000152393 | NM_029278 | NM_029278 | Mm.40292 |
| Nphp1 | nephronophthisis 1 (juvenile) homolog (human) | Mus musculus nephronophthisis 1 (juvenile) homolog (human) (Nphp1), transcript variant 3, mRNA [NM_001291013] | chr2 | - | mm\|2qF1 | ENSMUST00000110357 | NM_001291013 | NM_001291013 | Mm.210766 |
| Nploc4 | NPL4 homolog, ubiquitin recognition factor | Mus musculus NPL4 homolog, ubiquitin recognition factor (Nploc4), transcript variant A, mRNA [NM_001195023] | chr11 | - | mm\|11qE2 | ENSMUST00000044271 | NM_001195023 | NM_001195023 | Mm.309520 |
| Npm1 | nucleophosmin 1 | Mus musculus nucleophosmin 1 (Npm1), transcript variant 3, mRNA [NM_001252261] | chr11 | - | mm\|11qA4 | ENSMUST00000146759 | NM_001252261 | NM_001252261 | Mm.485384 |
| Npm3 | nucleoplasmin 3 | Mus musculus nucleoplasmin 3 (Npm3), mRNA [NM_008723] | chr19 | - | mm\|19qC3 | ENSMUST00000070215 | NM_008723 | NM_008723 | Mm.1406 |
| Nqo2 | N-ribosyldihydronicotinamide quinone reductase 2 | Mus musculus N-ribosyldihydronicotinamide quinone reductase 2 (Nqo2), transcript variant 1, mRNA [NM_020282] | chr13 | + | mm\|13qA3.3 | ENSMUST00000021843 | NM_020282 | NM_020282 | Mm.264036 |
| Nr2c1 | nuclear receptor subfamily 2, group C, member 1 | Mus musculus nuclear receptor subfamily 2, group C, member 1 (Nr2c1), mRNA [NM_011629] | chr10 | + | mm\|10qC2 | ENSMUST00000105290 | NM_011629 | NM_011629 | Mm.107483 |
| Nrp2 | neuropilin 2 | Mus musculus neuropilin 2 (Nrp2), transcript variant 5, mRNA [NM_001077406] | chr1 | + | mm\|1qC2 | ENSMUST00000027112 | NM_001077406 | NM_001077406 | Mm.266341 |
| Nsmce1 | NSE1 homolog, SMC5-SMC6 complex component | Mus musculus NSE1 homolog, SMC5-SMC6 complex component (Nsmce1), mRNA [NM_026330] | chr7 | - | mm\|7qF3 | ENSMUST00000033006 | NM_026330 | NM_026330 | Mm.4467 |
| Nsun2 | NOL1/NOP2/Sun domain family member 2 | Mus musculus NOL1/NOP2/Sun domain family member 2 (Nsun2), mRNA [NM_145354] | chr13 | + | mm\|13qC1 | ENSMUST00000109699 | NM_145354 | NM_145354 | Mm.260009 |
| Nt5dc2 | 5'-nucleotidase domain containing 2 | Mus musculus 5'-nucleotidase domain containing 2 (Nt5dc2), mRNA [NM_027289] | chr14 | + | mm\|14qB | ENSMUST00000090212 | NM_027289 | NM_027289 | Mm.261681 |
| Ntan1 | N-terminal Asn amidase | Mus musculus N-terminal Asn amidase (Ntan1), transcript variant 1, mRNA [NM_010946] | chr16 | + | mm\|16qA1 | ENSMUST00000139161 | NM_010946 | NM_010946 | Mm.380410 |
| Nthl1 | nth (endonuclease III)-like 1 (E.coli) | Mus musculus nth (endonuclease III)-like 1 (E.coli) (Nthl1), mRNA [NM_008743] | chr17 | + | mm\|17qA3.3 | ENSMUST00000047611 | NM_008743 | NM_008743 | Mm.148315 |
| Nucb2 | nucleobindin 2 | Mus musculus nucleobindin 2 (Nucb2), mRNA [NM_001130479] | chr7 | + | mm\|7qF1 | ENSMUST00000032895 | NM_001130479 | NM_001130479 | Mm.9901 |
| Nudcd2 | NudC domain containing 2 | Mus musculus NudC domain containing 2 (Nudcd2), transcript variant 1, mRNA [NM_026023] | chr11 | + | mm\|11qA5 | ENSMUST00000141839 | NM_026023 | NM_026023 | Mm.276504 |
| Nudt8 | nudix (nucleoside diphosphate linked moiety X)-type motif 8 | Mus musculus nudix (nucleoside diphosphate linked moiety X)-type motif 8 (Nudt8), mRNA [NM_025529] | chr19 | + | mm\|19qA | ENSMUST00000122924 | NM_025529 | NM_025529 | Mm.259222 |
| Nup188 | nucleoporin 188 | Mus musculus nucleoporin 188 (Nup188), mRNA [NM_198304] | chr2 | + | mm\|2qB | ENSMUST00000064447 | NM_198304 | NM_198304 | Mm.330119 |
| Nupr1 | nuclear protein transcription regulator 1 | Mus musculus nuclear protein transcription regulator 1 (Nupr1), mRNA [NM_019738] | chr7 | - | mm\|7qF3 | ENSMUST00000187609 | NM_019738 | NM_019738 | Mm.18742 |
| Odc1 | ornithine decarboxylase, structural 1 | Mus musculus ornithine decarboxylase, structural 1 (Odc1), mRNA [NM_013614] | chr12 | + | mm\|12qA1.1 | ENSMUST00000171737 | NM_013614 | NM_013614 | Mm.34102 |
| Ofd1 | OFD1, centriole and centriolar satellite protein | Mus musculus oral-facial-digital syndrome 1 gene homolog (human) (Ofd1), mRNA [NM_177429] | chrX | - | mm\|XqF5 | ENSMUST00000049501 | NM_177429 | NM_177429 | Mm.247480 |
| Opa1 | OPA1, mitochondrial dynamin like GTPase | Mus musculus OPA1, mitochondrial dynamin like GTPase (Opa1), transcript variant 1, mRNA [NM_001199177] | chr16 | + | mm\|16qB2 | ENSMUST00000038867 | NM_001199177 | NM_001199177 | Mm.274285 |
| Orc3 | origin recognition complex, subunit 3 | Mus musculus origin recognition complex, subunit 3 (Orc3), transcript variant 1, mRNA [NM_015824] | chr4 | - | mm\|4qA5 |  | NM_015824 | NM_015824 | Mm.399624 |
| Otub1 | OTU domain, ubiquitin aldehyde binding 1 | Mus musculus OTU domain, ubiquitin aldehyde binding 1 (Otub1), mRNA [NM_134150] | chr19 | - | mm\|19qA | ENSMUST00000025679 | NM_134150 | NM_134150 | Mm.203921 |
| Oxct1 | 3-oxoacid CoA transferase 1 | Mus musculus 3-oxoacid CoA transferase 1 (Oxct1), mRNA [NM_024188] | chr15 | + | mm\|15qA1 | ENSMUST00000110690 | NM_024188 | NM_024188 | Mm.13445 |
| Oxld1 | oxidoreductase like domain containing 1 | Mus musculus oxidoreductase like domain containing 1 (Oxld1), mRNA [NM_025560] | chr11 | - | mm\|11qE2 | ENSMUST00000044007 | NM_025560 | NM_025560 | Mm.45063 |
| Oxnad1 | oxidoreductase NAD-binding domain containing 1 | Mus musculus oxidoreductase NAD-binding domain containing 1 (Oxnad1), mRNA [NM_145460] | chr14 | + | mm\|14qB | ENSMUST00000022462 | NM_145460 | NM_145460 | Mm.202257 |
| Pa2g4 | proliferation-associated 2G4 | Mus musculus proliferation-associated 2G4 (Pa2g4), mRNA [NM_011119] | chr10 | - | mm\|10qD3 | ENSMUST00000131728 | NM_011119 | NM_011119 | Mm.4742 |
| Pacc1 |  |  |  |  |  |  |  |  |  |
| Pam16 | presequence translocase-asssociated motor 16 homolog (S. cerevisiae) | Mus musculus presequence translocase-asssociated motor 16 homolog (S. cerevisiae) (Pam16), mRNA [NM_025571] | chr16 | - | mm\|16qA1 | ENSMUST00000014445 | NM_025571 | NM_025571 | Mm.354760 |
| Paqr3 | progestin and adipoQ receptor family member III | Mus musculus progestin and adipoQ receptor family member III (Paqr3), mRNA [NM_198422] | chr5 | - | mm\|5qE3 | ENSMUST00000196078 | NM_198422 | NM_198422 | Mm.332505 |
| Park7 | Parkinson disease (autosomal recessive, early onset) 7 | Mus musculus Parkinson disease (autosomal recessive, early onset) 7 (Park7), mRNA [NM_020569] | chr4 | - | mm\|4qE2 | ENSMUST00000105674 | NM_020569 | NM_020569 | Mm.277349 |
| Parl | presenilin associated, rhomboid-like | Mus musculus presenilin associated, rhomboid-like (Parl), mRNA [NM_001005767] | chr16 | - | mm\|16qA3 | ENSMUST00000120117 | NM_001005767 | NM_001005767 | Mm.371737 |
| Parn | poly(A)-specific ribonuclease (deadenylation nuclease) | Mus musculus poly(A)-specific ribonuclease (deadenylation nuclease) (Parn), mRNA [NM_028761] | chr16 | - | mm\|16qA1 | ENSMUST00000058884 | NM_028761 | NM_028761 | Mm.182350 |
| Pcbd2 | pterin 4 alpha carbinolamine dehydratase/dimerization cofactor of hepatocyte nuclear factor 1 alpha (TCF1) 2 | Mus musculus pterin 4 alpha carbinolamine dehydratase/dimerization cofactor of hepatocyte nuclear factor 1 alpha (TCF1) 2 (Pcbd2), mRNA [NM_028281] | chr13 | + | mm\|13qB1 | ENSMUST00000021958 | NM_028281 | NM_028281 | Mm.28145 |
| Pcdhgc3 |  |  |  |  |  |  |  |  |  |
| Pdcd11 | programmed cell death 11 | Mus musculus programmed cell death 11 (Pdcd11), mRNA [NM_011053] | chr19 | + | mm\|19qC3 | ENSMUST00000072141 | NM_011053 | NM_011053 | Mm.41166 |
| Pdia3 | protein disulfide isomerase associated 3 | Mus musculus protein disulfide isomerase associated 3 (Pdia3), mRNA [NM_007952] | chr2 | + | mm\|2qE5 | ENSMUST00000028683 | NM_007952 | NM_007952 | Mm.263177 |
| Pdia5 | protein disulfide isomerase associated 5 | Mus musculus protein disulfide isomerase associated 5 (Pdia5), mRNA [NM_028295] | chr16 | - | mm\|16qB3 | ENSMUST00000023550 | NM_028295 | NM_028295 | Mm.71015 |
| Pdrg1 | p53 and DNA damage regulated 1 | Mus musculus p53 and DNA damage regulated 1 (Pdrg1), mRNA [NM_178939] | chr2 | - | mm\|2qH1 | ENSMUST00000150545 | NM_178939 | NM_178939 | Mm.12746 |
| Pdxdc1 | pyridoxal-dependent decarboxylase domain containing 1 | Mus musculus pyridoxal-dependent decarboxylase domain containing 1 (Pdxdc1), transcript variant 1, mRNA [NM_053181] | chr16 | - | mm\|16qA1 | ENSMUST00000154150 | NM_053181 | NM_053181 | Mm.171484 |
| Pex11b | peroxisomal biogenesis factor 11 beta | Mus musculus peroxisomal biogenesis factor 11 beta (Pex11b), transcript variant 1, mRNA [NM_011069] | chr3 | + | mm\|3qF2.1 | ENSMUST00000165842 | NM_011069 | NM_011069 | Mm.20901 |
| Pfn1 | profilin 1 | profilin 1 [Source:MGI Symbol;Acc:MGI:97549] [ENSMUST00000108549] | chr11 | - | mm\|11qB3 | ENSMUST00000108549 | AK034932 |  |  |
| Pgam5 | phosphoglycerate mutase family member 5 | Mus musculus phosphoglycerate mutase family member 5 (Pgam5), transcript variant 1, mRNA [NM_001163538] | chr5 | - | mm\|5qF | ENSMUST00000112505 | NM_001163538 | NM_001163538 | Mm.61682 |
| Pgls | 6-phosphogluconolactonase | Mus musculus 6-phosphogluconolactonase (Pgls), transcript variant 2, mRNA [NM_001294269] | chr8 | + | mm\|8qB3.3 | ENSMUST00000138742 | NM_001294269 | NM_001294269 | Mm.282284 |
| Phf10 | PHD finger protein 10 | Mus musculus PHD finger protein 10 (Phf10), mRNA [NM_024250] | chr17 | - | mm\|17qA2 | ENSMUST00000024657 | NM_024250 | NM_024250 | Mm.440085 |
| Phgdh | 3-phosphoglycerate dehydrogenase | Mus musculus 3-phosphoglycerate dehydrogenase (Phgdh), mRNA [NM_016966] | chr3 | - | mm\|3qF2.2 | ENSMUST00000209921 | NM_016966 | NM_016966 | Mm.16898 |
| Picalm | phosphatidylinositol binding clathrin assembly protein | Mus musculus phosphatidylinositol binding clathrin assembly protein (Picalm), transcript variant 1, mRNA [NM_146194] | chr7 | + | mm\|7qE1 | ENSMUST00000208730 | NM_146194 | NM_146194 | Mm.235175 |
| Pigf | phosphatidylinositol glycan anchor biosynthesis, class F | Mus musculus phosphatidylinositol glycan anchor biosynthesis, class F (Pigf), mRNA [NM_008838] | chr17 | - | mm\|17qE4 | ENSMUST00000024957 | NM_008838 | NM_008838 | Mm.219685 |
| Pigt | phosphatidylinositol glycan anchor biosynthesis, class T | phosphatidylinositol glycan anchor biosynthesis, class T [Source:MGI Symbol;Acc:MGI:1926178] [ENSMUST00000117066] | chr2 | + | mm\|2qH3 | ENSMUST00000117066 | AK090020 |  | Mm.28228 |
| Pigyl | phosphatidylinositol glycan anchor biosynthesis, class Y-like | Mus musculus phosphatidylinositol glycan anchor biosynthesis, class Y-like (Pigyl), mRNA [NM_001082532] | chr9 | + | mm\|9qA3 | ENSMUST00000123680 | NM_001082532 | NM_001082532 | Mm.28924 |
| Pik3cb | phosphatidylinositol-4,5-bisphosphate 3-kinase catalytic subunit beta | Mus musculus phosphatidylinositol 3-kinase, catalytic, beta polypeptide (Pik3cb), mRNA [NM_029094] | chr9 | - | mm\|9qE3.3 | ENSMUST00000136965 | NM_029094 | NM_029094 | Mm.213128 |
| Pim2 | proviral integration site 2 | Mus musculus proviral integration site 2 (Pim2), mRNA [NM_138606] | chrX | + | mm\|XqA1.1 | ENSMUST00000033495 | NM_138606 | NM_138606 | Mm.347478 |
| Pitpna | phosphatidylinositol transfer protein, alpha | Mus musculus phosphatidylinositol transfer protein, alpha (Pitpna), mRNA [NM_008850] | chr11 | + | mm\|11qB5 | ENSMUST00000179445 | NM_008850 | NM_008850 | Mm.3128 |
| Plec | plectin | Mus musculus plectin (Plec), transcript variant 13, mRNA [NM_001163540] | chr15 | - | mm\|15qD3 | ENSMUST00000073418 | NM_001163540 | NM_001163540 | Mm.234912 |
| Plek2 | pleckstrin 2 | Mus musculus pleckstrin 2 (Plek2), mRNA [NM_013738] | chr12 | - | mm\|12qC3 | ENSMUST00000021544 | NM_013738 | NM_013738 | Mm.103380 |
| Plpp1 | phospholipid phosphatase 1 | Mus musculus phospholipid phosphatase 1 (Plpp1), transcript variant 1, mRNA [NM_008247] | chr13 | + | mm\|13qD2.2 | ENSMUST00000070951 | NM_008247 | NM_008247 | Mm.317186 |
| Pmpca | peptidase (mitochondrial processing) alpha | peptidase (mitochondrial processing) alpha [Source:MGI Symbol;Acc:MGI:1918568] [ENSMUST00000114093] | chr2 | + | mm\|2qA3 | ENSMUST00000114093 | AK032081 |  |  |
| Pno1 | partner of NOB1 homolog | Mus musculus partner of NOB1 homolog (Pno1), mRNA [NM_025443] | chr11 | - | mm\|11qA2 | ENSMUST00000020317 | NM_025443 | NM_025443 | Mm.27831 |
| Pnpt1 | polyribonucleotide nucleotidyltransferase 1 | Mus musculus polyribonucleotide nucleotidyltransferase 1 (Pnpt1), mRNA [NM_027869] | chr11 | + | mm\|11qA3.3 | ENSMUST00000154924 | NM_027869 | NM_027869 | Mm.211131 |
| Poglut2 |  |  |  |  |  |  |  |  |  |
| Polb | polymerase (DNA directed), beta | Mus musculus polymerase (DNA directed), beta (Polb), mRNA [NM_011130] | chr8 | - | mm\|8qA2 | ENSMUST00000033938 | NM_011130 | NM_011130 | Mm.123211 |
| Polr2i | polymerase (RNA) II (DNA directed) polypeptide I | polymerase (RNA) II (DNA directed) polypeptide I [Source:MGI Symbol;Acc:MGI:1917170] [ENSMUST00000108193] | chr7 | + | mm\|7qB1 | ENSMUST00000108193 |  |  |  |
| Polr2j | polymerase (RNA) II (DNA directed) polypeptide J | Mus musculus polymerase (RNA) II (DNA directed) polypeptide J (Polr2j), mRNA [NM_011293] | chr5 | + | mm\|5qG2 | ENSMUST00000111129 | NM_011293 | NM_011293 | Mm.4896 |
| Pop4 | processing of precursor 4, ribonuclease P/MRP family, (S. cerevisiae) | Mus musculus processing of precursor 4, ribonuclease P/MRP family, (S. cerevisiae) (Pop4), mRNA [NM_025390] | chr7 | - | mm\|7qB2 | ENSMUST00000032585 | NM_025390 | NM_025390 | Mm.22284 |
| Ppib | peptidylprolyl isomerase B | Mus musculus peptidylprolyl isomerase B (Ppib), mRNA [NM_011149] | chr9 | + | mm\|9qC | ENSMUST00000213785 | NM_011149 | NM_011149 | Mm.335249 |
| Ppid | peptidylprolyl isomerase D (cyclophilin D) | Mus musculus peptidylprolyl isomerase D (cyclophilin D) (Ppid), mRNA [NM_026352] | chr3 | + | mm\|3qE3 | ENSMUST00000199720 | NM_026352 | NM_026352 | Mm.295252 |
| Ppp1cb | protein phosphatase 1, catalytic subunit, beta isoform | protein phosphatase 1, catalytic subunit, beta isoform [Source:MGI Symbol;Acc:MGI:104871] [ENSMUST00000015100] | chr5 | + | mm\|5qB1 | ENSMUST00000015100 |  |  |  |
| Ppp1r12a | protein phosphatase 1, regulatory (inhibitor) subunit 12A | Mus musculus protein phosphatase 1, regulatory (inhibitor) subunit 12A (Ppp1r12a), mRNA [NM_027892] | chr10 | + | mm\|10qD1 | ENSMUST00000070663 | NM_027892 | NM_027892 | Mm.422959 |
| Ppp1r14b | protein phosphatase 1, regulatory (inhibitor) subunit 14B |  | chr19 | + | mm\|19qA |  | BC082545 |  | Mm.140 |
| Ppp1r15a | protein phosphatase 1, regulatory (inhibitor) subunit 15A | Mus musculus protein phosphatase 1, regulatory (inhibitor) subunit 15A (Ppp1r15a), mRNA [NM_008654] | chr7 | - | mm\|7qB4 | ENSMUST00000167273 | NM_008654 | NM_008654 | Mm.4048 |
| Ppp2r5d | protein phosphatase 2, regulatory subunit B', delta | Mus musculus protein phosphatase 2, regulatory subunit B', delta (Ppp2r5d), mRNA [NM_009358] | chr17 | - | mm\|17qC | ENSMUST00000002839 | NM_009358 | NM_009358 | Mm.295009 |
| Prelid3b | PRELI domain containing 3B | Mus musculus PRELI domain containing 3B (Prelid3b), mRNA [NM_025531] | chr2 | - | mm\|2qH4 | ENSMUST00000016401 | NM_025531 | NM_025531 | Mm.182294 |
| Prepl | prolyl endopeptidase-like | Mus musculus prolyl endopeptidase-like (Prepl), transcript variant 1, mRNA [NM_001163622] | chr17 | - | mm\|17qE4 | ENSMUST00000171795 | NM_001163622 | NM_001163622 | Mm.461481 |
| Prkcg | protein kinase C, gamma | Mus musculus protein kinase C, gamma (Prkcg), transcript variant 1, mRNA [NM_011102] | chr7 | + | mm\|7qA1 | ENSMUST00000181455 | NM_011102 | NM_011102 | Mm.7980 |
| Prkci | protein kinase C, iota | Mus musculus protein kinase C, iota (Prkci), mRNA [NM_008857] | chr3 | + | mm\|3qA3 | ENSMUST00000108249 | NM_008857 | NM_008857 | Mm.291554 |
| Prmt7 | protein arginine N-methyltransferase 7 | Mus musculus protein arginine N-methyltransferase 7 (Prmt7), mRNA [NM_145404] | chr8 | + | mm\|8qD3 | ENSMUST00000071592 | NM_145404 | NM_145404 | Mm.251804 |
| Prpf40b | pre-mRNA processing factor 40B | Mus musculus pre-mRNA processing factor 40B (Prpf40b), transcript variant 2, mRNA [NM_018786] | chr15 | + | mm\|15qF1 | ENSMUST00000124275 | NM_018786 | NM_018786 | Mm.358668 |
| Prpf8 | pre-mRNA processing factor 8 | Mus musculus pre-mRNA processing factor 8 (Prpf8), mRNA [NM_138659] | chr11 | + | mm\|11qB5 | ENSMUST00000018449 | NM_138659 | NM_138659 | Mm.3757 |
| Prps1 | phosphoribosyl pyrophosphate synthetase 1 | Mus musculus phosphoribosyl pyrophosphate synthetase 1 (Prps1), mRNA [NM_021463] | chrX | + | mm\|XqF1 | ENSMUST00000033809 | NM_021463 | NM_021463 | Mm.287178 |
| Psat1 | phosphoserine aminotransferase 1 | Mus musculus phosphoserine aminotransferase 1 (Psat1), transcript variant 2, mRNA [NM_001205339] | chr19 | - | mm\|19qA | ENSMUST00000025542 | NM_001205339 | NM_001205339 | Mm.289936 |
| Psma1 | proteasome (prosome, macropain) subunit, alpha type 1 | Mus musculus proteasome (prosome, macropain) subunit, alpha type 1 (Psma1), mRNA [NM_011965] | chr7 | - | mm\|7qF1 | ENSMUST00000033008 | NM_011965 | NM_011965 | Mm.121265 |
| Psma2 | proteasome (prosome, macropain) subunit, alpha type 2 | Mus musculus proteasome (prosome, macropain) subunit, alpha type 2 (Psma2), mRNA [NM_008944] | chr13 | + | mm\|13qA1 | ENSMUST00000170836 | NM_008944 | NM_008944 | Mm.252255 |
| Psma3 | proteasome (prosome, macropain) subunit, alpha type 3 | Mus musculus proteasome (prosome, macropain) subunit, alpha type 3 (Psma3), transcript variant 2, mRNA [NM_001310595] | chr12 | + | mm\|12qC3 | ENSMUST00000162851 | NM_001310595 | NM_001310595 | Mm.296338 |
| Psma4 | proteasome (prosome, macropain) subunit, alpha type 4 | Mus musculus proteasome (prosome, macropain) subunit, alpha type 4 (Psma4), mRNA [NM_011966] | chr9 | + | mm\|9qB | ENSMUST00000153606 | NM_011966 | NM_011966 | Mm.30270 |
| Psmb7 | proteasome (prosome, macropain) subunit, beta type 7 | Mus musculus proteasome (prosome, macropain) subunit, beta type 7 (Psmb7), mRNA [NM_011187] | chr2 | - | mm\|2qB | ENSMUST00000028083 | NM_011187 | NM_011187 | Mm.389251 |
| Psmc2 | proteasome (prosome, macropain) 26S subunit, ATPase 2 | Mus musculus proteasome (prosome, macropain) 26S subunit, ATPase 2 (Psmc2), mRNA [NM_011188] | chr5 | + | mm\|5qA3 | ENSMUST00000030769 | NM_011188 | NM_011188 | Mm.2462 |
| Psmc6 | proteasome (prosome, macropain) 26S subunit, ATPase, 6 | Mus musculus proteasome (prosome, macropain) 26S subunit, ATPase, 6 (Psmc6), mRNA [NM_025959] | chr14 | + | mm\|14qC1 | ENSMUST00000022380 | NM_025959 | NM_025959 | Mm.18472 |
| Psmd10 | proteasome (prosome, macropain) 26S subunit, non-ATPase, 10 | Mus musculus proteasome (prosome, macropain) 26S subunit, non-ATPase, 10 (Psmd10), transcript variant 1, mRNA [NM_016883] | chrX | - | mm\|XqF1 | ENSMUST00000033805 | NM_016883 | NM_016883 | Mm.17640 |
| Psmd11 | proteasome (prosome, macropain) 26S subunit, non-ATPase, 11 | Mus musculus proteasome (prosome, macropain) 26S subunit, non-ATPase, 11 (Psmd11), mRNA [NM_178616] | chr11 | + | mm\|11qB5 | ENSMUST00000172847 | NM_178616 | NM_178616 | Mm.260539 |
| Psmd14 | proteasome (prosome, macropain) 26S subunit, non-ATPase, 14 | Mus musculus proteasome (prosome, macropain) 26S subunit, non-ATPase, 14 (Psmd14), mRNA [NM_021526] | chr2 | + | mm\|2qC1.3 | ENSMUST00000146051 | NM_021526 | NM_021526 | Mm.218198 |
| Psmd2 | proteasome (prosome, macropain) 26S subunit, non-ATPase, 2 | Mus musculus proteasome (prosome, macropain) 26S subunit, non-ATPase, 2 (Psmd2), transcript variant 1, mRNA [NM_134101] | chr16 | + | mm\|16qB1 | ENSMUST00000007212 | NM_134101 | NM_134101 | Mm.243234 |
| Psmd3 | proteasome (prosome, macropain) 26S subunit, non-ATPase, 3 | Mus musculus proteasome (prosome, macropain) 26S subunit, non-ATPase, 3 (Psmd3), mRNA [NM_009439] | chr11 | + | mm\|11qD | ENSMUST00000017365 | NM_009439 | NM_009439 | Mm.12194 |
| Psme3 | proteaseome (prosome, macropain) activator subunit 3 (PA28 gamma, Ki) | Mus musculus proteaseome (prosome, macropain) activator subunit 3 (PA28 gamma, Ki) (Psme3), mRNA [NM_011192] | chr11 | + | mm\|11qD | ENSMUST00000019470 | NM_011192 | NM_011192 | Mm.288477 |
| Psmg1 | proteasome (prosome, macropain) assembly chaperone 1 | Mus musculus proteasome (prosome, macropain) assembly chaperone 1 (Psmg1), mRNA [NM_019537] | chr16 | - | mm\|16qC4 | ENSMUST00000023630 | NM_019537 | NM_019537 | Mm.396248 |
| Psmg4 | proteasome (prosome, macropain) assembly chaperone 4 | Mus musculus proteasome (prosome, macropain) assembly chaperone 4 (Psmg4), transcript variant 2, mRNA [NM_001101430] | chr13 | + | mm\|13qA3.3 | ENSMUST00000124996 | NM_001101430 | NM_001101430 | Mm.24788 |
| Psph | phosphoserine phosphatase | Mus musculus phosphoserine phosphatase (Psph), mRNA [NM_133900] | chr5 | - | mm\|5qG1.3 | ENSMUST00000031399 | NM_133900 | NM_133900 | Mm.271784 |
| Ptges3 | prostaglandin E synthase 3 | Mus musculus prostaglandin E synthase 3 (cytosolic) (Ptges3), mRNA [NM_019766] | chr10 | + | mm\|10qD3 | ENSMUST00000084771 | NM_019766 | NM_019766 | Mm.305816 |
| Ptk2 | PTK2 protein tyrosine kinase 2 | Mus musculus PTK2 protein tyrosine kinase 2 (Ptk2), transcript variant 2, mRNA [NM_001130409] | chr15 | - | mm\|15qD3 | ENSMUST00000170939 | NM_001130409 | NM_001130409 | Mm.254494 |
| Ptp4a2 | protein tyrosine phosphatase 4a2 | Mus musculus protein tyrosine phosphatase 4a2 (Ptp4a2), transcript variant 1, mRNA [NM_008974] | chr4 | + | mm\|4qD2.2 | ENSMUST00000030578 | NM_008974 | NM_008974 | Mm.193688 |
| Pwp2 | PWP2 periodic tryptophan protein homolog (yeast) | Mus musculus PWP2 periodic tryptophan protein homolog (yeast) (Pwp2), mRNA [NM_029546] | chr10 | - | mm\|10qC1 | ENSMUST00000042556 | NM_029546 | NM_029546 | Mm.103522 |
| Pyroxd2 | pyridine nucleotide-disulphide oxidoreductase domain 2 | Mus musculus pyridine nucleotide-disulphide oxidoreductase domain 2 (Pyroxd2), mRNA [NM_029011] | chr19 | - | mm\|19qC3 | ENSMUST00000076505 | NM_029011 | NM_029011 | Mm.329858 |
| R3hdm2 | R3H domain containing 2 | Mus musculus R3H domain containing 2 (R3hdm2), transcript variant 3, mRNA [NM_001168293] | chr10 | + | mm\|10qD3 | ENSMUST00000166820 | NM_001168293 | NM_001168293 | Mm.29342 |
| Rab1b | RAB1B, member RAS oncogene family | Mus musculus RAB1B, member RAS oncogene family (Rab1b), mRNA [NM_029576] | chr19 | - | mm\|19qA | ENSMUST00000025804 | NM_029576 | NM_029576 | Mm.182563 |
| Rad23a | RAD23 homolog A, nucleotide excision repair protein | Mus musculus RAD23 homolog A, nucleotide excision repair protein (Rad23a), transcript variant 1, mRNA [NM_001297606] | chr8 | - | mm\|8qC3 | ENSMUST00000003911 | NM_001297606 | NM_001297606 | Mm.255539 |
| Rad23b | RAD23 homolog B, nucleotide excision repair protein | Mus musculus RAD23 homolog B, nucleotide excision repair protein (Rad23b), mRNA [NM_009011] | chr4 | + | mm\|4qB3 | ENSMUST00000030134 | NM_009011 | NM_009011 | Mm.196846 |
| Ran | RAN, member RAS oncogene family | Mus musculus RAN, member RAS oncogene family (Ran), mRNA [NM_009391] | chr5 | + | mm\|5qG1.3 | ENSMUST00000031383 | NM_009391 | NM_009391 | Mm.297440 |
| Rap1b | RAS related protein 1b | RAS related protein 1b [Source:MGI Symbol;Acc:MGI:894315] [ENSMUST00000064667] | chr10 | - | mm\|10qD2 | ENSMUST00000064667 | BG242006 |  | Mm.392484 |
| Rassf8 | Ras association (RalGDS/AF-6) domain family (N-terminal) member 8 | Mus musculus Ras association (RalGDS/AF-6) domain family (N-terminal) member 8 (Rassf8), mRNA [NM_027760] | chr6 | + | mm\|6qG3 | ENSMUST00000032388 | NM_027760 | NM_027760 | Mm.444985 |
| Rbfa | ribosome binding factor A | Mus musculus ribosome binding factor A (Rbfa), mRNA [NM_199197] | chr18 | - | mm\|18qE3 | ENSMUST00000025462 | NM_199197 | NM_199197 | Mm.186936 |
| Rbm12 | RNA binding motif protein 12 | Mus musculus RNA binding motif protein 12 (Rbm12), transcript variant 1, mRNA [NM_029397] | chr2 | - | mm\|2qH1 | ENSMUST00000059647 | NM_029397 | NM_029397 | Mm.27660 |
| Rbm18 | RNA binding motif protein 18 | Mus musculus RNA binding motif protein 18 (Rbm18), transcript variant 1, mRNA [NM_026434] | chr2 | - | mm\|2qB | ENSMUST00000028251 | NM_026434 | NM_026434 | Mm.205937 |
| Rbm42 | RNA binding motif protein 42 | Mus musculus RNA binding motif protein 42 (Rbm42), mRNA [NM_133693] | chr7 | - | mm\|7qB1 | ENSMUST00000042726 | NM_133693 | NM_133693 | Mm.401747 |
| Rcan3 | regulator of calcineurin 3 | Mus musculus regulator of calcineurin 3 (Rcan3), mRNA [NM_022980] | chr4 | - | mm\|4qD3 | ENSMUST00000030606 | NM_022980 | NM_022980 | Mm.331970 |
| Rer1 | retention in endoplasmic reticulum sorting receptor 1 | Mus musculus retention in endoplasmic reticulum sorting receptor 1 (Rer1), mRNA [NM_026395] | chr4 | - | mm\|4qE2 | ENSMUST00000149796 | NM_026395 | NM_026395 | Mm.241604 |
| Rhbdd1 | rhomboid domain containing 1 | Mus musculus rhomboid domain containing 1 (Rhbdd1), transcript variant 1, mRNA [NM_029777] | chr1 | + | mm\|1qC5 | ENSMUST00000140020 | NM_029777 | NM_029777 | Mm.226994 |
| Rin1 | Ras and Rab interactor 1 | Mus musculus Ras and Rab interactor 1 (Rin1), mRNA [NM_145495] | chr19 | + | mm\|19qA | ENSMUST00000025818 | NM_145495 | NM_145495 | Mm.271922 |
| Rlim | ring finger protein, LIM domain interacting | Mus musculus ring finger protein, LIM domain interacting (Rlim), mRNA [NM_011276] | chrX | - | mm\|XqD | ENSMUST00000121153 | NM_011276 | NM_011276 | Mm.427762 |
| Rmnd5b | required for meiotic nuclear division 5 homolog B | Mus musculus required for meiotic nuclear division 5 homolog B (Rmnd5b), mRNA [NM_025346] | chr11 | - | mm\|11qB1.3 | ENSMUST00000147009 | NM_025346 | NM_025346 | Mm.288183 |
| Rnf187 | ring finger protein 187 | Mus musculus ring finger protein 187 (Rnf187), mRNA [NM_022423] | chr11 | - | mm\|11qB1.3 | ENSMUST00000094151 | NM_022423 | NM_022423 | Mm.249986 |
| Rnf25 | ring finger protein 25 | Mus musculus ring finger protein 25 (Rnf25), transcript variant 1, mRNA [NM_021313] | chr1 | - | mm\|1qC3 | ENSMUST00000113721 | NM_021313 | NM_021313 | Mm.280920 |
| Rnf31 | ring finger protein 31 | Mus musculus ring finger protein 31 (Rnf31), mRNA [NM_194346] | chr14 | + | mm\|14qC3 | ENSMUST00000140178 | NM_194346 | NM_194346 | Mm.23748 |
| Rnf4 | ring finger protein 4 | Mus musculus ring finger protein 4 (Rnf4), transcript variant 1, mRNA [NM_011278] | chr5 | + | mm\|5qB2 | ENSMUST00000182047 | NM_011278 | NM_011278 | Mm.21281 |
| Rpap3 | RNA polymerase II associated protein 3 | Mus musculus RNA polymerase II associated protein 3 (Rpap3), mRNA [NM_028003] | chr15 | - | mm\|15qF1 | ENSMUST00000023104 | NM_028003 | NM_028003 | Mm.12255 |
| Rpe | ribulose-5-phosphate-3-epimerase | Mus musculus ribulose-5-phosphate-3-epimerase (Rpe), transcript variant 3, mRNA [NM_001310643] | chr1 | + | mm\|1qC3 | ENSMUST00000142920 | NM_001310643 | NM_001310643 | Mm.240912 |
| Rpf2 | ribosome production factor 2 homolog | Mus musculus ribosome production factor 2 homolog (Rpf2), transcript variant 2, mRNA [NM_001042556] | chr10 | - | mm\|10qB1 | ENSMUST00000182287 | NM_001042556 | NM_001042556 | Mm.11480 |
| Rpl12 | ribosomal protein L12 | Mus musculus ribosomal protein L12 (Rpl12), mRNA [NM_009076] | chr2 | + | mm\|2qB | ENSMUST00000092620 | NM_009076 | NM_009076 | Mm.250030 |
| Rpl14 | ribosomal protein L14 |  | chr9 | + | mm\|9qF4 |  | BC013249 |  | Mm.289810 |
| Rpl21-ps8 |  |  |  |  |  |  |  |  |  |
| Rpl27-ps3 |  |  |  |  |  |  |  |  |  |
| Rpl31-ps16 |  |  |  |  |  |  |  |  |  |
| Rpl36al | ribosomal protein L36A-like | Mus musculus ribosomal protein L36A-like (Rpl36al), mRNA [NM_025589] | chr12 | - | mm\|12qC2 | ENSMUST00000054544 | NM_025589 | NM_025589 | Mm.491116 |
| Rpl36a-ps3 |  |  |  |  |  |  |  |  |  |
| Rpl37a | ribosomal protein L37a | Mus musculus ribosomal protein L37a (Rpl37a), mRNA [NM_009084] | chr1 | + | mm\|1qC3 | ENSMUST00000059980 | NM_009084 | NM_009084 | Mm.21529 |
| Rpl7a | ribosomal protein L7A | Mus musculus ribosomal protein L7A (Rpl7a), mRNA [NM_013721] | chr2 | + | mm\|2qA3 | ENSMUST00000137376 | NM_013721 | NM_013721 | Mm.398371 |
| Rpl9-ps7 |  |  |  |  |  |  |  |  |  |
| Rplp2-ps1 |  |  |  |  |  |  |  |  |  |
| Rps12l1 |  |  |  |  |  |  |  |  |  |
| Rps12-ps3 |  |  |  |  |  |  |  |  |  |
| Rps12-ps9 |  |  |  |  |  |  |  |  |  |
| Rps24-ps3 |  |  |  |  |  |  |  |  |  |
| Rps27a | ribosomal protein S27A | Mus musculus ribosomal protein S27A (Rps27a), transcript variant 2, mRNA [NM_001033865] | chr11 | - | mm\|11qA3.3 | ENSMUST00000181799 | NM_001033865 | NM_001033865 | Mm.180003 |
| Rps2-ps10 |  |  |  |  |  |  |  |  |  |
| Rps2-ps13 |  |  |  |  |  |  |  |  |  |
| Rps2-ps6 |  |  |  |  |  |  |  |  |  |
| Rps2-ps8 |  |  |  |  |  |  |  |  |  |
| Rps9 | ribosomal protein S9 | Mus musculus ribosomal protein S9 (Rps9), mRNA [NM_029767] | chr7 | + | mm\|7qA1 | ENSMUST00000108624 | NM_029767 | NM_029767 | Mm.13944 |
| Rpsa-ps7 |  |  |  |  |  |  |  |  |  |
| Rrn3 | RRN3 RNA polymerase I transcription factor homolog (yeast) | Mus musculus RRN3 RNA polymerase I transcription factor homolog (yeast) (Rrn3), mRNA [NM_001039521] | chr16 | + | mm\|16qA1 | ENSMUST00000023363 | NM_001039521 | NM_001039521 | Mm.490362 |
| Rrp7a | ribosomal RNA processing 7 homolog A (S. cerevisiae) | ribosomal RNA processing 7 homolog A (S. cerevisiae) [Source:MGI Symbol;Acc:MGI:1922028] [ENSMUST00000018184] | chr15 | - | mm\|15qE1 | ENSMUST00000018184 | AK032596 |  | Mm.452636 |
| Rsl1d1 | ribosomal L1 domain containing 1 | Mus musculus ribosomal L1 domain containing 1 (Rsl1d1), mRNA [NM_025546] | chr16 | - | mm\|16qA1 | ENSMUST00000119953 | NM_025546 | NM_025546 | Mm.28291 |
| Rsl24d1 | ribosomal L24 domain containing 1 |  | chr9 | + | mm\|9qD |  | BC003885 |  | Mm.379366 |
| Rufy4 | RUN and FYVE domain containing 4 | Mus musculus RUN and FYVE domain containing 4 (Rufy4), transcript variant 2, mRNA [NM_001034060] | chr1 | + | mm\|1qC3 | ENSMUST00000080167 | NM_001034060 | NM_001034060 | Mm.240447 |
| Ruvbl2 | RuvB-like protein 2 | Mus musculus RuvB-like protein 2 (Ruvbl2), mRNA [NM_011304] | chr7 | - | mm\|7qB4 | ENSMUST00000107771 | NM_011304 | NM_011304 | Mm.34410 |
| Rwdd1 | RWD domain containing 1 | Mus musculus RWD domain containing 1 (Rwdd1), mRNA [NM_025614] | chr10 | - | mm\|10qB1 | ENSMUST00000019917 | NM_025614 | NM_025614 | Mm.28528 |
| S100a10 | S100 calcium binding protein A10 (calpactin) | Mus musculus S100 calcium binding protein A10 (calpactin) (S100a10), mRNA [NM_009112] | chr3 | + | mm\|3qF2.1 | ENSMUST00000045756 | NM_009112 | NM_009112 | Mm.1 |
| S100a6 | S100 calcium binding protein A6 (calcyclin) | Mus musculus S100 calcium binding protein A6 (calcyclin) (S100a6), mRNA [NM_011313] | chr3 | + | mm\|3qF1 | ENSMUST00000001051 | NM_011313 | NM_011313 | Mm.100144 |
| Sacs | sacsin | sacsin [Source:MGI Symbol;Acc:MGI:1354724] [ENSMUST00000119943] | chr14 | + | mm\|14qD1 | ENSMUST00000119943 | XM_006519226 | XM_006519226 | Mm.440703 |
| Sar1a | secretion associated Ras related GTPase 1A | Mus musculus secretion associated Ras related GTPase 1A (Sar1a), mRNA [NM_009120] | chr10 | + | mm\|10qB4 | ENSMUST00000219506 | NM_009120 | NM_009120 | Mm.391794 |
| Sars | seryl-aminoacyl-tRNA synthetase | Mus musculus seryl-aminoacyl-tRNA synthetase (Sars), transcript variant 1, mRNA [NM_011319] | chr3 | - | mm\|3qF3 | ENSMUST00000102625 | NM_011319 | NM_011319 | Mm.28688 |
| Scaf11 | SR-related CTD-associated factor 11 | Mus musculus SR-related CTD-associated factor 11 (Scaf11), mRNA [NM_028148] | chr15 | - | mm\|15qF1 | ENSMUST00000047835 | NM_028148 | NM_028148 | Mm.324474 |
| Scoc | short coiled-coil protein | Mus musculus short coiled-coil protein (Scoc), transcript variant 2, mRNA [NM_019708] | chr8 | - | mm\|8qC2 | ENSMUST00000167525 | NM_019708 | NM_019708 | Mm.246911 |
| Sdc1 | syndecan 1 | Mus musculus syndecan 1 (Sdc1), mRNA [NM_011519] | chr12 | + | mm\|12qA1.1 | ENSMUST00000020911 | NM_011519 | NM_011519 | Mm.2580 |
| Sdf2 | stromal cell derived factor 2 | Mus musculus stromal cell derived factor 2 (Sdf2), mRNA [NM_009143] | chr11 | + | mm\|11qB5 | ENSMUST00000002133 | NM_009143 | NM_009143 | Mm.390372 |
| Sdsl | serine dehydratase-like | Mus musculus serine dehydratase-like (Sdsl), mRNA [NM_133902] | chr5 | - | mm\|5qF | ENSMUST00000031594 | NM_133902 | NM_133902 | Mm.5162 |
| Septin2 |  |  |  |  |  |  |  |  |  |
| Serac1 | serine active site containing 1 | Mus musculus serine active site containing 1 (Serac1), transcript variant 3, mRNA [NM_001111017] | chr17 | - | mm\|17qA1 |  | NM_001111017 | NM_001111017 | Mm.5548 |
| Sesn2 | sestrin 2 | Mus musculus sestrin 2 (Sesn2), mRNA [NM_144907] | chr4 | - | mm\|4qD2.3 | ENSMUST00000030724 | NM_144907 | NM_144907 | Mm.23608 |
| Set | SET nuclear oncogene | Mus musculus SET nuclear oncogene (Set), transcript variant 2, mRNA [NM_001204875] | chr2 | + | mm\|2qB | ENSMUST00000125824 | NM_001204875 | NM_001204875 | Mm.335942 |
| Sft2d2 | SFT2 domain containing 2 | Mus musculus SFT2 domain containing 2 (Sft2d2), mRNA [NM_145512] | chr1 | - | mm\|1qH2.3 | ENSMUST00000192185 | NM_145512 | NM_145512 | Mm.288369 |
| Sgtb | small glutamine-rich tetratricopeptide repeat (TPR)-containing, beta | Mus musculus small glutamine-rich tetratricopeptide repeat (TPR)-containing, beta (Sgtb), mRNA [NM_144838] | chr13 | + | mm\|13qD1 | ENSMUST00000044385 | NM_144838 | NM_144838 | Mm.44416 |
| Sh3bp1 | SH3-domain binding protein 1 | Mus musculus SH3-domain binding protein 1 (Sh3bp1), transcript variant 2, mRNA [NM_009164] | chr15 | + | mm\|15qE1 | ENSMUST00000061239 | NM_009164 | NM_009164 | Mm.4462 |
| Sh3glb2 | SH3-domain GRB2-like endophilin B2 | Mus musculus SH3-domain GRB2-like endophilin B2 (Sh3glb2), transcript variant 4, mRNA [NM_001346806] | chr2 | - | mm\|2qB | ENSMUST00000113621 | NM_001346806 | NM_001346806 | Mm.33343 |
| Siva1 | SIVA1, apoptosis-inducing factor | Mus musculus SIVA1, apoptosis-inducing factor (Siva1), transcript variant 1, mRNA [NM_013929] | chr12 | + | mm\|12qF1 | ENSMUST00000077997 | NM_013929 | NM_013929 | Mm.289812 |
| Slc20a1 | solute carrier family 20, member 1 | Mus musculus solute carrier family 20, member 1 (Slc20a1), transcript variant 1, mRNA [NM_015747] | chr2 | + | mm\|2qF1 | ENSMUST00000110315 | NM_015747 | NM_015747 | Mm.272675 |
| Slc25a33 | solute carrier family 25, member 33 | Mus musculus solute carrier family 25, member 33 (Slc25a33), mRNA [NM_027460] | chr4 | - | mm\|4qE2 | ENSMUST00000105686 | NM_027460 | NM_027460 | Mm.41877 |
| Slc25a5 | solute carrier family 25 (mitochondrial carrier, adenine nucleotide translocator), member 5 | Mus musculus solute carrier family 25 (mitochondrial carrier, adenine nucleotide translocator), member 5 (Slc25a5), mRNA [NM_007451] | chrX | + | mm\|XqA3.3 |  | NM_007451 | NM_007451 | Mm.371544 |
| Slc25a51 | solute carrier family 25, member 51 | Mus musculus solute carrier family 25, member 51 (Slc25a51), mRNA [NM_001009949] | chr4 | - | mm\|4qB1 | ENSMUST00000116341 | NM_001009949 | NM_001009949 | Mm.260210 |
| Slc35b1 | solute carrier family 35, member B1 | Mus musculus solute carrier family 35, member B1 (Slc35b1), mRNA [NM_016752] | chr11 | + | mm\|11qD | ENSMUST00000021243 | NM_016752 | NM_016752 | Mm.4593 |
| Slc39a11 | solute carrier family 39 (metal ion transporter), member 11 | solute carrier family 39 (metal ion transporter), member 11 [Source:MGI Symbol;Acc:MGI:1917056] [ENSMUST00000042657] | chr11 | - | mm\|11qE2 | ENSMUST00000042657 | XM_011249241 | XM_011249241 |  |
| Slc3a2 | solute carrier family 3 (activators of dibasic and neutral amino acid transport), member 2 | Mus musculus solute carrier family 3 (activators of dibasic and neutral amino acid transport), member 2 (Slc3a2), transcript variant 2, mRNA [NM_008577] | chr19 | - | mm\|19qA | ENSMUST00000010239 | NM_008577 | NM_008577 | Mm.4114 |
| Smap1 | small ArfGAP 1 | Mus musculus small ArfGAP 1 (Smap1), transcript variant 1, mRNA [NM_028534] | chr1 | - | mm\|1qA5 | ENSMUST00000027339 | NM_028534 | NM_028534 | Mm.329963 |
| Smap2 | small ArfGAP 2 | Mus musculus small ArfGAP 2 (Smap2), mRNA [NM_133716] | chr4 | - | mm\|4qD2.2 | ENSMUST00000043200 | NM_133716 | NM_133716 | Mm.271819 |
| Smarcal1 | SWI/SNF related matrix associated, actin dependent regulator of chromatin, subfamily a-like 1 | Mus musculus SWI/SNF related matrix associated, actin dependent regulator of chromatin, subfamily a-like 1 (Smarcal1), mRNA [NM_018817] | chr1 | + | mm\|1qC3 | ENSMUST00000047615 | NM_018817 | NM_018817 | Mm.274232 |
| Smim26 | small integral membrane protein 26 | Mus musculus predicted gene 561 (Gm561), mRNA [NM_001033297] | chr2 | + | mm\|2qG1 | ENSMUST00000136628 | NM_001033297 | NM_001033297 | Mm.27391 |
| Smyd2 | SET and MYND domain containing 2 | Mus musculus SET and MYND domain containing 2 (Smyd2), mRNA [NM_026796] | chr1 | - | mm\|1qH6 | ENSMUST00000027897 | NM_026796 | NM_026796 | Mm.156895 |
| Snai2 | snail family zinc finger 2 | Mus musculus snail family zinc finger 2 (Snai2), mRNA [NM_011415] | chr16 | + | mm\|16qA1 | ENSMUST00000023356 | NM_011415 | NM_011415 | Mm.4272 |
| Snhg6 | small nucleolar RNA host gene 6 | Mus musculus small nucleolar RNA host gene 6 (Snhg6), long non-coding RNA [NR_024067] | chr1 | - | mm\|1qA2 |  | NR_024067 | NR_024067 | Mm.28311 |
| Snhg8 | small nucleolar RNA host gene 8 | AGENCOURT_10790207 NIH_MGC_154 Mus musculus cDNA clone IMAGE:30120368 5', mRNA sequence [CA481153] | chr3 | - | mm\|3qG1 |  | CA481153 |  | Mm.260791 |
| Snora74a | small nucleolar RNA, H/ACA box 74A | Mus musculus small nucleolar RNA, H/ACA box 74A (Snora74a), small nucleolar RNA [NR_002905] | chr18 | + | mm\|18qB2 |  | NR_002905 | NR_002905 | Mm.482118 |
| Snord3a |  |  |  |  |  |  |  |  |  |
| Snord94 |  |  |  |  |  |  |  |  |  |
| Snrnp25 | small nuclear ribonucleoprotein 25 (U11/U12) | Mus musculus small nuclear ribonucleoprotein 25 (U11/U12) (Snrnp25), mRNA [NM_030093] | chr11 | + | mm\|11qA4 | ENSMUST00000039601 | NM_030093 | NM_030093 | Mm.29952 |
| Snrnp40 | small nuclear ribonucleoprotein 40 (U5) | Mus musculus small nuclear ribonucleoprotein 40 (U5) (Snrnp40), mRNA [NM_025645] | chr4 | + | mm\|4qD2.2 | ENSMUST00000105994 | NM_025645 | NM_025645 | Mm.423019 |
| Snrpd2 | small nuclear ribonucleoprotein D2 | Mus musculus small nuclear ribonucleoprotein D2 (Snrpd2), mRNA [NM_026943] | chr7 | + | mm\|7qA3 | ENSMUST00000049294 | NM_026943 | NM_026943 | Mm.29135 |
| Snrpg | small nuclear ribonucleoprotein polypeptide G | Mus musculus small nuclear ribonucleoprotein polypeptide G (Snrpg), mRNA [NM_026506] | chr6 | + | mm\|6qD1 | ENSMUST00000204768 | NM_026506 | NM_026506 | Mm.276802 |
| Snx33 | sorting nexin 33 | Mus musculus sorting nexin 33 (Snx33), mRNA [NM_175483] | chr9 | - | mm\|9qB | ENSMUST00000050916 | NM_175483 | NM_175483 | Mm.191459 |
| Sos1 | son of sevenless homolog 1 (Drosophila) | Mus musculus son of sevenless homolog 1 (Drosophila) (Sos1), mRNA [NM_009231] | chr17 | - | mm\|17qE3 | ENSMUST00000068714 | NM_009231 | NM_009231 | Mm.360004 |
| Spout1 | SPOUT domain containing methyltransferase 1 | Mus musculus SPOUT domain containing methyltransferase 1 (Spout1), mRNA [NM_172660] | chr2 | - | mm\|2qB | ENSMUST00000144093 | NM_172660 | NM_172660 | Mm.4449 |
| Sqstm1 | sequestosome 1 | sequestosome 1 [Source:MGI Symbol;Acc:MGI:107931] [ENSMUST00000015981] | chr11 | - | mm\|11qB1.3 | ENSMUST00000015981 | AK028898 |  |  |
| Srbd1 | S1 RNA binding domain 1 | Mus musculus S1 RNA binding domain 1 (Srbd1), mRNA [NM_030133] | chr17 | - | mm\|17qE4 | ENSMUST00000095187 | NM_030133 | NM_030133 | Mm.146236 |
| Src | Rous sarcoma oncogene | Mus musculus Rous sarcoma oncogene (Src), transcript variant 1, mRNA [NM_009271] | chr2 | + | mm\|2qH1 | ENSMUST00000109529 | NM_009271 | NM_009271 | Mm.22845 |
| Srp68 | signal recognition particle 68 | Mus musculus signal recognition particle 68 (Srp68), mRNA [NM_146032] | chr11 | - | mm\|11qE2 | ENSMUST00000021133 | NM_146032 | NM_146032 | Mm.29655 |
| Srr | serine racemase | Mus musculus serine racemase (Srr), transcript variant 1, mRNA [NM_013761] | chr11 | - | mm\|11qB5 | ENSMUST00000138612 | NM_013761 | NM_013761 | Mm.131443 |
| Srxn1 | sulfiredoxin 1 homolog (S. cerevisiae) | Mus musculus sulfiredoxin 1 homolog (S. cerevisiae) (Srxn1), mRNA [NM_029688] | chr2 | + | mm\|2qG3 | ENSMUST00000041500 | NM_029688 | NM_029688 | Mm.218639 |
| Ssr1 | signal sequence receptor, alpha | Mus musculus signal sequence receptor, alpha (Ssr1), mRNA [NM_025965] | chr13 | - | mm\|13qA3.3 | ENSMUST00000021864 | NM_025965 | NM_025965 | Mm.490298 |
| Ssu72 | Ssu72 RNA polymerase II CTD phosphatase homolog (yeast) | Mus musculus Ssu72 RNA polymerase II CTD phosphatase homolog (yeast) (Ssu72), mRNA [NM_026899] | chr4 | + | mm\|4qE2 | ENSMUST00000030905 | NM_026899 | NM_026899 | Mm.294770 |
| Stam | signal transducing adaptor molecule (SH3 domain and ITAM motif) 1 | Mus musculus signal transducing adaptor molecule (SH3 domain and ITAM motif) 1 (Stam), mRNA [NM_011484] | chr2 | + | mm\|2qA2 | ENSMUST00000102960 | NM_011484 | NM_011484 | Mm.273174 |
| Stambpl1 | STAM binding protein like 1 | Mus musculus STAM binding protein like 1 (Stambpl1), mRNA [NM_029682] | chr19 | + | mm\|19qC1 | ENSMUST00000119603 | NM_029682 | NM_029682 | Mm.130952 |
| Stau2 | staufen (RNA binding protein) homolog 2 (Drosophila) | Mus musculus staufen (RNA binding protein) homolog 2 (Drosophila) (Stau2), transcript variant 3, mRNA [NM_025303] | chr1 | - | mm\|1qA3 | ENSMUST00000149320 | NM_025303 | NM_025303 | Mm.216257 |
| Steap1 | six transmembrane epithelial antigen of the prostate 1 | Mus musculus six transmembrane epithelial antigen of the prostate 1 (Steap1), mRNA [NM_027399] | chr5 | - | mm\|5qA1 | ENSMUST00000169542 | NM_027399 | NM_027399 | Mm.85429 |
| Stip1 | stress-induced phosphoprotein 1 | Mus musculus stress-induced phosphoprotein 1 (Stip1), mRNA [NM_016737] | chr19 | - | mm\|19qA | ENSMUST00000025918 | NM_016737 | NM_016737 | Mm.258633 |
| Stk19 | serine/threonine kinase 19 | Mus musculus serine/threonine kinase 19 (Stk19), mRNA [NM_019442] | chr17 | - | mm\|17qB1 | ENSMUST00000159333 | NM_019442 | NM_019442 | Mm.440064 |
| Strap | serine/threonine kinase receptor associated protein | Mus musculus serine/threonine kinase receptor associated protein (Strap), mRNA [NM_011499] | chr6 | + | mm\|6qG1 | ENSMUST00000137235 | NM_011499 | NM_011499 | Mm.22584 |
| Stub1 | STIP1 homology and U-Box containing protein 1 | Mus musculus STIP1 homology and U-Box containing protein 1 (Stub1), mRNA [NM_019719] | chr17 | - | mm\|17qA3.3 | ENSMUST00000044911 | NM_019719 | NM_019719 | Mm.491120 |
| Stxbp3 | syntaxin binding protein 3 | Mus musculus syntaxin binding protein 3 (Stxbp3), mRNA [NM_011504] | chr3 | - | mm\|3qF3 | ENSMUST00000106596 | NM_011504 | NM_011504 | Mm.316894 |
| Swsap1 | SWIM type zinc finger 7 associated protein 1 | Mus musculus SWIM type zinc finger 7 associated protein 1 (Swsap1), mRNA [NM_025870] | chr9 | + | mm\|9qA3 | ENSMUST00000053583 | NM_025870 | NM_025870 | Mm.46647 |
| Taf15 | TATA-box binding protein associated factor 15 | Mus musculus TATA-box binding protein associated factor 15 (Taf15), mRNA [NM_027427] | chr11 | + | mm\|11qC | ENSMUST00000021018 | NM_027427 | NM_027427 | Mm.181050 |
| Tagln2 | transgelin 2 | Mus musculus transgelin 2 (Tagln2), mRNA [NM_178598] | chr1 | + | mm\|1qH3 | ENSMUST00000111228 | NM_178598 | NM_178598 | Mm.271711 |
| Tank | TRAF family member-associated Nf-kappa B activator | Mus musculus TRAF family member-associated Nf-kappa B activator (Tank), transcript variant 3, mRNA [NM_011529] | chr2 | + | mm\|2qC1.3 | ENSMUST00000145632 | NM_011529 | NM_011529 | Mm.244393 |
| Tars | threonyl-tRNA synthetase | Mus musculus threonyl-tRNA synthetase (Tars), mRNA [NM_033074] | chr15 | - | mm\|15qA1 | ENSMUST00000022849 | NM_033074 | NM_033074 | Mm.286061 |
| Tbc1d31 | TBC1 domain family, member 31 | TBC1 domain family, member 31 [Source:MGI Symbol;Acc:MGI:2684931] [ENSMUST00000161329] | chr15 | + | mm\|15qD1 | ENSMUST00000161329 | XM_006520698 | XM_006520698 |  |
| Tbl1x | transducin (beta)-like 1 X-linked | Mus musculus transducin (beta)-like 1 X-linked (Tbl1x), mRNA [NM_020601] | chrX | + | mm\|XqA7.3 | ENSMUST00000088217 | NM_020601 | NM_020601 | Mm.258476 |
| Tcea1 | transcription elongation factor A (SII) 1 | Mus musculus transcription elongation factor A (SII) 1 (Tcea1), transcript variant 2, mRNA [NM_011541] | chr1 | + | mm\|1qA1 | ENSMUST00000081551 | NM_011541 | NM_011541 | Mm.207263 |
| Tex30 | testis expressed 30 | Mus musculus testis expressed 30 (Tex30), mRNA [NM_029368] | chr1 | - | mm\|1qC1.1 | ENSMUST00000147571 | NM_029368 | NM_029368 | Mm.348017 |
| Thnsl1 | threonine synthase-like 1 (bacterial) | Mus musculus threonine synthase-like 1 (bacterial) (Thnsl1), transcript variant 1, mRNA [NM_177588] | chr2 | + | mm\|2qA3 | ENSMUST00000054591 | NM_177588 | NM_177588 | Mm.268841 |
| Tmco1 | transmembrane and coiled-coil domains 1 | Mus musculus transmembrane and coiled-coil domains 1 (Tmco1), mRNA [NM_001039483] | chr1 | + | mm\|1qH2.3 | ENSMUST00000195015 | NM_001039483 | NM_001039483 | Mm.330045 |
| Tmed2 | transmembrane p24 trafficking protein 2 | Mus musculus transmembrane p24 trafficking protein 2 (Tmed2), mRNA [NM_019770] | chr5 | + | mm\|5qF | ENSMUST00000135464 | NM_019770 | NM_019770 | Mm.425279 |
| Tmem123 | transmembrane protein 123 | Mus musculus transmembrane protein 123 (Tmem123), mRNA [NM_133739] | chr9 | + | mm\|9qA1 | ENSMUST00000154371 | NM_133739 | NM_133739 | Mm.283293 |
| Tmem14c | transmembrane protein 14C | Mus musculus transmembrane protein 14C (Tmem14c), mRNA [NM_025387] | chr13 | + | mm\|13qA3.3 | ENSMUST00000021790 | NM_025387 | NM_025387 | Mm.30005 |
| Tmem150a | transmembrane protein 150A | Mus musculus transmembrane protein 150A (Tmem150a), mRNA [NM_144916] | chr6 | + | mm\|6qC1 | ENSMUST00000069695 | NM_144916 | NM_144916 | Mm.491165 |
| Tmem18 | transmembrane protein 18 | Mus musculus transmembrane protein 18 (Tmem18), mRNA [NM_172049] | chr12 | + | mm\|12qA2 | ENSMUST00000057151 | NM_172049 | NM_172049 | Mm.34035 |
| Tmem223 | transmembrane protein 223 | Mus musculus transmembrane protein 223 (Tmem223), mRNA [NM_025791] | chr19 | + | mm\|19qA | ENSMUST00000184970 | NM_025791 | NM_025791 | Mm.440071 |
| Tmem237 | transmembrane protein 237 | Mus musculus transmembrane protein 237 (Tmem237), transcript variant 2, mRNA [NM_001037812] | chr1 | - | mm\|1qC1.3 | ENSMUST00000087475 | NM_001037812 | NM_001037812 | Mm.261275 |
| Tmem8b | transmembrane protein 8B | Mus musculus transmembrane protein 8B (Tmem8b), mRNA [NM_001085508] | chr4 | + | mm\|4qB1 | ENSMUST00000107864 | NM_001085508 | NM_001085508 | Mm.171489 |
| Tmsb10 | thymosin, beta 10 |  | chr6 | - | mm\|6qC1 |  | AK008557 |  | Mm.3532 |
| Tmx2 | thioredoxin-related transmembrane protein 2 | Mus musculus thioredoxin-related transmembrane protein 2 (Tmx2), transcript variant 1, mRNA [NM_025868] | chr2 | - | mm\|2qD | ENSMUST00000053664 | NM_025868 | NM_025868 | Mm.371621 |
| Tnfaip1 | tumor necrosis factor, alpha-induced protein 1 (endothelial) | Mus musculus tumor necrosis factor, alpha-induced protein 1 (endothelial) (Tnfaip1), transcript variant 1, mRNA [NM_009395] | chr11 | - | mm\|11qB5 | ENSMUST00000108277 | NM_009395 | NM_009395 | Mm.386774 |
| Tnfaip2 | tumor necrosis factor, alpha-induced protein 2 | Mus musculus tumor necrosis factor, alpha-induced protein 2 (Tnfaip2), mRNA [NM_009396] | chr12 | + | mm\|12qF1 | ENSMUST00000102745 | NM_009396 | NM_009396 | Mm.255332 |
| Tnfsf13 |  |  |  |  |  |  |  |  |  |
| Tomm20 | translocase of outer mitochondrial membrane 20 homolog (yeast) | Mus musculus translocase of outer mitochondrial membrane 20 homolog (yeast) (Tomm20), mRNA [NM_024214] | chr8 | - | mm\|8qE2 | ENSMUST00000179857 | NM_024214 | NM_024214 | Mm.372086 |
| Tomm22 | translocase of outer mitochondrial membrane 22 homolog (yeast) | Mus musculus translocase of outer mitochondrial membrane 22 homolog (yeast) (Tomm22), mRNA [NM_172609] | chr15 | + | mm\|15qE1 | ENSMUST00000023062 | NM_172609 | NM_172609 | Mm.485795 |
| Trap1 | TNF receptor-associated protein 1 | Mus musculus TNF receptor-associated protein 1 (Trap1), mRNA [NM_026508] | chr16 | - | mm\|16qA1 | ENSMUST00000006137 | NM_026508 | NM_026508 | Mm.123366 |
| Trappc10 | trafficking protein particle complex 10 | Mus musculus trafficking protein particle complex 10 (Trappc10), mRNA [NM_001081055] | chr10 | - | mm\|10qC1 | ENSMUST00000000384 | NM_001081055 | NM_001081055 | Mm.27539 |
| Trappc3 | trafficking protein particle complex 3 | Mus musculus trafficking protein particle complex 3 (Trappc3), mRNA [NM_013718] | chr4 | + | mm\|4qD2.2 | ENSMUST00000030660 | NM_013718 | NM_013718 | Mm.8392 |
| Trib3 | tribbles pseudokinase 3 | Mus musculus tribbles pseudokinase 3 (Trib3), mRNA [NM_175093] | chr2 | - | mm\|2qG3 | ENSMUST00000040312 | NM_175093 | NM_175093 | Mm.276018 |
| Trip10 | thyroid hormone receptor interactor 10 | Mus musculus thyroid hormone receptor interactor 10 (Trip10), transcript variant 1, mRNA [NM_001242389] | chr17 | + | mm\|17qD | ENSMUST00000019631 | NM_001242389 | NM_001242389 | Mm.37368 |
| Trit1 | tRNA isopentenyltransferase 1 | Mus musculus tRNA isopentenyltransferase 1 (Trit1), mRNA [NM_025873] | chr4 | + | mm\|4qD2.2 | ENSMUST00000141855 | NM_025873 | NM_025873 | Mm.235030 |
| Trmt10b | tRNA methyltransferase 10B | Mus musculus tRNA methyltransferase 10B (Trmt10b), mRNA [NM_027266] | chr4 | + | mm\|4qB1 | ENSMUST00000044673 | NM_027266 | NM_027266 | Mm.46761 |
| Tsku | tsukushi, small leucine rich proteoglycan | Mus musculus tsukushi, small leucine rich proteoglycan (Tsku), transcript variant 3, mRNA [NM_001024619] | chr7 | - | mm\|7qE2 | ENSMUST00000206414 | NM_001024619 | NM_001024619 | Mm.25317 |
| Tsr3 | TSR3 20S rRNA accumulation | Mus musculus TSR3 20S rRNA accumulation (Tsr3), transcript variant 1, mRNA [NM_026676] | chr17 | + | mm\|17qA3.3 | ENSMUST00000063574 | NM_026676 | NM_026676 | Mm.180687 |
| Tuba1b | tubulin, alpha 1B | Mus musculus tubulin, alpha 1B (Tuba1b), mRNA [NM_011654] | chr15 | - | mm\|15qF1 | ENSMUST00000077577 | NM_011654 | NM_011654 | Mm.392113 |
| Tuba1c | tubulin, alpha 1C | Mus musculus tubulin, alpha 1C (Tuba1c), mRNA [NM_009448] | chr15 | + | mm\|15qF1 | ENSMUST00000058914 | NM_009448 | NM_009448 | Mm.88212 |
| Tubb6 | tubulin, beta 6 class V | Mus musculus tubulin, beta 6 class V (Tubb6), mRNA [NM_026473] | chr18 | + | mm\|18qE1 | ENSMUST00000001513 | NM_026473 | NM_026473 | Mm.181860 |
| Tufm | Tu translation elongation factor, mitochondrial |  | chr7 | + | mm\|7qF3 |  | BC100596 |  | Mm.197829 |
| Twf2 | twinfilin actin binding protein 2 | Mus musculus twinfilin actin binding protein 2 (Twf2), mRNA [NM_011876] | chr9 | + | mm\|9qF1 | ENSMUST00000217523 | NM_011876 | NM_011876 | Mm.274346 |
| Txn1 | thioredoxin 1 | Mus musculus thioredoxin 1 (Txn1), mRNA [NM_011660] | chr4 | - | mm\|4qB3 | ENSMUST00000030051 | NM_011660 | NM_011660 | Mm.260618 |
| Txndc12 | thioredoxin domain containing 12 (endoplasmic reticulum) | Mus musculus thioredoxin domain containing 12 (endoplasmic reticulum) (Txndc12), mRNA [NM_025334] | chr4 | + | mm\|4qC7 | ENSMUST00000030296 | NM_025334 | NM_025334 | Mm.159965 |
| Tyw5 | tRNA-yW synthesizing protein 5 | Mus musculus tRNA-yW synthesizing protein 5 (Tyw5), transcript variant 5, mRNA [NM_001302963] | chr1 | - | mm\|1qC1.3 | ENSMUST00000079998 | NM_001302963 | NM_001302963 | Mm.288490 |
| Uba1 | ubiquitin-like modifier activating enzyme 1 | Mus musculus ubiquitin-like modifier activating enzyme 1 (Uba1), transcript variant 3, mRNA [NM_001276316] | chrX | + | mm\|XqA1.3 | ENSMUST00000089217 | NM_001276316 | NM_001276316 | Mm.1104 |
| Ube2f | ubiquitin-conjugating enzyme E2F (putative) | Mus musculus ubiquitin-conjugating enzyme E2F (putative) (Ube2f), mRNA [NM_026454] | chr1 | + | mm\|1qD | ENSMUST00000080066 | NM_026454 | NM_026454 | Mm.337238 |
| Ube2i | ubiquitin-conjugating enzyme E2I | Mus musculus ubiquitin-conjugating enzyme E2I (Ube2i), transcript variant 2, mRNA [NM_001177609] | chr17 | - | mm\|17qA3.3 | ENSMUST00000173084 | NM_001177609 | NM_001177609 | Mm.240044 |
| Ube2o | ubiquitin-conjugating enzyme E2O | Mus musculus ubiquitin-conjugating enzyme E2O (Ube2o), mRNA [NM_173755] | chr11 | - | mm\|11qE2 | ENSMUST00000082152 | NM_173755 | NM_173755 | Mm.243950 |
| Ubqln1 | ubiquilin 1 | Mus musculus ubiquilin 1 (Ubqln1), transcript variant 2, mRNA [NM_152234] | chr13 | - | mm\|13qB1 | ENSMUST00000058735 | NM_152234 | NM_152234 | Mm.182053 |
| Ubr7 | ubiquitin protein ligase E3 component n-recognin 7 (putative) | Mus musculus ubiquitin protein ligase E3 component n-recognin 7 (putative) (Ubr7), mRNA [NM_025666] | chr12 | + | mm\|12qE | ENSMUST00000046404 | NM_025666 | NM_025666 | Mm.34261 |
| Ubtd1 | ubiquitin domain containing 1 | Mus musculus ubiquitin domain containing 1 (Ubtd1), mRNA [NM_145500] | chr19 | + | mm\|19qC3 | ENSMUST00000026170 | NM_145500 | NM_145500 | Mm.22948 |
| Ufm1 | ubiquitin-fold modifier 1 | Mus musculus ubiquitin-fold modifier 1 (Ufm1), mRNA [NM_026435] | chr3 | - | mm\|3qC | ENSMUST00000146598 | NM_026435 | NM_026435 | Mm.23067 |
| Ugt1a6a | UDP glucuronosyltransferase 1 family, polypeptide A6A | Mus musculus UDP glucuronosyltransferase 1 family, polypeptide A6A (Ugt1a6a), mRNA [NM_145079] | chr1 | + | mm\|1qD | ENSMUST00000113135 | NM_145079 | NM_145079 | Mm.300095 |
| Upp1 | uridine phosphorylase 1 | Mus musculus uridine phosphorylase 1 (Upp1), transcript variant 1, mRNA [NM_009477] | chr11 | + | mm\|11qA1 | ENSMUST00000020677 | NM_009477 | NM_009477 | Mm.4610 |
| Uqcc1 | ubiquinol-cytochrome c reductase complex assembly factor 1 | Mus musculus ubiquinol-cytochrome c reductase complex assembly factor 1 (Uqcc1), transcript variant 1, mRNA [NM_018888] | chr2 | - | mm\|2qH1 | ENSMUST00000109631 | NM_018888 | NM_018888 | Mm.158827 |
| Uqcc2 | ubiquinol-cytochrome c reductase complex assembly factor 2 | Mus musculus ubiquinol-cytochrome c reductase complex assembly factor 2 (Uqcc2), mRNA [NM_026063] | chr17 | - | mm\|17qA3.3 | ENSMUST00000118613 | NM_026063 | NM_026063 | Mm.272389 |
| Uqcc3 | ubiquinol-cytochrome c reductase complex assembly factor 3 | Mus musculus ubiquinol-cytochrome c reductase complex assembly factor 3 (Uqcc3), mRNA [NM_001160356] | chr19 | - | mm\|19qA | ENSMUST00000096253 | NM_001160356 | NM_001160356 | Mm.381181 |
| Uqcr10 | ubiquinol-cytochrome c reductase, complex III subunit X | Mus musculus ubiquinol-cytochrome c reductase, complex III subunit X (Uqcr10), mRNA [NM_197979] | chr11 | - | mm\|11qA1 |  | NM_197979 | NM_197979 | Mm.269736 |
| Uqcrb | ubiquinol-cytochrome c reductase binding protein | Mus musculus ubiquinol-cytochrome c reductase binding protein (Uqcrb), mRNA [NM_026219] | chr13 | - | mm\|13qB3 | ENSMUST00000021993 | NM_026219 | NM_026219 | Mm.379136 |
| Uqcrc2 | ubiquinol cytochrome c reductase core protein 2 | Mus musculus ubiquinol cytochrome c reductase core protein 2 (Uqcrc2), mRNA [NM_025899] | chr7 | + | mm\|7qF2 | ENSMUST00000033176 | NM_025899 | NM_025899 | Mm.334206 |
| Uqcrq | ubiquinol-cytochrome c reductase, complex III subunit VII | Mus musculus ubiquinol-cytochrome c reductase, complex III subunit VII (Uqcrq), transcript variant 1, mRNA [NM_025352] | chr11 | - | mm\|11qB1.3 | ENSMUST00000109019 | NM_025352 | NM_025352 | Mm.251621 |
| Urb1 | URB1 ribosome biogenesis 1 homolog (S. cerevisiae) | Mus musculus URB1 ribosome biogenesis 1 homolog (S. cerevisiae) (Urb1), mRNA [NM_029497] | chr16 | - | mm\|16qC3.3 | ENSMUST00000140920 | NM_029497 | NM_029497 | Mm.328688 |
| Urgcp | upregulator of cell proliferation | Mus musculus upregulator of cell proliferation (Urgcp), transcript variant 2, mRNA [NM_001077661] | chr11 | - | mm\|11qA1 | ENSMUST00000118076 | NM_001077661 | NM_001077661 | Mm.271657 |
| Usf2-ps1 |  |  |  |  |  |  |  |  |  |
| Uso1 | USO1 vesicle docking factor | Mus musculus USO1 vesicle docking factor (Uso1), mRNA [NM_019490] | chr5 | + | mm\|5qE2 | ENSMUST00000202362 | NM_019490 | NM_019490 | Mm.15868 |
| Usp39 | ubiquitin specific peptidase 39 | Mus musculus ubiquitin specific peptidase 39 (Usp39), mRNA [NM_138592] | chr6 | - | mm\|6qC1 | ENSMUST00000154799 | NM_138592 | NM_138592 | Mm.281900 |
| Usp45 | ubiquitin specific petidase 45 | Mus musculus ubiquitin specific petidase 45 (Usp45), transcript variant 1, mRNA [NM_152825] | chr4 | + | mm\|4qA3 | ENSMUST00000040429 | NM_152825 | NM_152825 | Mm.154306 |
| Usp50 | ubiquitin specific peptidase 50 | Mus musculus ubiquitin specific peptidase 50 (Usp50), mRNA [NM_029163] | chr2 | - | mm\|2qF1 | ENSMUST00000130356 | NM_029163 | NM_029163 | Mm.389373 |
| Usp8 | ubiquitin specific peptidase 8 | Mus musculus ubiquitin specific peptidase 8 (Usp8), transcript variant 1, mRNA [NM_001252580] | chr2 | + | mm\|2qF1 | ENSMUST00000138859 | NM_001252580 | NM_001252580 | Mm.272629 |
| Uxt | ubiquitously expressed transcript | Mus musculus ubiquitously expressed transcript (Uxt), mRNA [NM_013840] | chrX | - | mm\|XqA1.3 | ENSMUST00000001162 | NM_013840 | NM_013840 | Mm.34779 |
| Vars | valyl-tRNA synthetase | Mus musculus valyl-tRNA synthetase (Vars), mRNA [NM_011690] | chr17 | + | mm\|17qB1 | ENSMUST00000173584 | NM_011690 | NM_011690 | Mm.28420 |
| Vdac3 | voltage-dependent anion channel 3 | Mus musculus voltage-dependent anion channel 3 (Vdac3), transcript variant 1, mRNA [NM_001198998] | chr8 | - | mm\|8qA2 | ENSMUST00000009036 | NM_001198998 | NM_001198998 | Mm.227704 |
| Vegfd | vascular endothelial growth factor D | Mus musculus vascular endothelial growth factor D (Vegfd), transcript variant 1, mRNA [NM_010216] | chrX | + | mm\|XqF5 | ENSMUST00000033751 | NM_010216 | NM_010216 | Mm.297978 |
| Vldlr | very low density lipoprotein receptor | Mus musculus very low density lipoprotein receptor (Vldlr), transcript variant 1, mRNA [NM_013703] | chr19 | + | mm\|19qC1 | ENSMUST00000164509 | NM_013703 | NM_013703 | Mm.4141 |
| Vnn1 | vanin 1 | Mus musculus vanin 1 (Vnn1), mRNA [NM_011704] | chr10 | + | mm\|10qA4 | ENSMUST00000041416 | NM_011704 | NM_011704 | Mm.27154 |
| Vta1 | vesicle (multivesicular body) trafficking 1 | Mus musculus vesicle (multivesicular body) trafficking 1 (Vta1), mRNA [NM_025418] | chr10 | - | mm\|10qA2 | ENSMUST00000133927 | NM_025418 | NM_025418 | Mm.227983 |
| Wasf2 | WAS protein family, member 2 | Mus musculus WAS protein family, member 2 (Wasf2), mRNA [NM_153423] | chr4 | + | mm\|4qD2.3 | ENSMUST00000105912 | NM_153423 | NM_153423 | Mm.23566 |
| Washc4 | WASH complex subunit 4 | Mus musculus WASH complex subunit 4 (Washc4), mRNA [NM_001033375] | chr10 | + | mm\|10qC1 | ENSMUST00000038388 | NM_001033375 | NM_001033375 | Mm.491194 |
| Washc5 | WASH complex subunit 5 | Mus musculus WASH complex subunit 5 (Washc5), mRNA [NM_153548] | chr15 | - | mm\|15qD1 | ENSMUST00000022976 | NM_153548 | NM_153548 | Mm.218665 |
| Wdr45b | WD repeat domain 45B | Mus musculus WD repeat domain 45B (Wdr45b), mRNA [NM_025793] | chr11 | - | mm\|11qE2 | ENSMUST00000106110 | NM_025793 | NM_025793 | Mm.103986 |
| Wdr75 | WD repeat domain 75 | Mus musculus WD repeat domain 75 (Wdr75), mRNA [NM_028599] | chr1 | + | mm\|1qC1.1 | ENSMUST00000186308 | NM_028599 | NM_028599 | Mm.270376 |
| Wwtr1 | WW domain containing transcription regulator 1 | Mus musculus WW domain containing transcription regulator 1 (Wwtr1), transcript variant 2, mRNA [NM_133784] | chr3 | - | mm\|3qD | ENSMUST00000120977 | NM_133784 | NM_133784 | Mm.405029 |
| Xpo1 | exportin 1 | Mus musculus exportin 1 (Xpo1), transcript variant 1, mRNA [NM_134014] | chr11 | + | mm\|11qA3.2 | ENSMUST00000102870 | NM_134014 | NM_134014 | Mm.217547 |
| Yars | tyrosyl-tRNA synthetase | Mus musculus tyrosyl-tRNA synthetase (Yars), mRNA [NM_134151] | chr4 | + | mm\|4qD2.2 | ENSMUST00000106054 | NM_134151 | NM_134151 | Mm.145488 |
| Ybx1 | Y box protein 1 | Mus musculus Y box protein 1 (Ybx1), mRNA [NM_011732] | chr4 | - | mm\|4qD2.1 | ENSMUST00000145976 | NM_011732 | NM_011732 | Mm.258204 |
| Ybx3 | Y box protein 3 | Mus musculus Y box protein 3 (Ybx3), transcript variant 1, mRNA [NM_139117] | chr6 | - | mm\|6qF3 | ENSMUST00000032309 | NM_139117 | NM_139117 | Mm.458000 |
| Yipf6 | Yip1 domain family, member 6 | Mus musculus Yip1 domain family, member 6 (Yipf6), mRNA [NM_207633] | chrX | + | mm\|XqC3 | ENSMUST00000054697 | NM_207633 | NM_207633 | Mm.212290 |
| Ykt6 | YKT6 v-SNARE homolog (S. cerevisiae) | Mus musculus YKT6 v-SNARE homolog (S. cerevisiae) (Ykt6), mRNA [NM_019661] | chr11 | + | mm\|11qA1 | ENSMUST00000002818 | NM_019661 | NM_019661 | Mm.294821 |
| Ywhae | tyrosine 3-monooxygenase/tryptophan 5-monooxygenase activation protein, epsilon polypeptide | Mus musculus tyrosine 3-monooxygenase/tryptophan 5-monooxygenase activation protein, epsilon polypeptide (Ywhae), mRNA [NM_009536] | chr11 | + | mm\|11qB5 | ENSMUST00000067664 | NM_009536 | NM_009536 | Mm.234700 |
| Zbtb44 | zinc finger and BTB domain containing 44 | Mus musculus zinc finger and BTB domain containing 44 (Zbtb44), transcript variant a, mRNA [NM_001115130] | chr9 | + | mm\|9qA4 | ENSMUST00000167346 | NM_001115130 | NM_001115130 | Mm.239355 |
| Zc2hc1a | zinc finger, C2HC-type containing 1A | Mus musculus zinc finger, C2HC-type containing 1A (Zc2hc1a), mRNA [NM_173181] | chr3 | + | mm\|3qA1 | ENSMUST00000192835 | NM_173181 | NM_173181 | Mm.332366 |
| Zc3h14 | zinc finger CCCH type containing 14 | Mus musculus zinc finger CCCH type containing 14 (Zc3h14), transcript variant 1, mRNA [NM_029334] | chr12 | + | mm\|12qE | ENSMUST00000222146 | NM_029334 | NM_029334 | Mm.25549 |
| Zfand5 | zinc finger, AN1-type domain 5 | Mus musculus zinc finger, AN1-type domain 5 (Zfand5), mRNA [NM_009551] | chr19 | + | mm\|19qB | ENSMUST00000025659 | NM_009551 | NM_009551 | Mm.292405 |
| Zfp106 | zinc finger protein 106 | Mus musculus zinc finger protein 106 (Zfp106), mRNA [NM_011743] | chr2 | - | mm\|2qE5 | ENSMUST00000055241 | NM_011743 | NM_011743 | Mm.485295 |
| Zfp7 | zinc finger protein 7 | Mus musculus zinc finger protein 7 (Zfp7), mRNA [NM_145916] | chr15 | + | mm\|15qD3 | ENSMUST00000023179 | NM_145916 | NM_145916 | Mm.38948 |
| Zfp938 | zinc finger protein 938 | Mus musculus zinc finger protein 938 (Zfp938), mRNA [NM_001105557] | chr10 | - | mm\|10qC1 | ENSMUST00000156218 | NM_001105557 | NM_001105557 | Mm.393147 |
| Zfp948 | zinc finger protein 948 | Mus musculus zinc finger protein 948 (Zfp948), mRNA [NM_001002008] | chr17 | + | mm\|17qA3.2 | ENSMUST00000088787 | NM_001002008 | NM_001002008 | Mm.441097 |
| Zfyve16 | zinc finger, FYVE domain containing 16 | Mus musculus zinc finger, FYVE domain containing 16 (Zfyve16), mRNA [NM_173392] | chr13 | - | mm\|13qC3 | ENSMUST00000156586 | NM_173392 | NM_173392 | Mm.259585 |
| Zkscan5 | zinc finger with KRAB and SCAN domains 5 | Mus musculus zinc finger with KRAB and SCAN domains 5 (Zkscan5), transcript variant 1, mRNA [NM_016683] | chr5 | + | mm\|5qG2 | ENSMUST00000031601 | NM_016683 | NM_016683 | Mm.332842 |
| Zw10 | zw10 kinetochore protein | Mus musculus zw10 kinetochore protein (Zw10), mRNA [NM_012039] | chr9 | + | mm\|9qA5.3 | ENSMUST00000034803 | NM_012039 | NM_012039 | Mm.24791 |
| Zyg11b | zyg-ll family member B, cell cycle regulator | Mus musculus zyg-ll family member B, cell cycle regulator (Zyg11b), mRNA [NM_001033634] | chr4 | - | mm\|4qC7 | ENSMUST00000043616 | NM_001033634 | NM_001033634 | Mm.426241 |
