# Supplementary material for: Targeting metabolic vulnerabilities: REV-ERB agonist SR9009 potentiates sorafenib efficacy in liver cancer
Source: Cell Death Discov. 2026 Jan 19;12:86. doi: 10.1038/s41420-026-02940-3 (PMC12877144; doi:10.1038/s41420-026-02940-3)

Full lenght Western Blot employed for Figure 2c

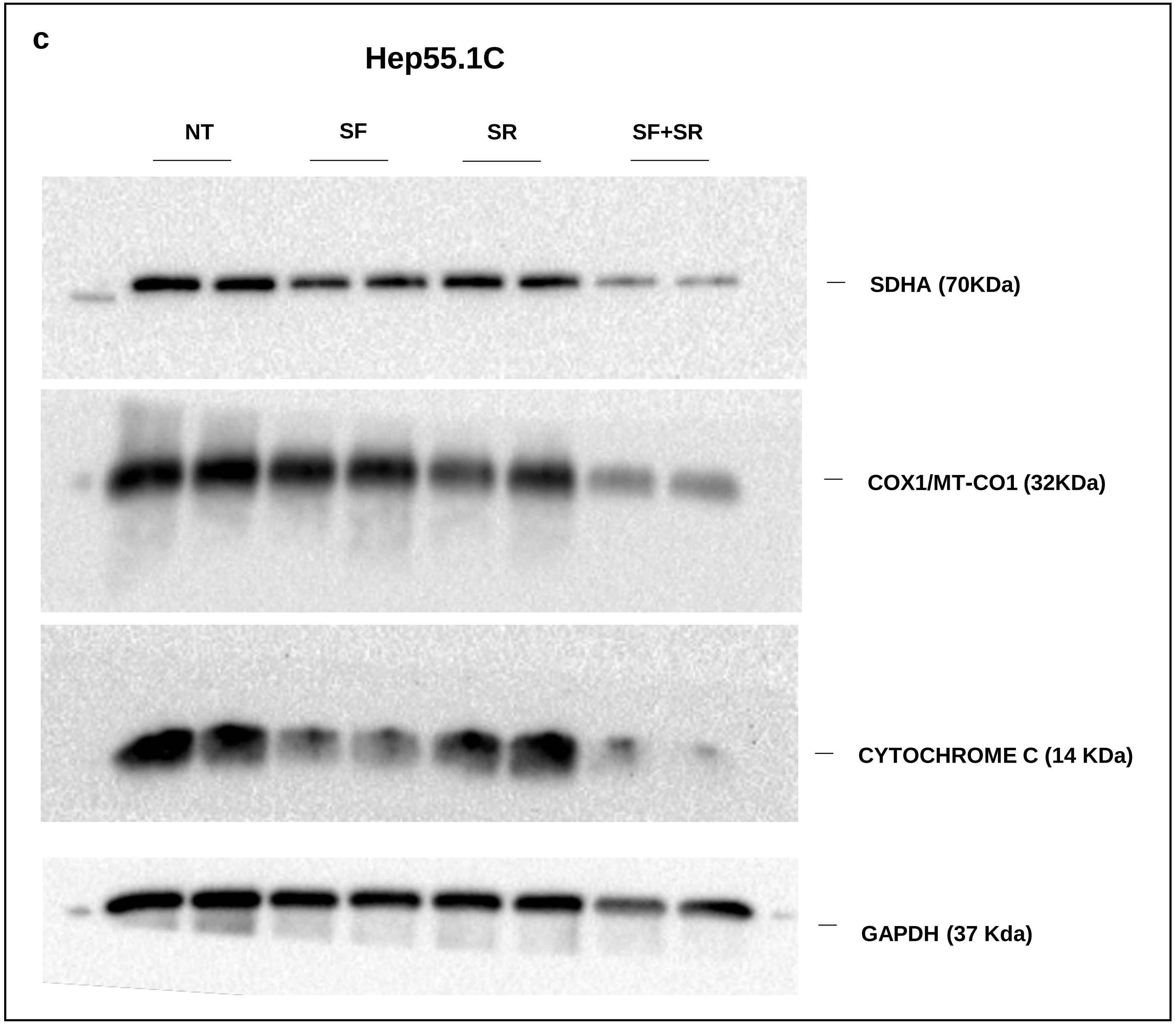

Full length Western Blot employed for Figure 4b and Supplementary Fig. 11

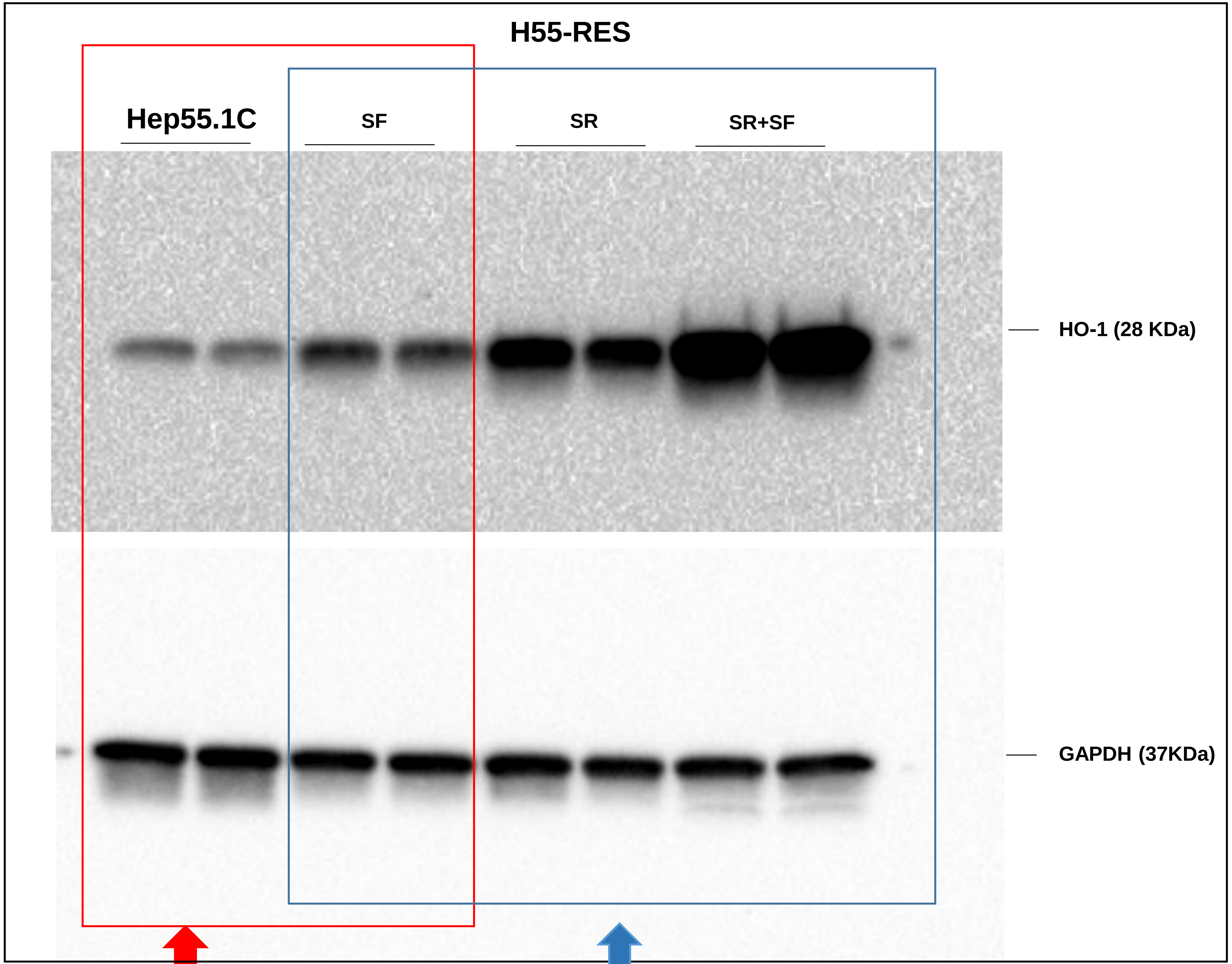

Included in final Suppl Figure 11

Included in final Figure 4

Full lenght Western Blot employed for Supplementary Figure 6a

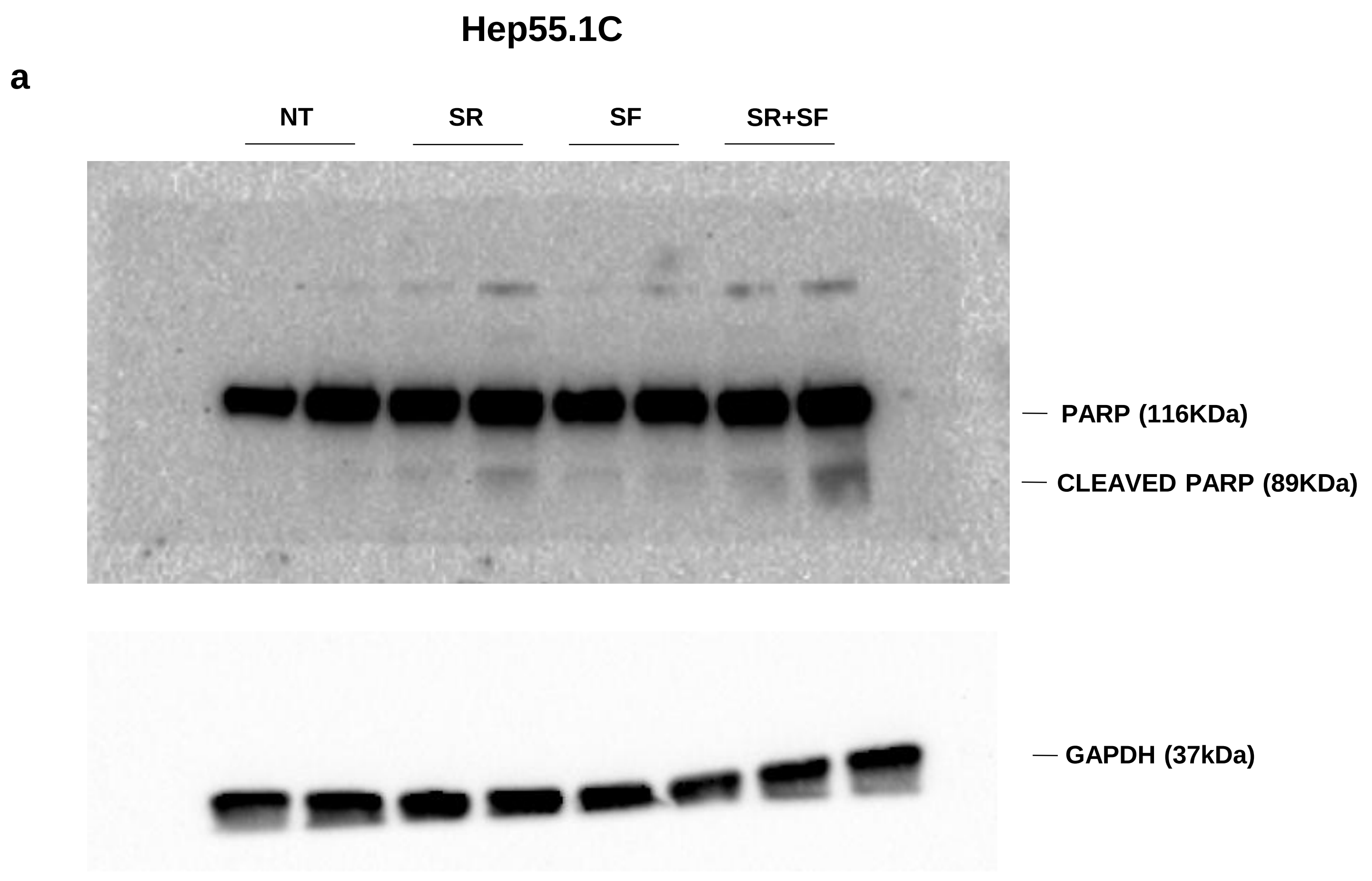

Full lenght Western Blot employed for Supplementary Figure 6b

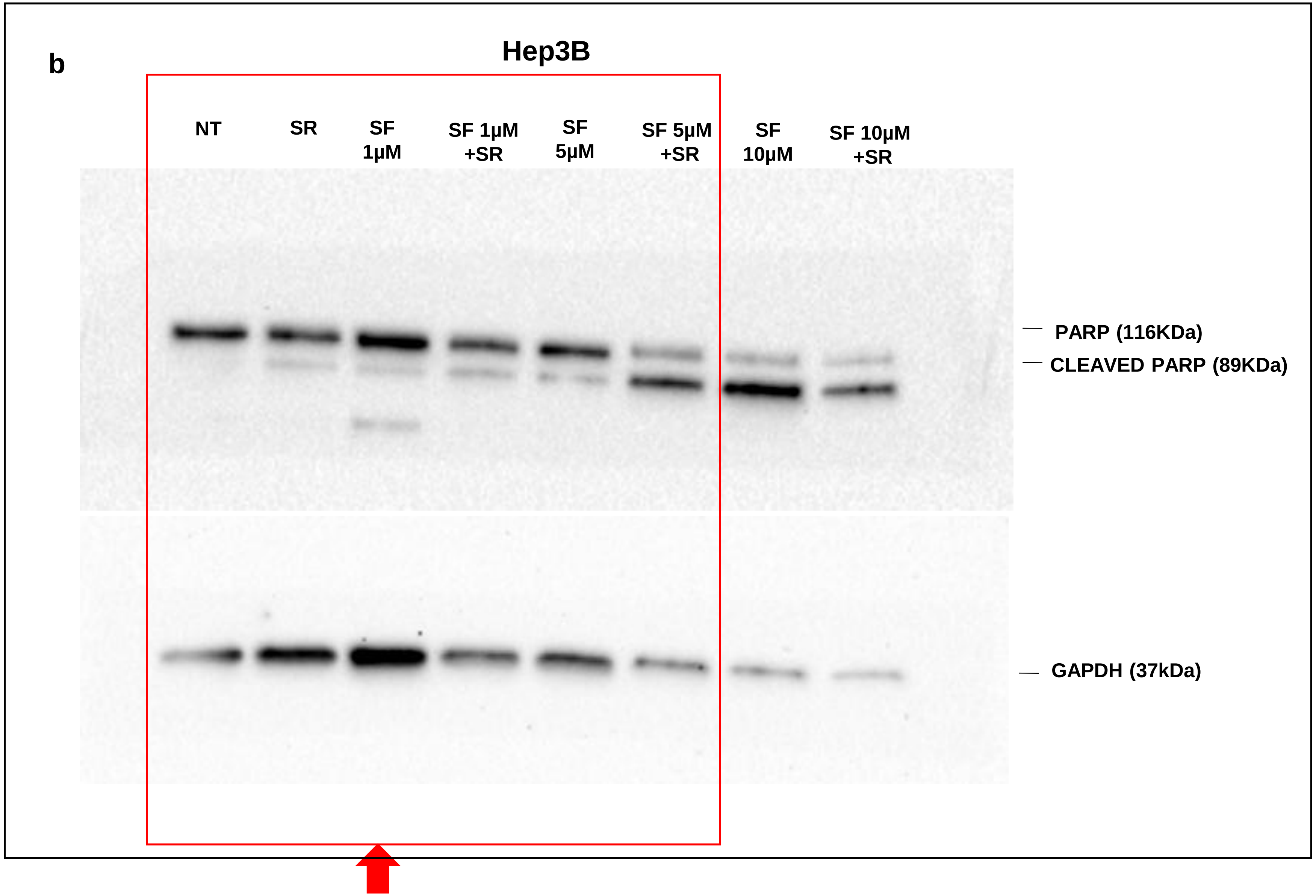

Included in final Supplementary Figure 6

Full length Western Blot employed for Supplementary Figure 12

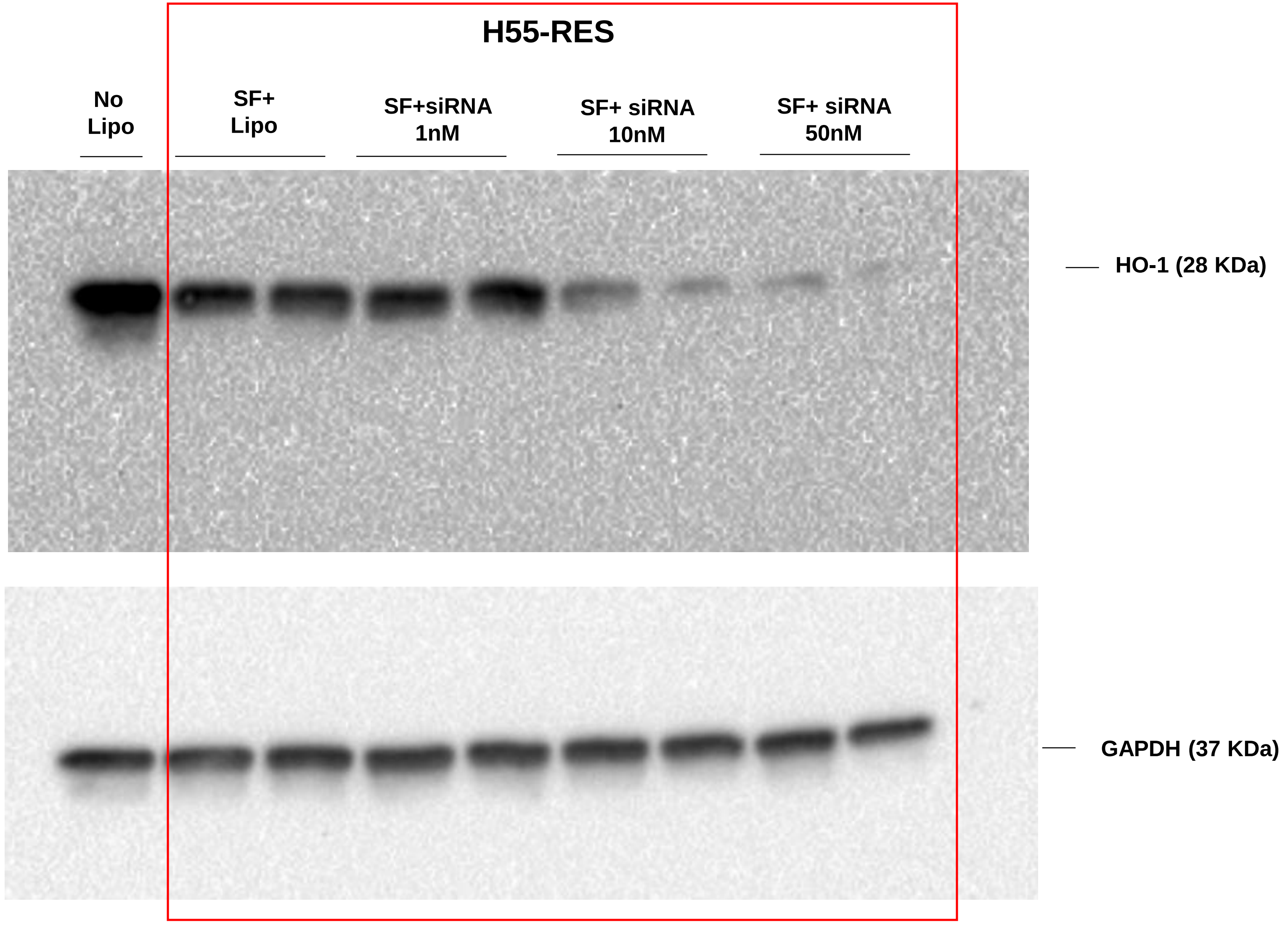

Included in final Supplementary Figure 12

Full lenght Western Blot employed for Supplementary Figure 13

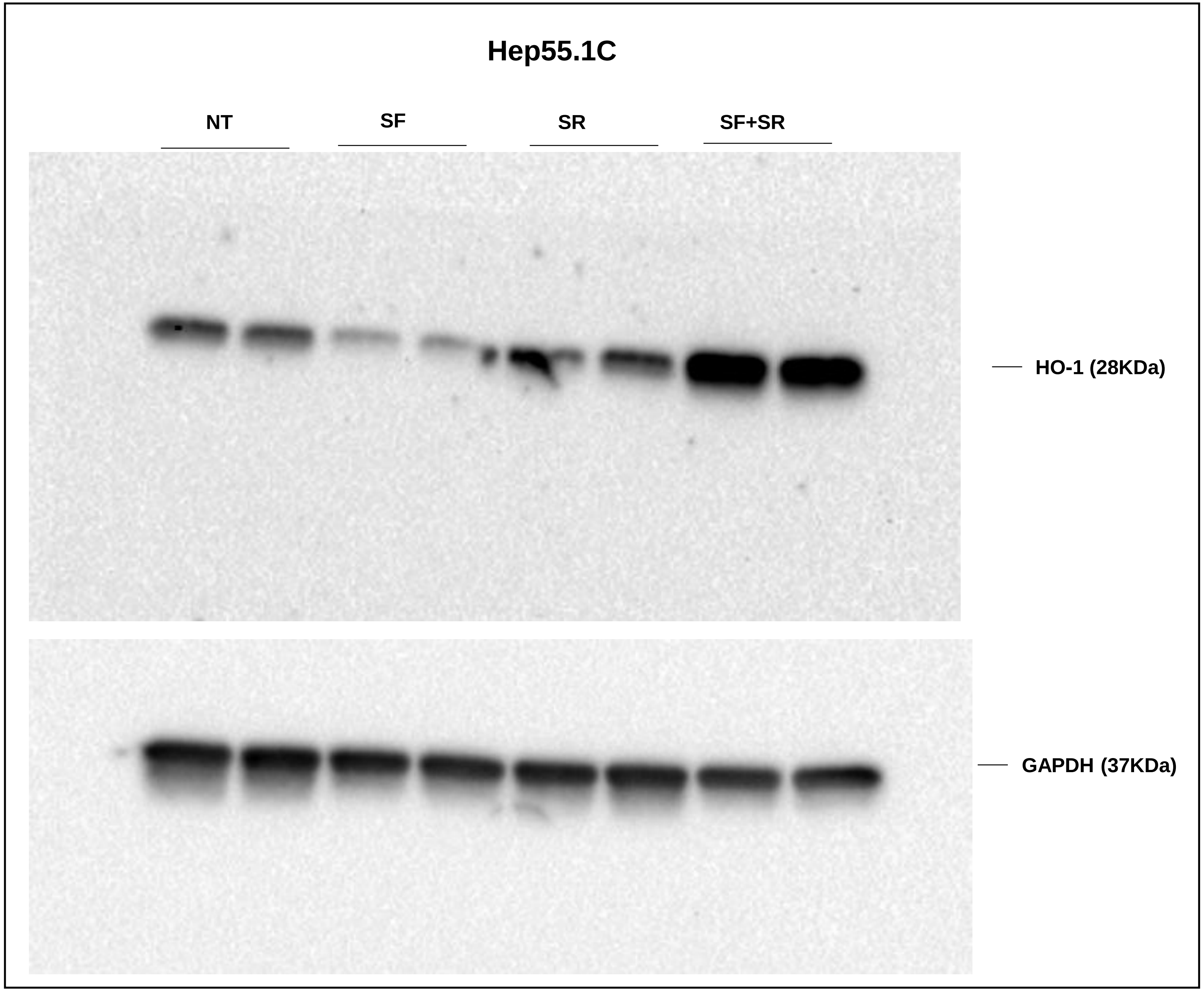

Full length Western Blot employed for Supplementary Figure 14

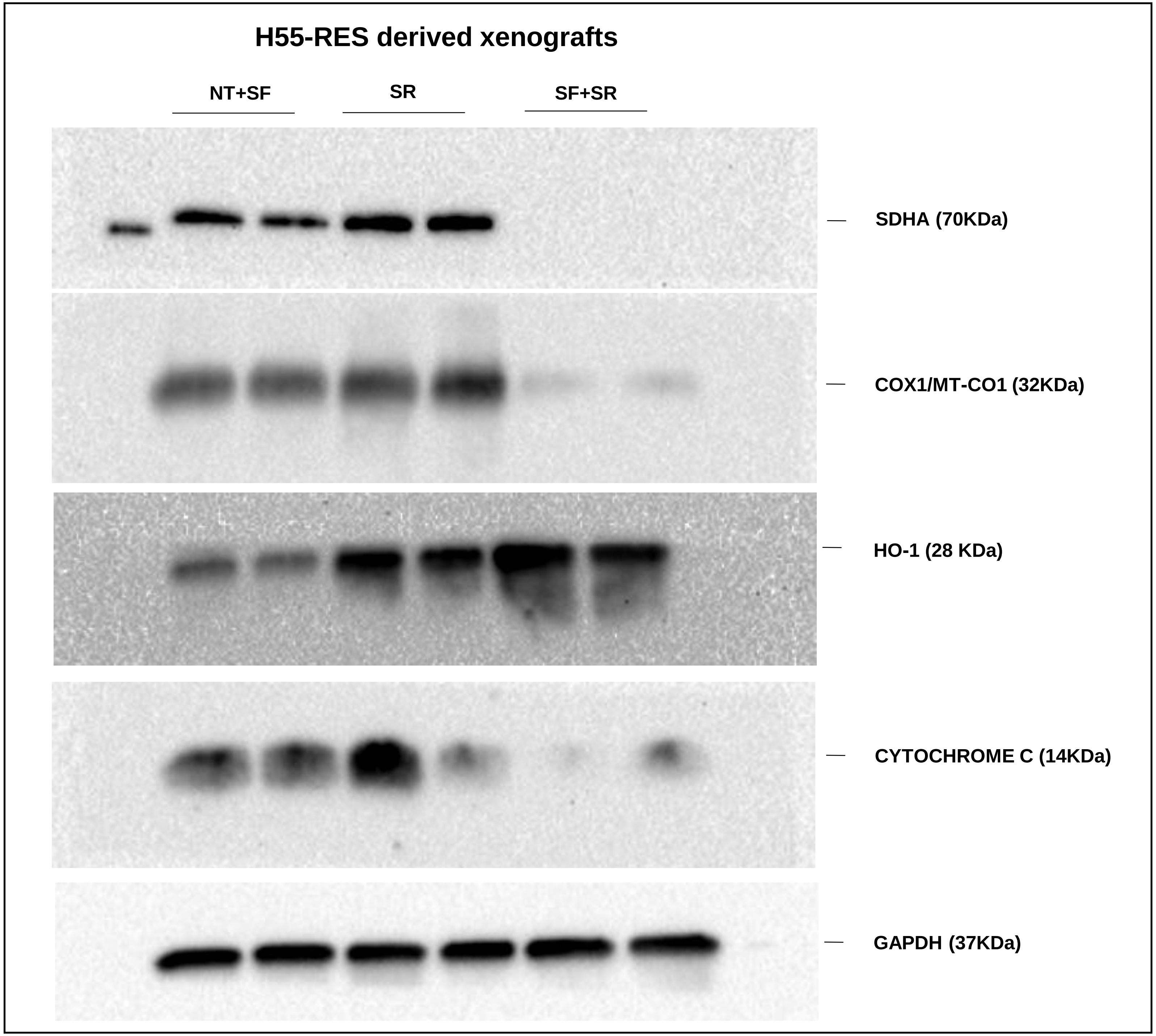

Supplement: Supplementary file 4 — Full length Western Blot [file 41420_2026_2940_MOESM4_ESM.pdf]
